# Novel lung imaging biomarkers and skin gene expression subsetting in dasatinib treatment of systemic sclerosis-associated interstitial lung disease

**DOI:** 10.1371/journal.pone.0187580

**Published:** 2017-11-09

**Authors:** Viktor Martyanov, Grace-Hyun J. Kim, Wendy Hayes, Shuyan Du, Bishu J. Ganguly, Oumar Sy, Sun Ku Lee, Galina S. Bogatkevich, Gary L. Schieven, Elena Schiopu, Roberta Gonçalves Marangoni, Jonathan Goldin, Michael L. Whitfield, John Varga

**Affiliations:** 1 Geisel School of Medicine at Dartmouth, Hanover, NH, United States of America; 2 David Geffen School of Medicine at UCLA, University of California, Los Angeles, CA, United States of America; 3 Bristol-Myers Squibb, Princeton, NJ, United States of America; 4 Medical University of South Carolina, Charleston, SC, United States of America; 5 University of Michigan Health System, Ann Arbor, MI, United States of America; 6 Northwestern Scleroderma Program, Feinberg School of Medicine, Chicago, IL, United States of America; Keio University, JAPAN

## Abstract

**Background:**

There are no effective treatments or validated clinical response markers in systemic sclerosis (SSc). We assessed imaging biomarkers and performed gene expression profiling in a single-arm open-label clinical trial of tyrosine kinase inhibitor dasatinib in patients with SSc-associated interstitial lung disease (SSc-ILD).

**Methods:**

Primary objectives were safety and pharmacokinetics. Secondary outcomes included clinical assessments, quantitative high-resolution computed tomography (HRCT) of the chest, serum biomarker assays and skin biopsy-based gene expression subset assignments. Clinical response was defined as decrease of >5 or >20% from baseline in the modified Rodnan Skin Score (MRSS). Pulmonary function was assessed at baseline and day 169.

**Results:**

Dasatinib was well-tolerated in 31 patients receiving drug for a median of nine months. No significant changes in clinical assessments or serum biomarkers were seen at six months. By quantitative HRCT, 65% of patients showed no progression of lung fibrosis, and 39% showed no progression of total ILD. Among 12 subjects with available baseline and post-treatment skin biopsies, three were improvers and nine were non-improvers. Improvers mapped to the fibroproliferative or normal-like subsets, while seven out of nine non-improvers were in the inflammatory subset (p = 0.0455). Improvers showed stability in forced vital capacity (FVC) and diffusing capacity for carbon monoxide (D_L_CO), while both measures showed a decline in non-improvers (p = 0.1289 and p = 0.0195, respectively). Inflammatory gene expression subset was associated with higher baseline HRCT score (p = 0.0556). Non-improvers showed significant increase in lung fibrosis (p = 0.0313).

**Conclusions:**

In patients with SSc-ILD dasatinib treatment was associated with acceptable safety profile but no significant clinical efficacy. Patients in the inflammatory gene expression subset showed increase in skin fibrosis, decreasing pulmonary function and worsening lung fibrosis during the study. These findings suggest that target tissue-specific gene expression analyses can help match patients and therapeutic interventions in heterogeneous diseases such as SSc, and quantitative HRCT is useful for assessing clinical outcomes.

**Trial registration:**

Clinicaltrials.gov NCT00764309

## Introduction

Systemic sclerosis (SSc) is a clinically heterogeneous orphan disease characterized by autoimmunity, widespread microangiopathy and multi-organ fibrosis [1,2]. Progressive fibrosis in the skin and lungs accounts for the significant morbidity and mortality of SSc [3]. In the skin, excessive matrix deposition causes stiffening and tightening, as assessed by the modified Rodnan Skin Score (MRSS), while in the lungs, fibrosis causes respiratory insufficiency associated with restrictive physiologic changes and interstitial lung disease (ILD) detected by high resolution computed tomography (HRCT) [4,5]. Despite significant recent advances toward elucidating the pathogenesis and uncovering novel targets, effective disease-modifying therapy for SSc remains elusive [6].

The profibrotic cytokines transforming growth factor beta (TGF-β) and platelet-derived growth factor (PDGF) are established as key pathogenic mediators of skin and lung fibrosis in SSc [7,8]. Tyrosine kinases are implicated in profibrotic intracellular signaling cascades downstream of both TGF-β and PDGF receptors, and therefore represent potential therapeutic targets in SSc [9–11]. Significant challenges remain in the development of effective therapies for SSc [6]. These include marked patient-to-patient heterogeneity in clinical features and molecular drivers of disease, variable trajectories for the rate of disease progression in different organs even for the same individual, unpredictable clinical outcomes and the lack of robust prognostic and diagnostic biomarkers. Transcriptome analysis of target organs such as skin and lungs, and quantitative assessment of the extent of radiological lung involvement on HRCT represent promising novel strategies in this regard [12]. In particular, HRCT has been shown to be predictive of survival in SSc, and changes in texture-based quantitative measures are potential surrogate biomarkers of treatment response in clinical trials [13].

The broad-spectrum tyrosine kinase inhibitor (TKI) dasatinib was originally developed to target breakpoint cluster region-Abelson tyrosine-protein kinase 1 (*BCR-Abl*) and is now widely used in the treatment of chronic myeloid leukemia (CML) [14–17]. The potent inhibitory effects of dasatinib on c-Abl, PDGF receptor and Src kinase activities suggest that it may have anti-fibrotic efficacy [18,19]. Indeed, in preclinical models dasatinib was shown to mitigate bleomycin-induced dermal fibrosis [18] and reduce lung inflammation and silica-induced pulmonary fibrosis [20] in mice. Furthermore, dasatinib inhibits T-cell receptor signaling and the production of inflammatory and fibrotic cytokines [21–23]. These considerations prompted us to undertake an open-label clinical trial to evaluate the safety of dasatinib in SSc patients with associated ILD. As secondary and exploratory outcomes, we evaluated the utility of radiologic texture-based quantitative imaging biomarkers, combined with skin-based gene expression profiling, in predicting and monitoring treatment response.

## Materials and methods

### Study protocol

We undertook a multicenter, single-arm, open-label Phase 2a trial (NCT00764309) of dasatinib (Bristol-Myers Squibb, Princeton, NJ) in SSc-ILD. Patients received dasatinib (100 mg) once daily for 6 months (primary endpoint), and were followed for up to 18 additional months to assess longer-term safety. The protocol was approved by the Institutional Review Boards/Independent Ethics Committees from each participating center: Western Institutional Review Board, Olympia, WA, USA; University of Michigan IRBMED, Ann Arbor, MI, USA; Lifespan Institutional Review Board, Providence, RI, USA; Mayo Clinic Institutional Review Board, Rochester, MN, USA and MUSC Institutional Review Board for Human Research, Charleston, SC, USA. Written informed consent was received from each patient before study entry. Date range for participant recruitment and follow-up was January 2009 through April 2011. CONSORT checklist is provided as S1 Checklist and complete study protocol is included in S1 File.

Eligible patients were ≥18 years old with the diagnosis of diffuse cutaneous SSc (dcSSc), according to the American College of Rheumatology criteria and the LeRoy classification [24,25]. Inclusion criteria included disease duration (defined as the interval from first non-Raynaud disease manifestation) ≤ 3 years, and MRSS ≥15 [26]. Patients had to have functional evidence of pulmonary involvement (defined as forced vital capacity (FVC) between 45% and 80% of predicted normal and/or diffusing capacity for carbon monoxide (D_L_CO) between 30% and 70% of predicted normal) and/or HRCT evidence for ILD (defined as ground glass opacities, inter-and intra-lobular septal thickening, traction bronchiectasis, and/or honeycombing). Treatment with methotrexate, cyclophosphamide or D-penicillamine was discontinued at least 12 weeks prior to enrollment, while steroids (at a dose <10 mg or equivalent of prednisone/day) could be continued at a stable dose. The full list of inclusion and exclusion criteria is shown in S1 Table.

### Safety and pharmacokinetics

Physical examinations and assessment of vital signs and laboratory values were completed at screening and at day 1, 15, 29, and monthly thereafter throughout the course of the study. Blood samples were collected for pharmacokinetics (PK) analysis on day 15 pre-dose and 0.5, 1, 1.5, 2, 3, 4, 6 and 8 hours post-dose. Plasma samples were analyzed by liquid chromatography tandem mass spectrometry and pharmacokinetic parameters were estimated using non-compartmental analysis [27].

### Efficacy assessments

Safety was the primary outcome. Efficacy outcomes included change at day 169 compared to baseline in the following parameters: MRSS, pulmonary function test (PFT) results, the Mahler Transitional Dyspnea Index (TDI), and HRCT of the lungs. HRCT scans were performed at baseline and at 6-month follow-up. A standardized HRCT protocol was followed at all sites and a rigorous ongoing quality control program was used to ensure patient compliance with breath-hold and acquisition guidelines [28]. A computer-aided diagnosis (CAD) system was used to segment the lungs and quantitate the extent and changes in parenchymal abnormalities [29,30]. This technique utilizes a texture feature classification that has been trained to allow quantitation (Q) of four parameters of ILD: lung fibrosis (QLF), ground glass (QGG), honeycomb cysts (QHC), and a combination of all three, quantitative interstitial lung disease (QILD) [30,31]. Thresholds of 3% changes for the most severe lobe and 2% changes for the whole lung were considered as meaningful changes [30]. In addition, all HRCT studies were also scored visually using a modified Kazerooni scoring system [32] that utilizes a standardized Likert scale (0 = absent; 1 = 1–5%; 2 = 6–25%; 3 = 26–50%; 4 = 51–75%; and 5 = 76–100%) for each component (ground glass opacities, fibrosis and honeycomb cysts). Scoring was performed by two blinded readers. Discordant reads were reviewed by a consensus reading; the majority score was recorded. All HRCT scans were read with respect to each lung, and each lobe. Comparison of baseline and 6-month HRCT scans was carried out by randomly assigning one of the two scans as “scan A” and the other one as “scan B” and each component and lobe was scored as better, same or worse by the two readers [33].

### Serum markers

Serum levels of Krebs von den Lungen-6 (KL-6), surfactant protein D (SP-D), a B cell proliferation-inducing ligand (APRIL) and adiponectin were determined at baseline and serially during the trial by enzyme-linked immunosorbent assay (ELISA) using kits from BioVendor or from Millipore (EZHMWA-64K and EZHADP-61K).

### Skin biopsies

Biopsies of skin on the dorsal forearms (10 cm distal to the olecranon) were performed at baseline and on day 169. Repeat biopsies were performed within 1 cm of the initial biopsy site.

### RNA isolation, labeling and hybridization

RNA was isolated from skin biopsies by RNeasy Fibrous Tissue Mini Kit (Qiagen), with on-column DNase digestion. RNA quality was monitored by Agilent 2100 Bioanalyzer (Agilent Technologies). RNA quantity was measured with NanoDrop (Thermo Fisher Scientific). RNA samples were arrayed into 96-Well Microtiter Microplates (Thermo Fisher Scientific). 50 ng of skin RNA for each sample was amplified and labeled with WT-Ovation Pico System and Encore Biotin Module (NuGEN Technologies). 2.5 μg of labeled cDNA was hybridized on GeneChip HT HG-U133A Array Plate (Affymetrix).

### Chip quality check and expression data generation

Scanned images were visually inspected and chip quality report was generated by Expression Console Software (Affymetrix). Image data were processed using Robust Multichip Average (RMA) [34] to determine specific hybridizing signal for each gene. CEL files were pre-processed via RMA generating a combined dataset with 45 samples and 22,277 probes. The gene expression data have been uploaded to NCBI GEO (GSE79387).

### Intrinsic gene expression subset assignment and pathway enrichment analysis

Intrinsic genes were determined as previously described [35]. Differential gene expression and pathway enrichment analyses were performed as recently described [36]. Briefly, differentially expressed genes were determined using GenePattern [37] module ComparativeMarkerSelection [38] and differentially expressed pathways were identified via Gene Set Enrichment Analysis [39,40] run vs. Hallmark database [41].

### Statistical analysis

All patients who received ≥1 dose of study drug were included in the safety analysis. Baseline and change from baseline values for each study visit were summarized using descriptive statistics for PFTs, TDI, and MRSS. The TDI scores represent either an improvement (positive) or worsening (negative). Serum adiponectin levels at baseline and at the end of the study were compared with the Wilcoxon signed-rank test. For HRCT fibrosis scoring, changes relative to baseline for each lobe type and overall were summarized for subjects with at least one follow-up paired scan (note that two patients had less than 6-month follow-up). Changes in quantitative scores were classified as “not worse” (if >3 or >2% decrease from baseline score or stable within ±3% or ±2% changes in most severe baseline lobe or whole lung, respectively), or worse (if >3 or >2% increase from baseline score in most severe baseline lobe or whole lung, respectively) [30,42]. Spearman rank correlations were used to test associations among radiological data, PFT, MRSS, and serum markers. Kruskal-Wallis rank tests were used to compare the continuous CAD or FVC scores by the categorical visual assessment.

Gene signature centroids were generated by averaging expression values of each gene across all given samples (e.g. across all baseline improver samples). Categorical variables were analyzed via Fisher’s exact test. Mann-Whitney and Wilcoxon signed-rank tests were used to compare PFT measures and HRCT quantitative scores between improver/non-improver and baseline/post-treatment groups, respectively. Data were plotted as mean ± standard error of the mean (SEM) scatter plots. Statistical analyses were performed in Stata (version 14.0) and GraphPad Prism (Windows, version 6.05).

## Results

### Patient characteristics

**Fig 1:**
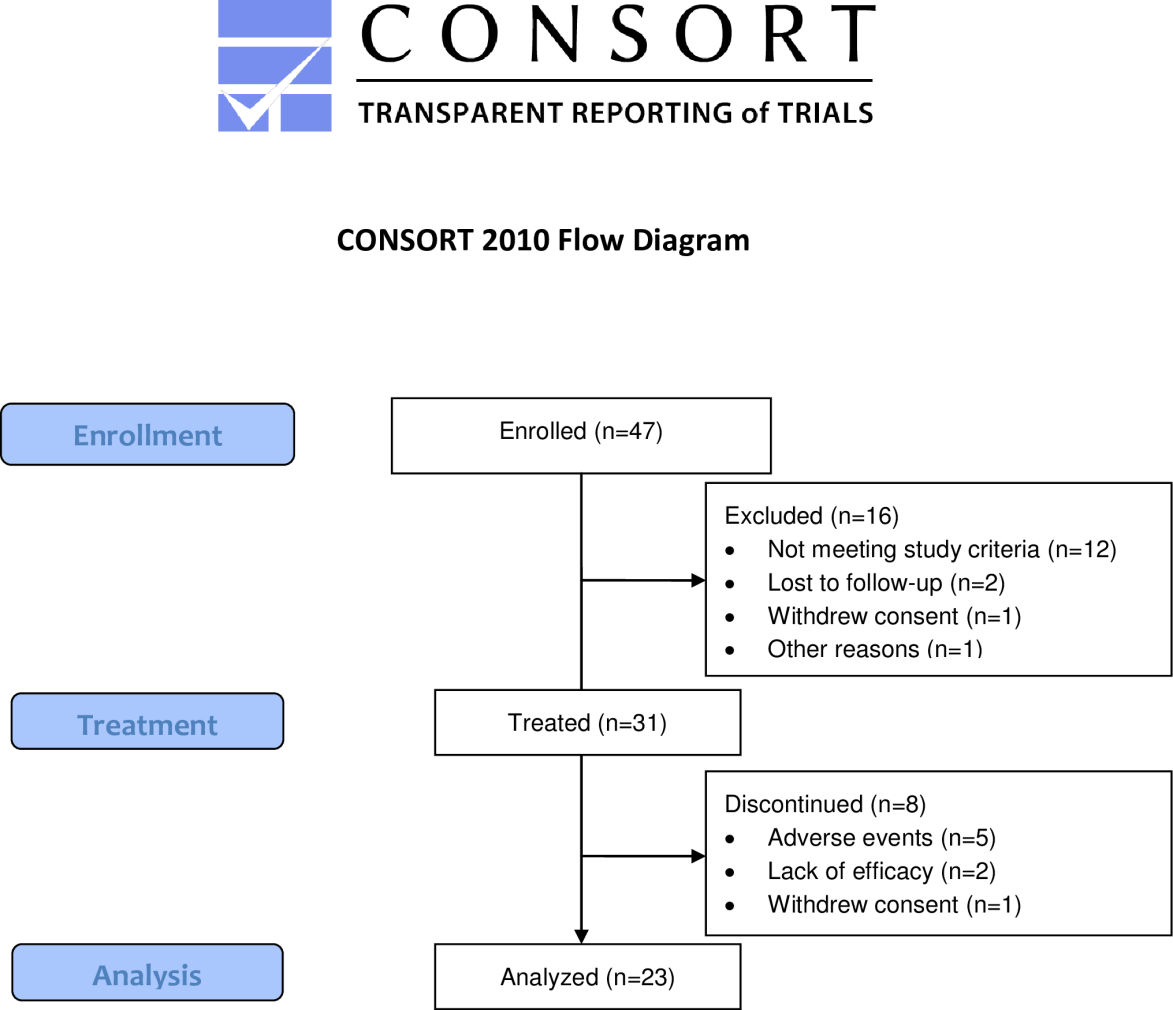
CONSORT flowchart for the study.

Of 47 screened subjects, 31 (66%) received ≥1 dose (100 mg) dasatinib. Baseline demographics and disease characteristics are summarized in Table 1. The mean age was 50.8 years (range 24–77), 67.7% were female, 77.4% were white, and median disease duration from first non-Raynaud disease manifestation was 14.1 months. At baseline, the mean MRSS was 24.5, FVC was 2.9 L, and D_L_CO was 14.2 mL/min/mmHg. Discontinuations from the study before day 169 (primary endpoint) occurred in eight patients, and were due to an adverse event (AE) (n = 5), lack of efficacy (n = 2) or withdrawn consent (n = 1). Patient allocation is provided as CONSORT flowchart in Fig 1.

**Table 1:** Baseline demographics and disease characteristics of SSc patients (n = 31).

Parameter	Value^#^
Mean age, years (range)	50.8 (24–77)
Female, n (%)	21 (67.7)
Aged <65 years, n (%)	26 (83.9)
Race, n (%):	
• White	77.4)
• Black	(9.7)
• American Indian/Alaska native	(3.2)
• Other	3 (9.7)
Median disease duration, months (range) -from the first non-Raynaud disease manifestation	14.1 (1–53)^a^
FVC*	2.9 ± 0.8
D_L_CO^&^	14.2 ± 4.8
MRSS*	24.5 ± 7.7
Serum markers:	
• APRIL^†^	11.4 ± 10.8
• SP-D^†^	± 126.7
• KL-6^†^	1078.0 ± 1038.4
HRCT, whole lung:	
• QGG, %	± 10.6
• QLF, %	± 9.1
• QHC*, %	± 0.4
• Total QILD*, %	24.7 ± 18.0
HRCT, most severe lobe:	
• QGG, %	± 13.3
• QLF, %	± 20.8
• QHC*, %	0.2 ± 0.3
• Total QILD*, %	40.9 ± 26.5

^#^Unless stated otherwise, values are mean ± standard deviation

*n = 30

^&^n = 29

^†^n = 27

^a^27 of 31 (87.1%) treated patients had disease duration ≤ 3 years; 4 of 31 (12.9%) had disease duration >3 years dcSSc, diffuse cutaneous systemic sclerosis; D_L_CO, diffusing capacity for carbon monoxide; FVC, forced vital capacity; MRSS, modified Rodnan skin score; QGG, quantitative ground glass; QLF, quantitative lung fibrosis; QHC, quantitative honeycomb; QILD, quantitative interstitial lung disease.

### Pharmacokinetics summary

In 21 evaluable subjects, the geometric mean maximum concentration (C_max_) and area under the curve (AUC) were 40.1 ng/mL (92% CV) and 119.0 ng•h/mL (73% CV), respectively. The median t_max_ was 1.5 h (range: 0.5–4) and the mean t-half was 2.7 h ± 0.9.

### Safety outcomes

Overall, dasatinib was well tolerated in this cohort of patients with early-stage SSc-ILD. No significant cardiac toxicity was observed. Of a total of 31 reported AEs, diarrhea and nausea were most common (S2 Table). A total of 7 serious AEs were recorded (S3 Table).

### Clinical efficacy outcomes

The change (mean ± standard deviation) in MRSS from baseline to day 169 was –1.8 ± 9.9 (p = 0.1531; n = 21). At follow-up, the change to day 365 was –7.6 ± 4.7 (p = 0.0002; n = 13) and to day 533 was –10.2 ± 4.2 (p = 0.0313; n = 6). For FVC, the results were as follows: at day 169, -0.1 ± 0.4 (p = 0.2064; n = 19); at day 365, -0.2 ± 0.4 (p = 0.0413; n = 15) and at day 533, -0.2 ± 0.2 L (p = 0.0625; n = 6). For D_L_CO, the changes were as follows: at day 169, -0.5 ± 9.1 (p = 0.0090; n = 18); at day 365, -0.3 ± 3.1 (p = 0.3028; n = 15) and at day 533, -2.2 ± 4.0 mL/min/mmHg (p = 0.3125; n = 6).

Matched pairs of baseline and post-treatment skin biopsies were available for 12 subjects. Of these, 3/12 were classified as improvers based on the change in MRSS, showing a >5 point or >20% decrease from baseline to post-treatment MRSS. The remaining subjects (9/12) were classified as non-improvers.

### Overview of quantitative HRCT results

**Fig 2:**
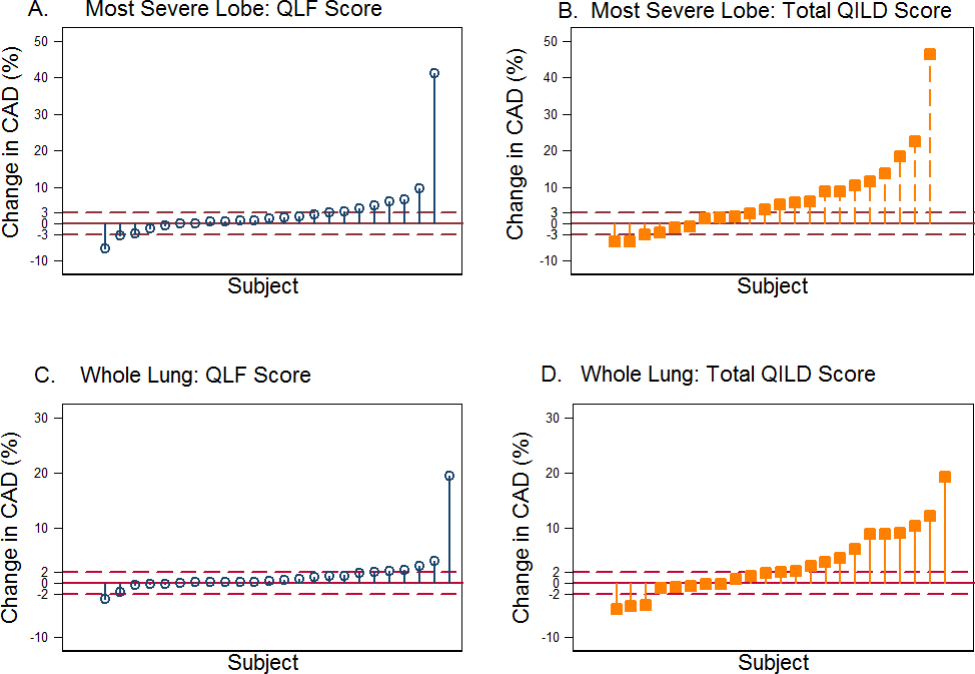
Drop plots of changes in texture-based quantitative scores from baseline using HRCT. (A) QLF and (B) QILD for the most severe lobe. (C) QLF and (D) QILD for the whole lung. Dashed lines of ±3% and ±2% are indicators of thresholds for changes in regional analysis in lobe and whole lung, respectively.

**Fig 3:**
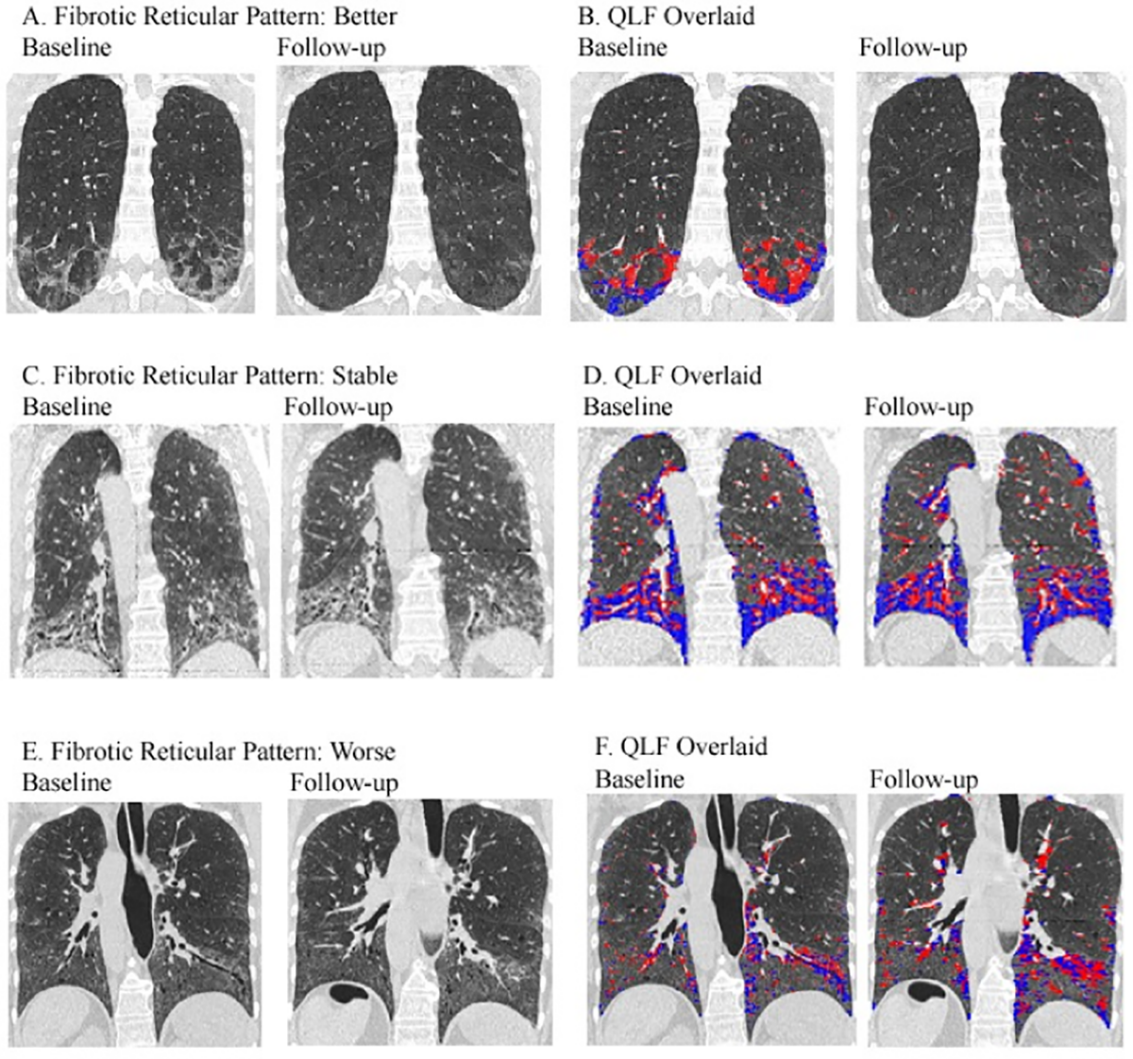
Representative pairs of baseline and follow-up HRCT images. (A) Better case: pulmonary fibrosis (PF) improved and ground glass opacity (GGO) worsened by the visual assessment in whole lung; (B) annotated HRCT of (A) with a CAD system: QLF and QILD in whole lung decreased, by 2.9% (3.9% to 1.0%) and 0.2% (19.9% to 19.7%), respectively. (C) Stable case; (D) annotated CAD of (C): QLF in the worst lobe (lower left) increased by 2% (64% to 66%). In whole lung, QLF and QILD increased, by 2.3% (39.5% to 41.8%) and 1.8% (72.4% to 74.2%), respectively. (E) Worse case: PF worsened and GGO improved; (F) annotated CAD of (E): QLF increased by 6.0% (28% to 34%) in the most severe lobe (lower right) and 1.8% (9.8% to 11.6%) in whole lung. QILD in whole lung was stable (41.3% to 40.5%).

Thirty-one patients patient had baseline ILD evaluation by quantitative HRCT. The mean values for the whole lung were as follows: QLF, 6.6%; QGG, 17.8%; QHC, 0.3%; and QILD, 24.7%. At the most severe lobe, the mean values were as follows: QLF, 16.2%; QGG, 24.4%; QHC, 0.2%; and QILD, 40.9%. For serial evaluation, 23 matched baseline and 6 month follow-up HRCT scans were available for analysis. The HRCT lung volumes showed a significant correlation with PFT-based lung volumes both at baseline (ρ≈ -0.50) and at follow-up (range from ρ = -0.69 to ρ = -0.32) (S4 and S5 Tables). Based on previously described quantitative computed tomography changes in SSc-ILD [30], subjects were classified as either “not worse” (combining patients with stable or decreased score) or “worse” (patients with increased score) at the 6-month follow-up. Using this dichotomous approach, the number of “not worse” subjects in the most severe baseline lobe and whole lung was as follows: QLF, 15 and 17; QILD, 9 and 10, respectively. The number of “worse” subjects was as follows: QLF, 8 and 6; QILD, 14 and 13 (Fig 2). At the most severe lobe, 65% of patients showed no progression by QLF and 39% showed no progression by QILD. Baseline QLF at most severe lobe and whole lung showed strong correlation with visual assessment of extent of lung fibrosis and with change in fibrosis at 6 month follow-up (S1 Fig). HRCT images at baseline and day 169 from representative subjects with reduced, stable, and increased QLF and QILD scores are shown (Fig 3). Weak associations between visual assessment of radiologic ILD and FVC at baseline and day 169 were found (Panels E and F in S1 Fig; p = 0.14 and p = 0.29, respectively).

### Serum marker changes

We measured selected putative SSc biomarkers in the serum. Levels of both KL-6 and SP-D were elevated at baseline, but substantial patient-to-patient variability was seen. They did not show significant change during the 6-month study period (S6 and S7 Tables). In contrast, serum levels of adiponectin, an anti-fibrotic adipokine previously implicated in SSc [43] showed a significant rise post-treatment (p<0.01 for all comparisons; S2 Fig). Further analysis revealed that baseline serum levels of SP-D and KL-6 were significantly correlated with QLF and QILD scores (S4 Table). Associations between QLF and FVC were -0.53 (p = 0.0025) at baseline and -0.36 (p = 0.10) for changes in the whole lung. Associations between QILD and FVC were -0.48 (p = 0.0077) at baseline and -0.56 (p = 0.0083) for changes in the whole lung. Changes in SP-D were significantly correlated with changes in QLF scores at both the most severe lobe and whole lung (ρ = 0.67 (p = 0.0001), ρ = 0.68 (p = 0.0001) at baseline; ρ = 0.59 (p = 0.0035), ρ = 0.47 (p = 0.029) at day 169, respectively; S5 Table).

### Skin-based intrinsic gene expression subset assignment

Gene expression profiling was performed for all 45 skin biopsy samples corresponding to 19 unique patients. These included lesional baseline and follow-up biopsies for 12 patients (24 samples) and non-lesional baseline biopsies for 11 of these 12 patients (13 samples, including technical replicates) as well a follow-up non-lesional biopsy for one of these 12 patients (1 sample). The remaining seven samples corresponded to baseline non-lesional skin biopsies from a different set of seven patients.

**Fig 4:**
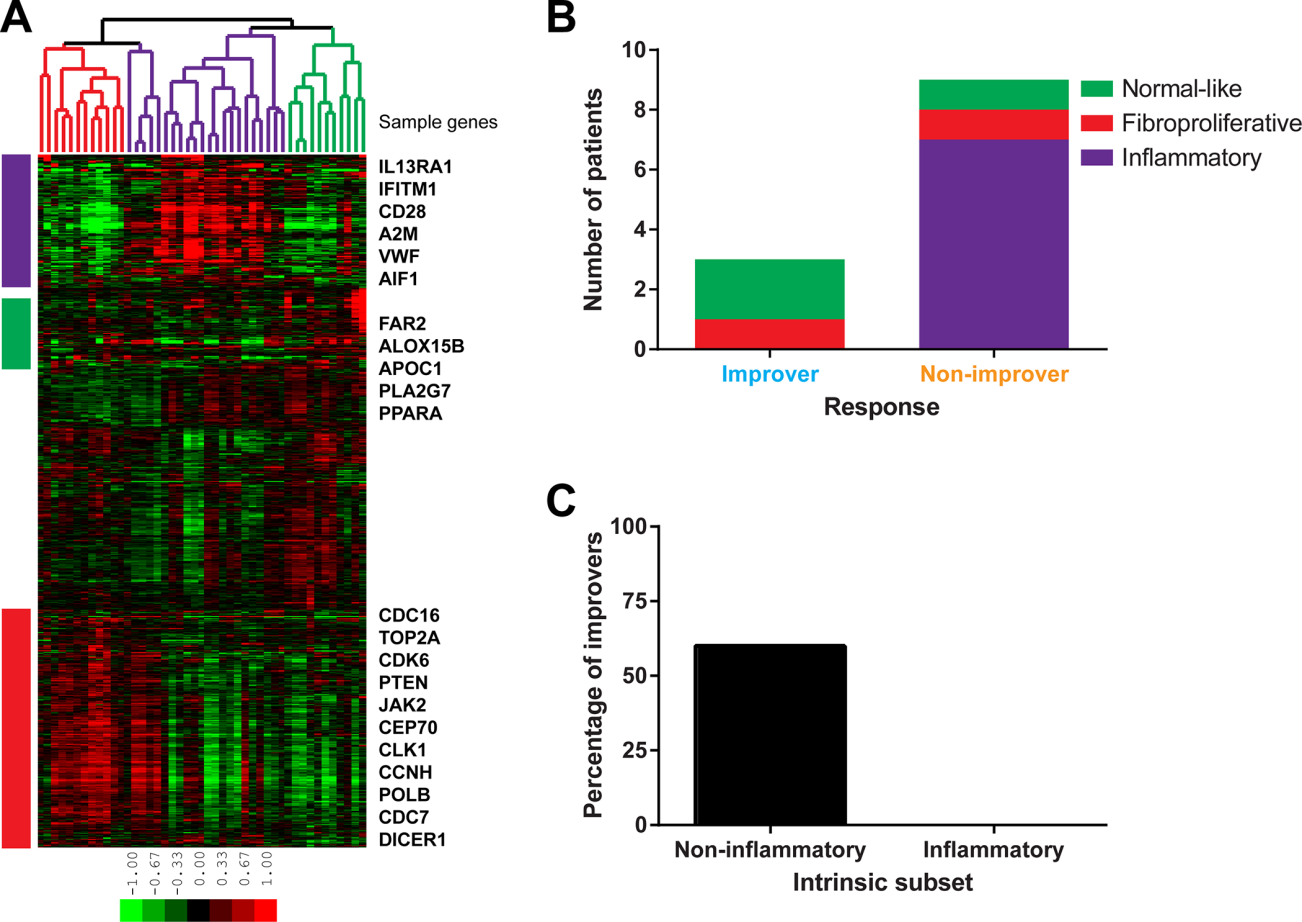
Intrinsic gene expression subset assignment and response status. (A) Intrinsic gene analysis. 3,207 probes (2,532 unique genes) at FDR<1.1% were used to organize samples into three gene expression-based clusters (intrinsic subsets). Sample color legend: red–fibroproliferative, purple–inflammatory, green–normal-like. (B) Distribution of patients according to their baseline intrinsic subsets and their response status. (C) Comparison between inflammatory and non-inflammatory (fibroproliferative and normal-like) patients in terms of the percentage of improvers.

First, each skin biopsy was assigned to an intrinsic gene expression subset. For this purpose, we identified 3,207 probes (2,532 unique genes) at false discovery rate (FDR)<1.1% using the intrinsic gene expression algorithm that finds genes with the most similar expression between samples from the same patient but with the largest differences between samples from different patients [35,44,45]. Using these genes for hierarchical sample clustering, three previously described intrinsic subsets (fibroproliferative, inflammatory and normal-like) [35,44–46] were observed (Fig 4A). We used g:Profiler [47] to identify functional terms significantly enriched in gene signatures associated with intrinsic subsets (p≤0.05, corrected for multiple testing via default g:Profiler method). Genes with increased expression in samples from fibroproliferative subset (Fig 4A, red bar) were enriched in cell cycle-related terms such as *mitotic cell cycle*, *chromosome organization*, *cell division* and *microtubule cytoskeleton*. Genes associated with the inflammatory subset (Fig 4A, purple bar) showed enrichment in *immune system process*, *defense response* and *inflammatory response*. Genes that were upregulated in normal-like samples (Fig 4A, green bar) were involved in *lipid metabolic process*, *fatty acid metabolic process* and *lipid biosynthetic process*. The absence in this cohort of limited intrinsic subset was not surprising, since only subjects with dcSSc were included in this study. S8 Table contains lists of annotated probes associated with each intrinsic subset.

### Intrinsic subset assignment and clinical response

Next, we compared the intrinsic gene expression subsets between subjects classified as clinical improvers and non-improvers (S9 Table). Overall, 7/12 subjects with matched biopsies mapped to the inflammatory, 2/12 to the fibroproliferative, and 3/12 to the normal-like subset at baseline. Significantly, we found that all three clinical improvers were non-inflammatory, mapping to the normal-like (2/3) or fibroproliferative (1/3) intrinsic subsets whereas 7/9 non-improvers were found to map to the inflammatory intrinsic subset (p = 0.0455; Fisher’s exact test) (Fig 4B). Three of five subjects (60%) mapping to the non-inflammatory (i.e. normal-like and fibroproliferative) subsets were classified as improvers, whereas none of the seven subjects (0%) mapping to the inflammatory subset were classified as improvers (Fig 4C).

### Comparison of baseline gene expression between improvers and non-improvers

**Fig 5:**
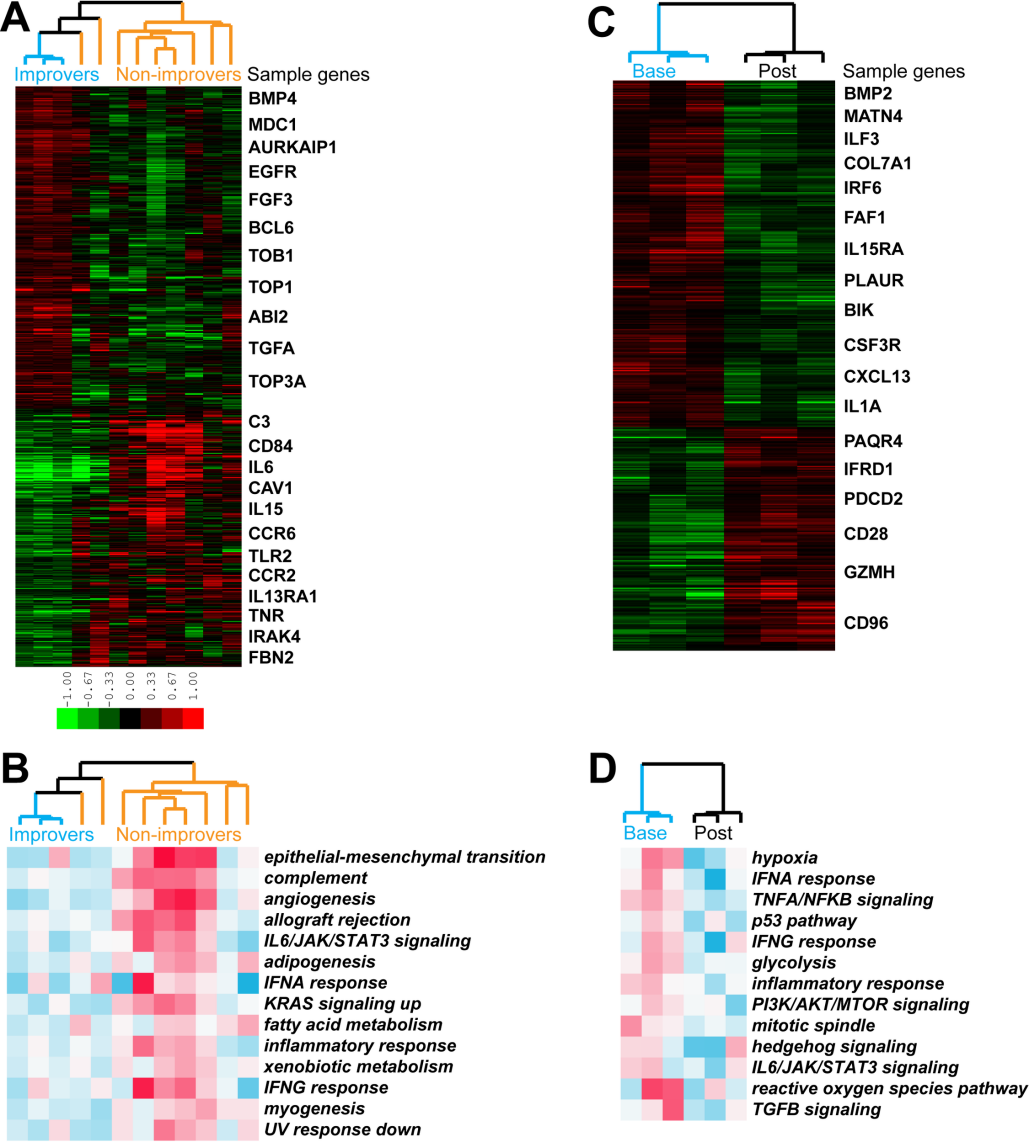
Baseline and improver gene expression and pathway enrichment analysis. (A) 1,062 genes were differentially expressed (p<0.05) between improvers and non-improvers at baseline. Sample color legend: blue–improvers, orange–non-improvers. (B) 14 pathways showing differential expression (FDR<10%) at baseline. (C) 454 genes displaying differential expression (p<0.05) between baseline and post-treatment in improvers. Sample color legend: blue–baseline samples, black–post-treatment samples. (D) 13 pathways were differentially expressed (FDR<10%) in improvers.

We sought to compare baseline gene expression between improvers and non-improvers. This analysis identified 1,062 genes that showed differential expression between the two groups (p<0.05; unpaired t-test) (Fig 5A). Genes showing increased baseline expression in improvers were enriched in *cell cycle*, *intracellular signal transduction* and *MAPK cascade*. The genes showing elevated baseline expression in non-improvers were associated with *defense response*, *inflammatory response* and *leukocyte migration*. There were 14 pathways that were differentially expressed (FDR<10%) between improvers and non-improvers at baseline. Non-improvers showed increased expression of inflammatory pathways, including *complement*, *allograft rejection* and *IL6/JAK/STAT3 signaling*, among others (Fig 5B).

### Changes in gene expression in improvers

Further analysis identified 454 genes that showed change from baseline to post-treatment skin biopsies in improvers (p<0.05; paired t-test) (Fig 5C). These genes showed higher baseline expression in improvers compared to non-improvers, and decreased post-treatment only in improvers (p<0.0001 for both comparisons) whereas their expression showed no change in non-improvers (p = 0.1576) (S3 Fig). Thirteen pathways (FDR<10%) were down-regulated post-treatment in improvers (Fig 5D) and included both inflammatory (e.g. *IFNA/IFNG response* and *TNFA/NFKB signaling*) and fibrotic gene sets (e.g. *PI3K/AKT/MTOR signaling* and *TGFB* signaling). No such change in pathways was observed in non-improvers.

### PFT results by clinical response

Lung functions (FVC and D_L_CO) remained stable in improvers during the course of the study (p = 0.75 for both PFTs). In marked contrast, a significant decrease in D_L_CO was seen in non-improvers (p = 0.0195; mean ± SEM -2.8 ± 1.0 mL/min/mmHg). Moreover, non-improvers also displayed a trend toward a decline in FVC at follow-up (p = 0.1289; mean ± SEM -0.3 ± 0.2 L).

### Quantitative HRCT, clinical response and intrinsic subsets

**Fig 6:**
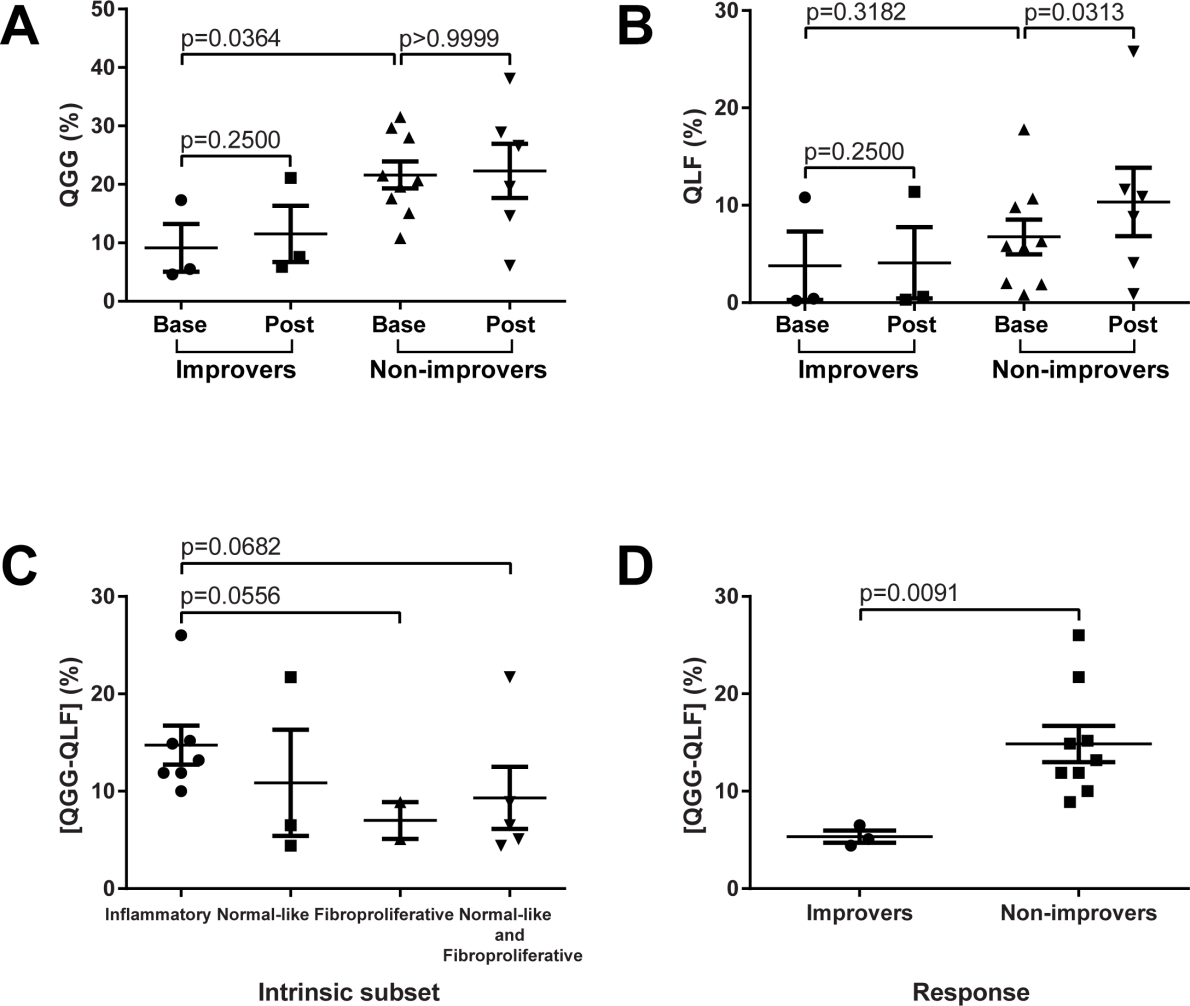
HRCT trends across improvers and non-improvers. (A) QGG trends. (B) QLF trends. (C) Baseline [QGG-QLF] marker vs. intrinsic subset. (D) Baseline [QGG-QLF] marker vs. response status.

Finally, we evaluated the radiologic parameters of lung fibrosis in improvers and non-improvers, as defined above. This analysis showed that non-improvers had significantly higher QGG scores at baseline, compared to improvers (p = 0.0364) (Fig 6A). QLF score in non-improvers increased significantly during therapy (p = 0.0313), while it remained stable in improvers (Fig 6B). Non-improvers also tended to have a higher QILD at baseline compared to improvers (p = 0.1212).

We next compared quantitative HRCT measures across intrinsic gene expression subsets (S10 Table). Specifically, we examined the relative differences in terms of the imaging marker of ground glass and fibrotic reticulation (measured as [QGG-QLF], large score indicating higher degree of inflammation relative to fibrosis in lung). At baseline, subjects mapping to the inflammatory subset had higher [QGG-QLF] scores (mean score 14.7%) compared to subjects mapping to fibroproliferative (p = 0.0556; mean score 7.0%), or combined normal-like and fibroproliferative intrinsic subsets (p = 0.0682; mean score 9.3%) (Fig 6C). Consistent with the fact that most non-improvers mapped to the inflammatory gene expression subset, baseline [QGG-QLF] scores were significantly higher in non-improvers compared to improvers (p = 0.0091; mean ± SEM 14.9 ± 1.9% for non-improvers vs. 5.3 ± 0.6% for improvers) (Fig 6D).

## Discussion

While recent studies in SSc have uncovered myriad potential therapeutic targets, disease-modifying treatment remains elusive. In light of remarkable disease heterogeneity that is the hallmark of SSc, and the multitude and diversity of molecular drivers linked to pathogenesis, effective interventions will require selective targeting of individual pathways in a “precision medicine” approach [6,48]. Multiple tyrosine kinases associated with TGF-β and PDGF signaling have been implicated in skin and lung fibrosis, and are potential targets for therapy [49]. We therefore performed an open-label clinical trial of dasatinib, a TKI that targets *c-Abl*, the PDGF receptor and Src tyrosine kinases [50]. While dasatinib appeared to be well-tolerated in this SSc cohort of patients with early-stage ILD, no significant changes in MRSS or PFTs were observed. However, the pattern of changes on quantitative HRCT suggested a probable therapeutic response in some subjects, since the 65% showing stability of lung fibrosis during the treatment period is significantly higher than in a placebo-treated historical cohort, in which only 23% of subjects showed no progression [42]. Adverse events in dasatinib-treated SSc patients were comparable to those seen in CML patients [51].

The extent of radiologic lung involvement at baseline is a strong predictor of both subsequent disease progression and mortality in patients with SSc [52]. In addition, radiologic change in SSc-associated lung fibrosis has been shown to correlate with treatment efficacy [33,42]. We had developed an innovative robust imaging approach for quantifying radiologic parameters of ILD in SSc. An advantage of quantitative HRCT evaluation over conventional visual assessment is its ability to provide a linear scale for determining absolute change [31,42]. In addition, it allows for accurate regional (lobar) measures of disease extent, and change over time (Fig 3). As observed in this study, the most severely involved lung lobes have moderate-to-severe disease, and change over time is best observed using this metric over whole lung measures. In the present study, we found that the QLF score remained unchanged during the course of dasatinib treatment in most patients, while the QILD scores increased in approximately two-thirds of patients, and QGG score increased at follow-up. Of note, non-improvers had significantly higher baseline QGG scores compared to improvers. Additionally, the QLF scores remained stable during the course of dasatinib treatment in improvers, while in non-improvers these scores significantly worsened. This might suggest that the presence of a ground glass radiological pattern in patients with SSc-ILD is indicative of active disease progressing to fibrosis; or that TKI therapy may control fibrotic, but not inflammatory, component of the disease.

Serum levels of SP-D, KL-6 [53] and APRIL have all been previously shown to be elevated in patients with SSc-ILD [54]. While we found levels of KL-6 and SP-D to be elevated in our cohort of SSc-ILD patients, they exhibited marked variability and showed poor correlation with MRSS or with improver status. In contrast, elevated levels of KL-6 and SP-D were significantly associated with HRCT QLF scores. Adiponectin, an adipocyte-derived circulating protein with potential utility as a biomarker [55], was shown to be reduced in patients with SSc, and to have an inverse correlation with MRSS [43]. In the present study, we found that adiponectin levels during the course of this trial showed a significant rise. Interestingly, adiponectin expression is negatively regulated by tyrosine kinases [56], which might provide an explanation for its up-regulation following treatment with dasatinib, and deserves further investigation.

Skin biopsy-based gene expression analyses revealed fundamental differences between patients classified as improvers and non-improvers. Each of the clinical improvers mapped to non-inflammatory intrinsic gene expression subsets, whereas most non-improvers were assigned to the inflammatory intrinsic subset. Improvers showed increased expression of cell cycle genes while non-improvers were characterized by up-regulated expression of immune and defense response genes. These observations indicate that baseline gene expression analysis of skin biopsies, and in particular molecular intrinsic subset assignment, represents a valuable approach for matching SSc patients to appropriate therapies. In particular, the present results suggest that dasatinib is more likely to benefit SSc-ILD patients mapping to the non-inflammatory intrinsic subsets than patients from the inflammatory subset. This is a significant and novel finding, since prior examples of subset-specific responses in SSc have identified patients mapping to the inflammatory subset as more likely to respond to immunomodulatory therapies such as mycophenolate mofetil [35] or abatacept [36].

In addition to the baseline gene expression differences, we also identified genes and pathways that were differentially expressed between baseline and post-treatment biopsies in improvers. Pathways that decreased in improvers represented both inflammatory and fibrotic signaling and, importantly, included genes upregulated in response to TGF-β. There were no pathways that changed in non-improvers suggesting a targeted modulation by dasatinib of inflammation and fibrosis in clinical improvers.

We recognize certain limitations of the present study. These include the absence of healthy controls, and the absence of a placebo-treated comparator group. In view of these considerations, gene expression differences between improvers and non-improvers cannot be attributed solely to the treatment effect, and could conceivably be explained by natural history of disease. Similarly, the relatively small sample sizes hampered identification of robust differentially expressed gene signatures, although we complemented this by performing differential pathway analysis on a whole genome level and correcting for multiple hypothesis testing. It should also be noted that since the primary objectives of this study were safety and pharmacokinetics of the dasatinib treatment, skin biopsies were available only for a subset of patients. This might potentially limit the extent to which findings from the present gene expression analyses are representative of a larger patient population.

In a recent clinical trial of nilotinib, another TKI, in patients with dcSSc [57], we reported that patients mapping to the inflammatory subset showed improvement on nilotinib. These results seem to contrast with the current findings, where none of the patients from the inflammatory subset responded to dasatinib. This difference is arguably a reflection of the distinct kinase inhibitory spectra of dasatinib and nilotinib [58]. Additionally, time points and clinical response criteria differed between these two studies making it problematic to directly compare the results. For example, in the nilotinib study, gene expression was analyzed at baseline, 6 and 12 months while in the dasatinib study, it was measured only at baseline and 6 months. Moreover, clinical endpoints (change in MRSS) were determined at 12 months for nilotinib and at 6 months for dasatinib [57]. Despite inconsistency in baseline subset assignment, clinical improvers in both trials showed decreased expression of inflammatory and fibrotic pathways post-treatment, which was not observed in non-improvers.

An important finding from this trial relates to the PK properties of dasatinib in SSc. Our PK studies indicated that the C_max_ and AUC were approximately 60% and 65% lower in patients with SSc compared to healthy subjects receiving dasatinib [27]. Reduced exposure to dasatinib in SSc patients may be due to concomitant use of proton pump inhibitors (PPIs). It was shown previously that co-medication with antacids and/or PPIs can reduce dasatinib exposure due to its pH-dependent absorption [51]. Since the solubility of dasatinib decreases as pH increases, antacids and PPIs might reduce the absorption of dasatinib by increasing gastric pH [51]. Nineteen of 21 subjects in the present study who underwent PK evaluation were receiving therapy with PPIs, which likely contributed to the substantial decrease in dasatinib exposure.

## Conclusions

In this open-label trial, dasatinib appeared to be well-tolerated in subjects with early-stage SSc-ILD. While no significant changes in MRSS or PFT were observed at day 169, the number of subjects showing stability of radiological QLF score was higher than had been previously observed in a historical placebo-treated SSc cohort [42]. Subjects classified clinically as non-improvers were more likely to show evidence of disease progression on quantitative HRCT. Moreover, patients mapping to the inflammatory gene expression subset were less likely to show improvement during dasatinib treatment. The changes in skin gene expression seen in the improvers correlated with the QLF. These results are unlikely to represent spontaneous improvement, which is only infrequently reported in the absence of effective therapy [35,45]. Taken together, the present observations provide support for the utility of QLF and QGG evaluation of chest HRCT, and analyses of gene expression from baseline skin biopsies, as precision medicine tools for matching SSc patients with optimal therapies.

## Supporting information

S1 Checklist CONSORT checklist for the study. (DOC)

S1 File Complete study protocol. (PDF)

S1 Table Inclusion and exclusion criteria for the study. (DOCX)

S2 Table Summary of adverse events experienced by ≥3 patients. (DOCX)

S3 Table Summary of serious adverse events. (DOCX)

S4 Table Correlation between baseline quantitative HRCT scoring, PFT results, serum markers and MRSS. (DOCX)

S5 Table Correlation between change in quantitative HRCT, PFT results, serum markers and MRSS. (DOCX)

S6 Table Correlation between adiponectin levels, serum biomarkers and PFT at baseline. (DOCX)

S7 Table Correlation between adiponectin levels, serum biomarkers and PFT at follow-up (change (Δ) from baseline at 6 mo). (DOCX)

S8 Table Lists of probes (with their annotations) corresponding to three intrinsic subsets from Fig 3. Probe IDs in first column are color-coded according to the subset with which they are associated: inflammatory–purple, normal-like–green, fibroproliferative–red.
(XLSX)

S9 Table Skin scores, response status and baseline intrinsic subset information for 12 SSc-ILD patients with serial skin biopsies. (DOCX)

S10 Table Quantitative HRCT and intrinsic subsets. (DOCX)

S1 Fig Quantitative and semi-quantitative (visual) HRCT assessment and its correlation with PFT at baseline and changes at 6 month follow-up or earlier end of study visit. (A) Plot of FVC (% predicted) and QLF score at the most severe lobe. (B) Plot of changes in FVC and QLF score at the most severe lobe at baseline. (C) Plot of FVC and QLF score in whole lung at baseline. (D) Plot of changes in FVC and QLF score in whole lung. (E) Box-whisker plot of FVC by visually assessed whole lung fibrotic reticulation at baseline. The range of visual assessment is as follows: 0% (none), 1–5%, 6–25%, 26–50%, 51–75%, and >75%. (F) Box-whisker plot of changes in FVC by the changes of the visual assessment in fibrotic reticular pattern (better, same, worse) in whole lung; (G) Box-whisker plot of QLF score by the visual assessment of fibrotic reticulation at the most severe lobe at baseline. (H) Box-whisker plot of changes in QLF by the visual assessment of fibrotic reticulation at the most severe lobe. (I) Box-whisker plot of QLF score by the visual assessment of fibrotic reticulation in whole lung at baseline. (J) Box-whisker plot of changes in QLF by the changes of the visual assessment in fibrotic reticular pattern (better, same, worse) in whole lung.
(PDF)

S2 Fig **Change in levels of high molecular weight (HMW) adiponectin (A), total adiponectin (B), and ratio of HMW/total adiponectin (C).** Data represent mean of duplicates for each patient at day 1 and 169 (n = 25).
(PDF)

S3 Fig Trends of improver gene signature. Only the centroid for genes down-regulated post-treatment in improvers is shown; the centroid for genes upregulated post-treatment represents its mirror image. See Statistical analysis section for the centroid explanation. P-values are for Tukey’s multiple comparisons test following ordinary one-way analysis of variance (ANOVA). Data are displayed as mean ± SEM scatter plot.
(PDF)
