# Supplementary material for: Novel lung imaging biomarkers and skin gene expression subsetting in dasatinib treatment of systemic sclerosis-associated interstitial lung disease
Source: PLoS One. 2017 Nov 9;12(11):e0187580. doi: 10.1371/journal.pone.0187580 (PMC5679625; doi:10.1371/journal.pone.0187580)
Supplement: S1 File — (PDF) [file pone.0187580.s002.pdf]

Protocol Number: CA180267
IND Number: IND 103,319
EUDRACT Number N/A
Date: 30-Jul-2008
Revised Date 11-Sep-2009

Clinical Protocol CA180267

An Open Label Trial to Evaluate the Safety of Dasatinib in the Treatment of Scleroderma Pulmonary Interstitial Fibrosis

Revised Protocol Number: 03

Incorporates amendment(s) 04

Study Director / Central Medical Monitor
Ramesh Pappu, MD
Mail Stop J13-05
Route 206 & Province Line RD
Princeton, NJ 08543
Telephone (office): 609-252-4654
Fax: 609-252-7811

Protocol Manager
Roy E Helfrick, MA
Mail Stop J44-04
Route 206 & Province Line RD
Princeton, NJ 08543
Telephone (office): 609-252-4410
Fax: 609-252-6806

24-hr Emergency Telephone Number
USA: 1-866-470-2267
International: +1-908-904-5520

Bristol-Myers Squibb Research and Development
Route 206 & Province Line Road
Lawrenceville, NJ 08543

This protocol contains information that is confidential and proprietary to Bristol-Myers Squibb

Replace all previous version(s) of the protocol with this revised protocol and please provide a copy of this revised protocol to all study personnel under your supervision, and archive the previous versions.

DOCUMENT HISTORY

Document	Date of Issue	Summary of Change
Revised Protocol 03	11-Sep-2009	Incorporates Amendment 04
Amendment 04	11-Sep-2009	1) Update the Time and Event Table to remove the skin biopsies at Days 365 and 533.<br>2) To clarify pregnancy testing requirements between scheduled visits.<br>3) To update the process for submission of Serious Adverse Events (SAEs) to BMS.<br>4) Correct discrepancies from the last amendment.
Revised Protocol 02	10-Jun-2009	Incorporates Amendment 03
Amendment 03	10-Jun-2009	1) Change contact information for central medical monitor.<br>2) Update the protocol to reflect an additional 18 months of study duration.<br>3) Update the time and events schedule to outline the procedures to be followed for the additional 18 months of the study.<br>4) Correct discrepancies from the last amendment.
Revised Protocol 01	04-Nov-2008	Incorporates Amendment 02
Amendment 02	04-Nov-2008	Clarifies certain inclusion/exclusion criteria and safety reporting.
Original Protocol	30-Jul-2008	Not applicable

Approved v7.0 930029550 5.0

SYNOPSIS

Clinical Protocol CA180267

Title of Study: An Open Label Trial to Evaluate the Safety of Dasatinib in the Treatment of Scleroderma Pulmonary Interstitial Fibrosis

Estimated Number of Study Centers and Countries/Regions: 15/ US

Study Phase: II

Research Hypothesis: Dasatinib will be safe in the treatment of scleroderma-associated pulmonary fibrosis.

Primary Objective: The primary objective of this study is to evaluate the safety of dasatinib in a population of subjects with scleroderma pulmonary fibrosis.

Study Design: Open Label

Duration of Study: This is a 30 subject, United States only, open label, safety study.

The duration of dasatinib dosing in this study will be six months to the primary endpoint. An additional 18 months of dosing will be provided unless the study is terminated for safety reasons.

Number of Subjects per Group: 30

Study Population: Patients to be recruited will be at least 18 years of age with established diffuse cutaneous scleroderma with a modified Rodnan skin score of at least 15 and evidence of fibrosing alveolitis as defined below on pulmonary function AND high resolution computed tomography (HRCT) scan of the chest.

Investigational Product(s), Dose and Mode of Administration, Duration of Treatment with Investigational Product(s): Dasatinib 100 mg (supplied in 50mg and 20mg tablets) once a day orally for 24 months.

Study Assessments and Primary Endpoints: Subject assessments will include: Physical exams, High resolution CT scans, Echocardiography, Chest X-rays, Pulmonary function testing, and laboratory assessments. See section below for a endpoint definitions.

Statistical Methods:

Sample Size Determination

The study will include 30 subjects, a sample size that provides a 45% probability of observing at least one death, a 79% probability of observing at least one incidence of pleural effusion and a 96 % probability of observing one occurrence of pericardial effusion. Exact 95% two-sided binomial confidence interval (CI) around the occurrence of any one of the aforementioned adverse events is (0.0008, 0.1722); for the occurrence of two such events in this study population the CI is (0.0082, 0.2207).

Populations for Analyses

There are 3 patient populations for purposes of statistical analyses in this study:

- enrolled subjects: All subjects who signed an informed consent
- treated subjects: All subjects who received at least one dose of dasatinib and will be use for safety analyses
- response-evaluable subjects: All treated subjects who have at least one post-baseline assessment of FVC. The all response-evaluable subjects will be used for efficacy analyses.

Approved v7.0 930029550 5.0

Endpoint Definitions

The primary safety endpoints include the following:

- Incidence of drug-related death
- Incidence of discontinuation related to significant drug-related pericardial effusion
- Incidence of discontinuation related to significant drug-related pleural effusion
- Incidence of discontinuation related to significant drug-related peripheral edema
- Incidence of discontinuation related to significant drug-related bone marrow suppression
- Incidence of discontinuation related to significant worsening of underlying scleroderma
- Incidence of discontinuation related to other unforeseen toxicities of dasatinib in this population.

Planned Statistical Analysis

The “all treated subjects” population will be used for safety analyses. Each primary endpoint incidence rate is calculated as the proportion of subjects with a discontinuation reason due to the primary endpoint divided by the number of “all treated subjects”.

The frequency and percentage of each primary safety endpoints, along with an exact 95% two-sided binomial confidence interval, will be provided without adjustment for multiple comparisons.

The frequency of occurrence of adverse events (AEs) and laboratory abnormalities will be provided. Tabulations of AEs considered at least possibly-related to study drug and of AEs regardless of relationship will be provided separately.

Exploratory analyses will be performed of the relationship between the incidence of drug-related adverse events and extent of prior treatment, pre-study AEs, study drug dose and treatment duration.

The “all response-evaluable subjects” population will be used for efficacy analyses. For continuous measurements, such as FVC, mean change from baseline to each scheduled study visit and its standard error will be estimated. The mean change from baseline values, along with individual change from baseline values at each study visit, will be presented in a plot. Individual best response values, which are defined as the largest percent change from baseline to any study visit, will be presented.

For categorical measurement, such as scleroderma skin severity score, frequency and percentage of each category will be presented.

Approved v7.0 930029550 5.0

TABLE OF CONTENTS

TITLE PAGE 1

DOCUMENT HISTORY 2

SYNOPSIS 3

TABLE OF CONTENTS 5

1 INTRODUCTION AND STUDY RATIONALE 11

1.1 Research Hypothesis 14

1.2 Investigational Product Development Rationale 14

1.2.1 Dosing Rationale 14

1.2.2 Experience in CML 17

1.2.3 Phase I Experience in Solid Tumors: CA180003 and CA180021 19

1.3 Summary of Results of Investigational Program 20

1.4 Study Rationale 22

1.5 Overall Risk/Benefit Assessment 22

2 STUDY OBJECTIVES 24

2.1 Primary Objective 24

2.2 Exploratory Objectives 25

3 ETHICAL CONSIDERATIONS 25

3.1 Good Clinical Practice 25

3.2 Institutional Review Board/Independent Ethics Committee 26

3.3 Informed Consent 26

Approved v7.0 930029550 5.0

4 INVESTIGATIONAL PLAN 27
4.1 Study Design and Duration. 27
4.2 Study Population 27
4.2.1 Inclusion Criteria 28
4.2.2 Exclusion Criteria 29
4.2.3 Discontinuation of Subjects from Treatment 31
4.3 Data Monitoring Committee 32
5 TREATMENTS. 32
5.1 Study Treatment 32
5.1.1 Investigational Product 32
5.1.2 Noninvestigational Product 33
5.1.3 Identification 33
5.1.4 Packaging and Labeling 33
5.1.5 Handling and Dispensing 33
5.2 Method of Assigning Subjects to a Treatment 35
5.3 Selection and Timing of Dose for Each Subject 35
5.3.1 Dose Modifications 35
5.4 Blinding/Unblinding. 36
5.5 Concomitant Treatments 36
5.5.1 Prohibited and/or Restricted Treatments 37
5.5.1.1 Medications that Prolong QTC Interval 37
5.5.2 Other Restrictions and Precautions 37
5.5.2.1 Medications that Inhibit Platelet Function and Anticoagulants 37
5.5.2.2 CYP3A4 Inhibitors. 38

Approved v7.0 930029550 5.0

5.5.2.3 CYP3A4 Inducers 38

5.5.2.4 CYP3A4 Substrates 38

5.5.2.5 Antacids 39

5.5.2.6 H2 Blockers/Proton Pump Inhibitors 39

5.6 Treatment Compliance 40

6 STUDY ASSESSMENTS AND PROCEDURES 40

6.1 Flow Chart/Time and Events Schedule 40

6.2 Study Materials 47

6.3 Safety Assessments 47

6.3.1 Laboratory Assessments to include: 48

6.3.2 Follow Up Visits 49

6.4 Efficacy Assessments 49

6.5 Pharmacokinetic Assessments 50

6.5.1 Pharmacokinetics: Blood Collection and Processing 50

6.5.2 Pharmacokinetic Sample Analysis 50

6.6 Pharmacodynamics Assessments 51

6.6.1 Plasma/Serum Soluble Mediators 51

6.6.2 Exploratory RNA Profiling 51

6.7 Pharmacogenomic/Pharmacogenetic Assessments . . 52

6.8 Outcomes Research Assessments 52

6.9 Other Assessments 52

7 ADVERSE EVENTS 52

7.1 Definitions 52

7.1.1 Serious Adverse Events 52

7.1.2 Nonserious Adverse Events 53

Approved v7.0 930029550 5.0

7.2 Assignment of Adverse Event Intensity and Relationship to Investigational Product 54
7.3 Collection and Reporting 54
7.3.1 Serious Adverse Events 55
7.3.2 Handling of Expedited Safety Reports 56
7.3.3 Nonserious Adverse Events 57
7.4 Laboratory Test Abnormalities 57
7.5 Overdose 58
7.6 Pregnancy 58
7.6.1 Requirements for Pregnancy Testing 59
7.6.2 Reporting of Pregnancy 59
7.7 Other Safety Considerations 60
8 STATISTICAL CONSIDERATIONS. 60
8.1 Sample Size Determination 60
8.2 Populations for Analyses 61
8.3 Endpoint Definitions 61
8.4 Analyses 61
8.4.1 Demographics and Baseline Characteristics 62
8.4.2 Safety Analyses 62
8.4.3 Efficacy Analyses 62
8.4.4 Pharmacokinetic Analyses 63
8.4.5 Pharmacodynamic Analyses. 63
8.4.6 Pharmacogenomic Analyses 63
8.4.7 Outcomes Research Analyses 63
8.4.8 Other Analyses 63

Approved v7.0 930029550 5.0

8.5 Interim Analyses 63

9 ADMINISTRATIVE SECTION 63

9.1 Compliance 63

9.1.1 Compliance with the Protocol and Protocol Revisions 63

9.1.2 Monitoring 64

9.1.3 Investigational Site Training 65

9.2 Records Retention 65

9.2.1 Case Report Forms 66

9.2.2 Investigational Product Records 67

9.3 Return and Destruction of Investigational Product 67

9.3.1 Return of Investigational Product 67

9.3.2 Destruction of Investigational Product 68

9.4 Publications 68

10 GLOSSARY OF TERMS 69

11 LIST OF ABBREVIATIONS 70

12 REFERENCES 72

APPENDIX 1 ADDITIONAL ETHICAL CONSIDERATIONS . . 76

APPENDIX 2 CRITERIA FOR THE CLASSIFICATION OF SYSTEMIC SCLEROSIS (SCLERODERMA) 80

APPENDIX 3 SKIN SCLEROSIS SCORE 82

APPENDIX 4 NEW YORK HEART ASSOCIATION (NYHA) CLASSIFICATION 84

Approved v7.0 930029550 5.0

APPENDIX 5 COMMON CYP3A4 SUBSTRATES, INHIBITORS AND INDUCERS (NOT ALL INCLUSIVE) . . 85

APPENDIX 6 LABORATORY GUIDELINES: PATIENT STUDIES (REVISED AUGUST 13, 1999) 89

Approved v7.0 930029550 5.0

1 INTRODUCTION AND STUDY RATIONALE

Scleroderma (systemic sclerosis) is a multisystem auto-immune and fibrotic disorder characterized by damage of small blood vessels, deposition of excess intracellular matrix and fibrosis of a variety of organ systems[1] and immune activation.[2] Scleroderma causes thickening of skin, progressive and often severe dysfunction of a number of internal organs. Raynaud phenomenon and non-specific complaints like fatigue and musculoskeletal pain are usually the first manifestation of scleroderma, often occurring in the absence of any other clinical features. Damage of the kidneys, lungs, gastrointestinal tract, heart and musculoskeletal systems is common.[1]

Previously the major cause of death in scleroderma was “renal crisis” (oliguric renal failure, often with malignant hypertension), but the introduction of angiotensin converting enzyme (ACE) inhibitors has made this much less common (although ACE inhibitors have not entirely eliminated this feature and its fatal outcomes in scleroderma).[1] Currently, the most common cause of death in scleroderma is pulmonary disease, interstitial fibrosis and/or vasculopathy, leading to pulmonary hypertension, processes which may occur and progress independently of each other.[1, 3, 4]

Scleroderma associated pulmonary fibrosis represents a compelling unmet medical need; improvement in pulmonary function and reversal, even stabilization, of fibrosis would represent a major step forward in the management of scleroderma. Cyclophosphamide has been a mainstay of therapy for severe, potentially fatal rheumatologic diseases for over three decades, since its efficacy was identified in vasculitis and lupus nephritis. Thus, exploration of its use in scleroderma was logical. However, recent studies have demonstrated that cyclophosphamide is of only marginal utility in the treatment of scleroderma-related pulmonary fibrosis;[5, 6, 7, 8] nonetheless, in the absence of any other effective therapy, cyclophosphamide has become “standard of care” for this manifestation of scleroderma.[9] A recent study of cyclophosphamide followed by azathioprine was unsuccessful.[10] The manifold toxicities of cyclophosphamide can be severe, leading investigators to seek alternative, less toxic therapies for potentially fatal rheumatologic disorders, including scleroderma, where mycophenolate mofetil and imatinib are currently or soon to be evaluated.

Approved v7.0 930029550 5.0

The cause of scleroderma remains unclear. A number of auto-antibodies, most notably antibodies against topoisomerase I (topoisomerases are a group of enzymes that allow the DNA double-helix to unwind and may nick one of the DNA strands), are produced, although their role in the immunopathogenesis of scleroderma is as yet unclear;[1,4, 11] (anti topoisomerase I antibodies, were previously known as anti-Scl70 antibodies- a scleroderma antigen of 70 kDa mass). T-cell activation by unknown antigen(s) is also prominent in scleroderma.[4, 12] Excessive accumulation of extracellular matrix components in skin and involved organs ultimately dominates the histopathological picture.[1] The resulting fibrosis disrupts the function of involved organs. Although our understanding of the mechanisms by which fibroblast activation in scleroderma is incomplete, there is compelling evidence that both TGFβ (transforming growth factorβ) and PDGF (platelet-derived growth factor) play important roles. TGFβ and PDGF potently stimulate synthesis of extracellular matrix by fibroblasts; inhibition of TGFβ or PDGF signaling reduces fibrosis in scleroderma experimental models and overexpression of TGFβ in fibroblasts induces a scleroderma-like disease, with tissue fibrosis. [4, 13, 14, 15, 16] Based on these predictions the tyrosine kinase inhibitor imatinib has been proposed by others as a rational option in the treatment of rheumatic diseases, including scleroderma.[17, 18] Dasatinib has a broader spectrum of kinase targets than does imatinib, suggesting that it might have more potential for efficacy, especially due to the effects of dasatinib on immune function not shared by imatinib.

Dasatinib provides a unique spectrum of kinase inhibition that can target what are likely to be the two main pathophysiologic processes underlying scleroderma, fibrosis and autoimmune reactions. Fibrosis in scleroderma is believed to be induced by the action of TGFβ and PDGF, with additional involvement by DDR1 (Discoidin Domain Receptor 1). Dasatinib potently inhibits the kinases required for signal transduction by these agents. The Abl kinase is activated by TGFβ and regulates extracellular matrix production and fibroblast proliferation. Dasatinib potently inhibits Abl; indeed the bcr-Abl fusion protein is the primary target in CML. The Src-family kinases Src and Fyn are also activated by TGFβ, and dasatinib inhibits these kinases with similar potency, displaying Ki values in the range of 16 - 50 pM. The Src family kinases have been shown to be required for TGFβ induced collagen type 1 accumulation, cellular proliferation, and myofibroblast differentiation. Consistent with these mechanisms, dasatinib has been reported to potently

Approved v7.0 930029550 5.0

inhibit TGFβ-induced production of collagen 1a1, collagen 1a2, and fibronectin by cultured fibroblasts from SSc patients, displaying IC50 values in the range of 0.005 - 0.002 ug/mL [16]. The PDGF receptor is a receptor tyrosine kinase, and this kinase activity is essential for the action of PDGF. Dasatinib inhibits PDGF receptor tyrosine kinase activity with an IC50 of 28 nM. DDR1 is a receptor tyrosine kinase activated by collagen, and dasatinib inhibits DDR1 kinase activity with an IC50 of 7 nM. DDR1 is elevated in the lungs of patients with pulmonary fibrosis. DDR1 knockout mice are resistant to bleomycin induced lung fibrosis, displaying reduced TGFβ expression, collagen deposition, chemokine expression and inflammatory cell infiltration. Consistent with these mechanisms, dasatinib has been found to be highly efficacious in the bleomycin model of dermal fibrosis, reducing dermal thickness, myofibroblast number, and collagen content[16].

The potent inhibition of Src-family kinases, including Lck, and Fyn as well as Src, provides the mechanism by which dasatinib can inhibit T-cell, monocyte, and mast cell responses. Dasatinib inhibits T-cell activation and cytokine production, activities not inhibited by the other commercially available kinase inhibitors;[19, 20, 21, 22] T-cell activation is prominent in scleroderma[4, 12] and autoimmune reactivity is prominent.[2] Dasatinib also inhibits mast cell proliferation and activation;[23] mast cells are found in sclerodermatous lesions and are thought to be involved in the pathogenesis of fibrosis, possibly by release of pro-fibrogenic cytokines.[1] Finally, dasatinib also inhibits monocyte production of inflammatory cytokines, a potent source of many mediators of fibrosis.[24] The broad spectrum of kinase inhibition by dasatinib makes it a logical choice for treatment of scleroderma, by dint of its unique broad target spectrum that inhibits the function of a broad array of immune cells implicated in the pathogenesis of scleroderma,

Dasatinib is currently approved for use in Philadelphia chromosome-positive chronic myeloid leukemia resistant or intolerant to prior therapy including imatinib; dasatinib's use for the treatment of solid tumors is currently under investigation. Especially at the 70 mg BID dose used in studies of CML and solid tumors, dasatinib was associated with serositis (pleural effusions, pericardial effusions) and peripheral edema, as well as bone marrow suppression (in CML, far less so in solid tumors). This toxicity led to evaluation and subsequent approval of a 100 mg PO QD dosing regimen in CML, which is being

Approved v7.0 930029550 5.0

evaluated in solid tumors, as well. In the current study we will use the 100 mg PO QD dasatinib dose.

1.1 Research Hypothesis

Dasatinib will be safe in the treatment of scleroderma-associated pulmonary fibrosis.

1.2 Investigational Product Development Rationale

Scleroderma is a potentially fatal multi-system autoimmune disorder, with prominent production of excess extracellular matrix, fibrosis and vascular obstruction in a number of organs. TGFβ and PDGF (both of which transmit their signals to the target cell via tyrosine kinases) have been implicated in the pathogenesis of scleroderma, especially the fibrotic abnormalities. Pulmonary disease (interstitial fibrosis and/or arterial hypertension) is the major cause of death in scleroderma, having surpassed renal disease. Interstitial fibrosis may be present early in the course of scleroderma and may be mild and self-limited, but in the one-third of scleroderma patients with significant pulmonary fibrosis, it can be rapidly progressive and fatal. Currently there is no established and safe therapeutic option for any aspect of scleroderma.

Signaling through tyrosine kinases is critical to transmission of potentially pathogenic signals from TGFβ and PDGF. The ability of tyrosine kinase inhibitors to suppress fibrosis in animal models of scleroderma and in the bone marrow of subjects treated for CML strongly suggests that these inhibitors may be useful in suppressing or reversing the systemic fibrosis seen in scleroderma. Dasatinib in particular has a very broad spectrum of kinase inhibition, affecting not only fibroblasts, but also T-cells, B-cells, monocytes and mast cells (all of which have been implicated in the immunopathogenesis of scleroderma), suggesting that it may be even more effective in this underserved population than the other kinase inhibitors with a more limited range of specificities.

1.2.1 Dosing Rationale

Although there is no human experience with dasatinib in scleroderma, there has been work utilizing a bleomycin-induced model (which includes dermal and pulmonary interstitial fibrosis), explants of human scleroderma skin biopsies and in vitro studies of

Approved v7.0 930029550 5.0

normal fibroblasts. Doses of dasatinib in mice approximating the characteristics of the human dose of 70 mg PO BID (Cmax and AUC) eventuate in significant changes in immune function and in bleomycin induced dermal fibrosis; the PK characteristics of murine doses of 6 and 20 mg/kg/day are replicated by doses of 29 and 96 mg/day in humans (hematologic and solid tumor patients and normal healthy controls), respectively (See Table 1.2.1). There is no difference between dasatinib PK in these three human populations. For purposes of dose selection the PK characteristics in the scleroderma population is assumed to be the same as those of the 3 human populations already studied.

The production of extracellular matrix protein, and the mRNA encoding these proteins, was suppressed in cultures of explanted human fibroblasts stimulated with TGFβ or PDGF by levels of dasatinib that correlate with plasma levels easily achievable with these murine and human doses. There being no prior human experience with dasatinib in scleroderma, we referred to the studies noted above to determine that a dose of 100 mg PO QD will provide PK characteristics, including sufficient Cmax, to suppress the kinases which drive fibrotic changes in scleroderma. Based on the dasatinib experience in malignancies, this approved dose is expected to be safe and well tolerated.

Table 1.2.1: Comparison of pharmacokinetics of dasatinib in BMS and Distler murine studies

Study	Dose (mg/kg)	Cmax (ng/mL)	AUC (ng.h/mL)	Cmin (ng/mL)	Tmax (h)	T1/2 (h)
BMS	5	51	220	nr	2	2.5
	15	160	580	nr	2	2.0
Distler	3 BID	31	128	1.6	Nr	nr
	10 BID	104	399	4.3	nr	nr
	6 QD	62	256	0.05	Nr	nr
	20 QD	208	798	0.14	nr	nr

Akhmetshina et al. demonstrated that dasatinib reduced the mRNA and protein levels of extracellular matrix proteins in scleroderma patient dermal fibroblasts stimulated with TGFβ or PDGF with an IC50 of 0.5 to 2.0 nM [25] (2 nM dasatinib approximates 1 ng/mL

Approved v7.0 930029550 5.0

in tissue culture medium). In the mouse bleomycin-induced dermal fibrosis model 3 mg/kg PO BID and 10 mg PO BID dosages suppressed dermal thickness by approximately 10% and 20%, respectively; these doses were chosen to mimic the 70 mg PO BID dosage approved for the treatment of CML (Joerg Distler, personal communication). Luo, et al. evaluated the pharmacokinetics of dasatinib in C57/BL6 mice and found that a dose of 1.25 mg/kg BID achieved lower peak (10 ng/mL) plasma concentrations than those achieved with 70 mg PO BID in humans (40 ng/mL) and far lower than the peak achieved after a 100 mg oral dose (70 ng/mL); the amount of time the plasma concentration remained above the EC90 was 4 and 5 hours respectively)[26, 29]. A dose of 2.5 mg/kg QD in mice (peak concentration of 40 ng/mL) likewise achieved a lower peak concentration than did 140 mg PO QD in humans (approximately 80 ng/mL); as measured by inhibition of the Bcr-Abl tyrosine kinase, the amount of time the plasma concentration remained above the EC90 was 6 and 8 hours, respectively).

Schade, et al. explored dasatinib's ability to inhibit T-cell activation and proliferation and found that the 10 mg BID dosage in mice approximates the Cmax of the 70 mg PO BID dose in humans; this dose profoundly inhibits murine mixed lymphocyte reaction, cytokine production, cell proliferation, signaling through the T-cell antigen receptor and in vivo T-cell responses.[19]

Weichsel, et al. utilized dasatinib to suppress antigen-specific T-cell effector functions and demonstrated that "clinically relevant doses" of 1 to 100 nM added to cell cultures for only 1 hour prior to stimulation were capable of suppressing a variety of functions: CD4+ cells (IC50 of 10 nM); CD8+ cell activation (IC50 of 16 nM); naive CD8+ cell activation (IC50 of 16 nM); naive CD4+ cell activation (IC50 of 12 nM); naive CD8+ cell proliferation (IC50 of 15 nM); naive CD4+ cell proliferation (IC50 of 9 nM); activation and proliferation of memory T-cells was slightly less sensitive to dasatinib, with IC50 ranging from 13 to 26 nM. Dasatinib (10 nM) completely suppressed production of TNF -α, γ--interferon and IL-2 by $\gamma\delta$ T-cells; IC50 was not determined in these experiments[27]. After a 100 mg oral dose of dasatinib, serum concentrations are over 10 ng/mL for approximately 6 hours.

In none of the experiments cited has there been any evidence that dasatinib induces apoptosis of T-cells.

Approved v7.0 930029550 5.0

Our intention is to achieve a persisting serum concentration to exceed the concentrations and IC50s noted in the studies (IC90 levels were not provided). The target is a serum dasatinib concentration of 20 to 25 nM to adequately suppress the relevant kinases. We believe the PK in normal healthy controls and in patients with leukemia and solid tumors will be representative of scleroderma patients, as well. The 100 mg daily dose of dasatinib in prior studies achieves a serum level of greater than 20 to 25 nM for over 4 hours. As noted in Section 5.5.2.6, optimal absorption of dasatinib requires an acid gastric pH. The widespread use of proton-pump inhibitors, H2 blockers and antacids in patients with scleroderma (as treatment of their nearly universal symptoms of gastroesophageal reflux) will likely decrease absorption of dasatinib, an effect that informs our decision to start the trial with the 100 mg PO QD dose. The pharmacokinetics of dasatinib in scleroderma will be explicitly studied in this trial, to assure that these patients achieve the desired therapeutic dasatinib serum level, regardless of concomitant medications.

In summary, doses of dasatinib in mice approximating the characteristics of human dose of 70 mg PO BID eventuate in significant changes in immune function and in bleomycin induced dermal fibrosis. The production of extracellular matrix protein and mRNA for these proteins was suppressed in cultures of explanted human fibroblasts stimulated with TGFβ or PDGF by levels of dasatinib that correlate with plasma levels easily achievable by these murine and human doses. The peak concentration achieved by the 100 mg PO QD dose is higher than that achieved with the 70 mg PO BID dosing regimen and it is anticipated that this will eventuate in greater kinase suppression.

1.2.2 Experience in CML

Although the toxicity experience with CML is not thought to be as relevant to scleroderma as is the array of toxicities in the solid tumor program (summarized below), it is in the CML program where the most thorough dose-ranging exploration took place, notably relevant to fluid retention. As illustrated in this table the incidences of three forms of fluid retention increased over time. These data were derived from a total of 165 patients treated at this dosed level.

Approved v7.0 930029550 5.0

Table 1.2.2A: Common Drug Related Non-Hematologic Toxicity Profile for Dasatinib Single Agent

Preferred Term (PT)	CML and Ph+ ALL (Any grade) N = 2182 (%)	Solid Tumor (Any grade) N = 164 (%)
Pleural effusion	535 (25%)	52 (31%)
Diarrhea	698 (32%)	75 (45.7%)
Headache	535 (25%)	52 (31.7%)
Rash	485 (22%)	44 (26.8%)
Nausea	480 (22%)	72 (43.9%)
Dyspnea	467 (21%)	48 (29.3%)
Fatigue	467 (21%)	60-(36.6%)
Edema	29 (1%)	6 (4 %)

At doses of 50 mg PO BID and 70 mg PO BID the rates of pleural effusion and other fluid retention are much higher than at the 100 mg PO QD at any time point. Thus, QD dosing appears to be safer than BID dosing, with 70 mg PO BID associated with somewhat more fluid retention than 50 mg PO BID.

Table 1.2.2B: Data at 50 mg PO BID (N = 167) Percent patients (% Grade 3 or 4)

**Duration of follow-up**			
	**8 months**	**1 year**	**2 years**
Pleural effusion	11.4 (1.8)	16.2 (3.0)	22.8 (3.6)
Congestive heart failure/cardiac dysfunction	1.2 (0.6)	1.2 (0.6)	1.2 (0.6)
Pericardial effusion	1.2 (0.6)	2.4 (0.6)	6.0 (2.4)
Pulmonary edema	0.6 (0)	0.6 (0)	1.2 (0.6)
Generalized edema	0.6 (0)	0	0
Superficial edema	16.3 (0)	18.6 (0)	23.4 (0)

Approved v7.0 930029550 5.0

Table 1.2.2C: Data at 70 mg PO BID N = 167 Percent patients (% Grade 3 or 4)

Duration of follow-up			
	**8 months**	**1 year**	**2 years**
Pleural effusion	18.0 (1.8)	20.4 (3.0)	25.1 (6.0)
Congestive heart failure/cardiac dysfunction	4.8 (3.6)	6.0 (4.2)	6.6 (4.2)
Pericardial effusion	1.2 (0.6)	2.4 (1.2)	2.4 (1.2)
Pulmonary edema	1.2 (0.6)	1.8 (0.6)	3.0 (0.6)
Generalized edema	0.6 (0)	0.6 (0)	0.6 (0)
Superficial edema	18.0 (0)	22.2 (0)	25.7 (0)

1.2.3 Phase I Experience in Solid Tumors: CA180003 and CA180021

Experience with solid tumors is considered to be more applicable than that with leukemia, so the current experience with solid tumors will be summarized. In study CA180003, dasatinib has been administered on a BID schedule to 42 subjects with refractory solid tumor. To date, doses up to 160 mg BID on a 5-day on/2-day off schedule have been administered. A dose of 120 mg BID continuous daily schedule is currently under investigation.

No severe clinical toxicity has been encountered. Gastrointestinal symptoms were reported in most subjects, fatigue was reported in 17 subjects (40%) and rash in 10 subjects (24%). Edema, lethargy and headache were uncommon, and appear to be dose-related. Grade 3 asymptomatic hypocalcemia was considered dose-limiting in one subject, Grade 2 rash was considered dose-limiting in two other subjects, and Grade 2 nausea and vomiting (with dysarthria, lightheadedness and lethargy in a 49 kg subject taking concurrent diazepam) was considered dose-limiting in one subject.

In study CA180021, dasatinib has been administered on a QD schedule to 24 subjects at doses up to 180 mg. Pleural effusions have been observed in three subjects at the 180 mg dose level (one with pneumonia and two with malignant effusion). A dose of 250 mg QD is currently under consideration. Hypocalcemia, GI symptoms and skin rash have been mild and infrequent.

Approved v7.0 930029550 5.0

To date, the safety profile in solid tumor subjects has been similar to that in CP CML subjects with the exception of severe myelosuppression, which has not been observed in solid tumor subjects and is considered related to efficacy against the leukemia as noted above, and severe bleeding which is secondary to thrombocytopenia in most instances.

Thus the dose of dasatinib chosen, 100 mg PO QD (approved for CML), has been shown to be safer than the prior approved dosage of 70 mg PO BID. Based on the human explant and murine data reported summarized above, the 100 mg PO QD dose should be sufficient to suppress fibroblast and immune cell function for much of the 24 hour cycle. The 100 mg PO QD dosage is successful in suppressing kinases involved in the pathogenesis of CML and related leukemias and has been effective in initial trials in solid tumors as well. At least one kinase serves as a distinct target in CML, ie Bcr-Abl, and this dosage of dasatinib is sufficient to suppress its activity. Dasatinib displays similar activity against the Src family kinases which play important roles in the pathogenetic mechanisms implicated in scleroderma, including the production of extracellular matrix components and fibrosis (fibroblasts and cytokine-producing cells), as well as in the activities of immune cells of the acquired (B and T-cells) and innate (monocytes, mast cells) immune system. The exact nature and extent of the contribution that each kinase target of dasatinib makes to the pathophysiology of scleroderma is not known, and may well vary among patients. However, the dosage of dasatinib proposed is expected to inhibit relevant responses in all of these cell types important in scleroderma pathophysiology. Thus, this dose represents a prudent choice for anticipated safety and tolerability, as well as efficacy.

1.3 Summary of Results of Investigational Program

There is no prior dasatinib investigational program in scleroderma. A widely used, albeit imperfect, animal of scleroderma is the bleomycin-induced fibrosis system. A recent paper describing the efficacy of the tyrosine kinase inhibitor imatinib in this model summarizes the effects of this class of compounds in scleroderma models[16]. Varga summarizes the current hypotheses for the pathogenesis of scleroderma and the potential role of tyrosine kinase inhibitors, and Paniagua goes further, suggesting a role for this class of compounds in many rheumatologic diseases.[28] A trial of imatinib in systemic sclerosis is about to begin; the imatinib trial does not target pulmonary fibrosis, but

Approved v7.0 930029550 5.0

focuses on skin and all other manifestations of scleroderma are assessed, as well. Thus, with current confidence in this class of agents and the broader specificities of kinases inhibited by dasatinib, the current trial is proposed.

In vitro studies have demonstrated that dasatanib successfully inhibits signaling through Bcr-Abl (the pathogenic fusion product in Philadelphia chromosome-positive chronic myeloid leukemia), Src and DDR1. Dasatinib inhibits a broader spectrum of protein kinases than do most other kinase inhibitors.[29] Of relevance to this disease, dasatinib also inhibits immune cells implicated in the pathogenesis of scleroderma. Dasatinib inhibits T-cell activation and cytokine production, activities not inhibited by the other commercially available kinase inhibitors.[19, 20, 21, 22] Dasatinib inhibits mast cell proliferation and activation;[23] mast cells are found in sclerodermatous lesions and are thought to be involved in the pathogenesis of fibrosis, possibly by release of pro-fibrogenic cytokines. Dasatinib inhibits monocyte production of inflammatory cytokines, a potent source of many mediators of fibrosis.[24] This broad range of cellular targets is due to the broader spectrum of kinases targeted by dasatinib. Inhibition of auto-immunity, mast cell and monocyte activation in addition to suppression of fibrosis makes dasatinib a very attractive therapeutic option for scleroderma.

The current trial is designed to evaluate the safety of dasatinib in this new patient population. Specific attention will be paid to toxicities encountered in the dasatanib oncology studies, including the prevalence and severity of pleural and pericardial effusions and peripheral edema; the prevalence and severity of bone marrow suppression; and toxicities unique to the scleroderma population. If adverse events should occur, the investigator is provided with a prescribed dosing decrease formula for defined thresholds of toxicity in order to continue to evaluate efficacy and the resolution of toxicity.

The dose of dasatinib chosen, 100 mg PO QD (approved for CML), has been shown to be safer than the prior approved dosage of 70 mg PO BID. The 100 mg PO QD dosage is successful in suppressing kinases involved in the pathogenesis of CML and related leukemias and has been effective in initial trials in solid tumors as well. At least one kinase serves as a distinct target in CML, ie Bcr-Abl, and this dosage of dasatinib is sufficient to suppress its activity. Although kinase activity is clearly implicated in the pathogenetic mechanisms implicated in scleroderma, including the production of

Approved v7.0 930029550 5.0

extracellular matrix components and fibrosis (fibroblasts and cytokine-producing cells), as well as in the activities of immune cells of the acquired (B and T-cells) and innate (monocytes, mast cells) immune systems, the precise kinase(s) target(s) in scleroderma are not known. The dosage of dasatinib proposed is capable of inhibiting kinase targets in all of these cells. Thus, this dose represents a prudent choice for anticipated safety and tolerability, as well as efficacy.

1.4 Study Rationale

The purpose of this open-label study is to evaluate the safety of dasatinib in the treatment of patients with moderate to severe scleroderma-related pulmonary fibrosis, to assure that there is not an unacceptable profile of toxicities, eg pleural and pericardial effusions, peripheral edema or myelosuppression. The local investigator may discontinue dasatinib based on the occurrence of a sufficiently significant drug-related toxicity. If a drug-related toxicity is encountered which the clinician determines is of a severity that continuation of dasatinib is possible at a lower dose, a pre-specific schedule with established thresholds for dose decrements is provided within the protocol.

Study discontinuation rules are provided within the protocol (see Section 1.5).

The 100 mg PO QD dose (recently approved for CML and under current study for solid tumors) was chosen because it was as effective as, and much less toxic than, the 70 mg PO BID dose previously approved for CML. No other drugs will be utilized for the treatment of scleroderma during this study.

1.5 Overall Risk/Benefit Assessment

There are no clinical data regarding safety or efficacy of dasatinib in the treatment of scleroderma. Based on preclinical data, a tyrosine kinase inhibitor like dasatinib (with both anti-fibrogenic and immunomodulatory potential) has a high likelihood of being effective in halting, perhaps reversing, this disorder. Currently there is no established, safe treatment for scleroderma. Once severe pulmonary fibrosis is present it progresses; progressive pulmonary disease is now the most common cause of death in scleroderma. Currently, the only therapeutic options are stem cell transplant (occasionally effective in small studies, if given early in the course of the progression), lung transplant (of

Approved v7.0 930029550 5.0

unproven value) and cyclophosphamide (efficacy in one study was statistically, but not clinically, significant).

Demonstration that dasatinib is well-tolerated and has the ability to halt progression of pulmonary fibrosis and other manifestations of scleroderma, would be a major contribution to management of these patients. Moreover there is reason to believe that this agent might reverse lung remodeling, which would be of tremendous benefit to patients with scleroderma. This would be a potentially a life-threatening intervention and certainly one that will eventuate in improvement of quality of life and the ability to discontinue potentially dangerous medication, eg cyclophosphamide.

The potential toxicities of dasatinib in scleroderma patients are currently unknown. Given the inexorable course of scleroderma lung disease we believe the potential benefits of the chosen dose of dasatinib, 100 mg PO QD (the lowest approved dasatinib dose for its primary indication) far outweigh the potential risks. We will not explore lower doses which would unduly delay testing the dose we believe (vide supra) to be most likely to demonstrate efficacy. Any such delay represents a potentially significant loss to scleroderma patients.

We are cognizant of the potential for known (and unknown) dasatinib toxicities to complicate the natural history of scleroderma. The current trial contains an intensive monitoring plan to rapidly identify the dasatinib toxicities manifest in all oncology studies, ie peripheral edema, pleural effusions, pericardial effusions. In the solid malignancy program severe hematologic toxicity was not encountered; in the CML trials this was an established toxicity, but is thought to be related to the immune milieu of CML, and/or myelophthysis (replacement of normal bone marrow components) and therefore not expected in the scleroderma population. Nonetheless, at all visits all scleroderma subjects will be monitored for myelosuppression. The routine management of scleroderma patients includes attention to all organ systems; the investigators are all clinicians with extensive experience in management of the multisystem features of scleroderma and will assess subjects at all visits. This clinical acumen will be supplemented by use of non-invasive imaging, eg echocardiography, chest radiography, electrocardiography, at predefined visits, in order to detect toxicities, eg pericardial effusion, at a preclinical stage.

Approved v7.0 930029550 5.0

The dasatinib Medical Surveillance Team (MST) will receive all reports of toxicities and CRFs rapidly and will discuss the status of the study at regular weekly meetings. Drug-related adverse events occurring more often than would be reasonable to expect by chance, ie the number of drug-related events descends below the 5% likelihood that a certain number of events would occur, as calculated from the Scleroderma Lung Study, will immediately require discussion by the MST to escalate the matter to consideration by the Medical Review Group (MRG), for possible study modification or termination.

The stopping rules for this study employed by the MST for this study are:

- All cause deaths of > 3
- Drug related deaths of 1 or more
- Any untreatable fluid retention adverse events of severity of CTC Grade III or IV that cause discontinuation from the study and do not reverse within 2 months of discontinuation of dasatinib.

There is no established and clinically significant therapy for the pulmonary fibrosis of scleroderma. Previous work has established that dasatinib's mechanism of action is a good fit for the complex pathophysiology of scleroderma, which involves fibrosis, vasculopathy and auto-immunity. Based on pre-clinical data, a dosage of dasatinib has been selected that will assure serum levels sufficient to suppress the target kinases for prolonged period of time each day. The possibility of drug toxicity remains, so a system has been developed to provide the intensive scrutiny necessary to identify adverse trends in individual patients and in the population as a whole.

2 STUDY OBJECTIVES

2.1 Primary Objective

The primary objective of this study is to evaluate the safety of dasatinib 100 mg PO in a population of subjects with scleroderma pulmonary fibrosis (see section 8.3 for endpoint definitions).

Approved v7.0 930029550 5.0

2.2 Exploratory Objectives

1) To estimate changes in FVC
2) To estimate changes in pulmonary fibrosis, as determined by semi-quantitative scoring of high-resolution CT scan of the chest
3) To estimate changes in Mahler dyspnea score
4) To estimate changes in the modified Rodnan skin score
5) To estimate changes in formal testing of D_LCO, TLC and FEV_1
6) To estimate changes in several pharmacodynamic measures (see Section 6.6 for complete list)
7) To assess pharmacokinetics of dasatinib in subjects with scleroderma

3 ETHICAL CONSIDERATIONS

3.1 Good Clinical Practice

This study will be conducted in accordance with Good Clinical Practice (GCP), as defined by the International Conference on Harmonisation (ICH) and in accordance with the ethical principles underlying European Union Directive 2001/20/EC and the United States Code of Federal Regulations, Title 21, Part 50 (21CFR50).

The study will be conducted in compliance with the protocol. The protocol and any amendments and the subject informed consent will receive Institutional Review Board/Independent Ethics Committee (IRB/IEC) approval/favorable opinion prior to initiation of the study.

All potential serious breaches must be reported to BMS immediately. A serious breach is a breach of the conditions and principles of GCP in connection with the study or the protocol, which is likely to affect, to a significant degree, the safety or physical or mental integrity of the subjects of the study or the scientific value of the study.

Study personnel involved in conducting this study will be qualified by education, training, and experience to perform their respective task(s).

Approved v7.0 930029550 5.0

This study will not use the services of study personnel where sanctions have been invoked or where there has been scientific misconduct or fraud (eg, loss of medical licensure, debarment).

Systems with procedures that assure the quality of every aspect of the study will be implemented.

3.2 Institutional Review Board/Independent Ethics Committee

Before study initiation, the investigator must have written and dated approval/favorable opinion from the IRB/IEC for the protocol, consent form, subject recruitment materials/process (eg, advertisements), and any other written information to be provided to subjects. The investigator or sponsor should also provide the IRB/IEC with a copy of the Investigator Brochure or product labeling, information to be provided to subjects and any updates.

The investigator or sponsor should provide the IRB/IEC with reports, updates and other information (eg, expedited safety reports, amendments, and administrative letters) according to regulatory requirements or institution procedures.

3.3 Informed Consent

Investigators must ensure that subjects, or, in those situations where consent cannot be given by subjects, their legally acceptable representatives, are clearly and fully informed about the purpose, potential risks, and other critical issues regarding clinical studies in which they volunteer to participate. Freely given written informed consent must be obtained from every subject or, in those situations where consent cannot be given by subjects, their legally acceptable representative, prior to clinical study participation, including informed consent for any screening procedures conducted to establish subject eligibility for the study.

The rights, safety, and well-being of the study subjects are the most important considerations and should prevail over interests of science and society.

Approved v7.0 930029550 5.0

Appendix 1 contains BMS procedures on obtaining informed consent from subjects, or, in those situations where consent cannot be given by subjects, their legally acceptable representative prior to participating in a clinical study. Procedures are described for all subjects, including those who are unable to give informed consent. The relevant procedures must be used whenever they are applicable (see subject selection criteria in Sections 4.2.1 and 4.2.2).

4 INVESTIGATIONAL PLAN

4.1 Study Design and Duration

This is a 30 subject, United States only, open label, safety study.

The duration of dasatinib dosing in this study will be 2 years. The primary study period will end for each subject when he or she completes six months of treatment unless the study is terminated for safety reasons. Subjects will be allowed to continue dasatinib beyond the 6 month period of the trial in order to assess the longer term safety and efficacy of dasatinib if, in the judgment of the subject and the investigator, the subject is deriving clinical benefit from therapy, is not experiencing any toxicity that precludes continuing therapy at the given dose being tolerated and the study has not been terminated by the sponsor. One follow up phone call will occur approximately 28 days after the last dose of study drug to assess adverse events.

4.2 Study Population

Patients to be recruited will be at least 18 years of age with established diffuse cutaneous scleroderma with a modified Rodnan skin score of at least 15 and evidence of fibrosing alveolitis as defined below on pulmonary function AND high resolution computed tomography (HRCT) scan of the chest/lungs

For entry into the study, the following criteria MUST be met:

Approved v 7.0 930029550 5.0

4.2.1 Inclusion Criteria

1) **Signed Written Informed Consent**
   a) Subject is willing to participate in the study and signs informed consent document

2) **Target Population**
   a) meet ACR criteria for scleroderma
   b) have clinical evidence of active skin disease with a skin score of ≥ 15 (see appendix 3)
   c) have had the onset of their first non-Raynaud phenomenon feature of SSc no more than 3 years prior to screening
   d) have evidence of fibrosing alveolitis (active pulmonary fibrosis) manifested by a Forced Vital Capacity (FVC) between 45% and 80% of predicted normal and/or a Diffusing Capacity for Carbon Monoxide (D_LCO) between 30% and 70% of predicted normal
   e) have an abnormal high resolution CT scan of the chest/lungs demonstrating typical ground glass changes of alveolitis with background fibrosis
   f) have adequate renal function- no evidence of renal crisis in the 2 months prior to enrollment and serum creatinine < 3 mg/dL
   g) for both sexes, must use an acceptable form of birth control

3) **Age and Sex**
   a) Men and women, ages ≥ 18

   Women of childbearing potential (WOCBP) must be using an adequate method of contraception to avoid pregnancy throughout the study and for up to 12 weeks after the last dose of investigational product in such a manner that the risk of pregnancy is minimized.

   WOCBP include any female who has experienced menarche and who has not undergone successful surgical sterilization (hysterectomy, bilateral tubal ligation, or bilateral oophorectomy) or is not postmenopausal. Post menopause is defined as:

   - Amenorrhea ≥ 12 consecutive months without another cause
   - For women with irregular menstrual periods and on hormone replacement therapy (HRT), a documented serum follicle stimulating hormone (FSH) level ≥ 35 mIU/mL.

Approved v7.0 930029550 5.0

Women who are using oral contraceptives, other hormonal contraceptives (vaginal products, skin patches, or implanted or injectable products), or mechanical products such as an intrauterine device or barrier methods (diaphragm, condoms, spermicides) to prevent pregnancy, or are practicing abstinence or where their partner is sterile (eg, vasectomy) should be considered to be of childbearing potential.

WOCBP must have a negative serum or urine pregnancy test (minimum sensitivity 25 IU/L or equivalent units of HCG) within 48 hours prior to the start of investigational product.

4.2.2 Exclusion Criteria

1) **Sex and Reproductive Status**
   a) WOCBP who are **unwilling or unable** to use an acceptable method to avoid pregnancy for the entire study period and for up to 12 weeks after the last dose of investigational product.
   b) WOCBP using a prohibited contraceptive method (Not Applicable for this study), Women who are pregnant or breastfeeding.
   c) Women with a positive pregnancy test on enrollment or prior to investigational product administration.
   d) Men whose sexual partners are WOCBP, who are unwilling or unable to use an acceptable method to avoid pregnancy for the entire study period and for at least one month (4 weeks) after completion of study medication.

2) **Target Disease Exceptions**
   a) a primary diagnosis of limited scleroderma
   b) a scleroderma-like illness associated with environmental or ingested agents, eg toxic rapeseed oil, vinyl chloride, bleomycin.

3) **Medical History and Concurrent Diseases**
   a) Clinically significant pleural or pericardial effusion in the previous 12 months: Common Terminology Criteria for Adverse Events (CTCAE guidelines will be supplied in a separate document) Grade 3 or 4. Patients with recent Grade I or II effusions or peripheral edema will be permitted to enter the study.
   b) Clinically significant cardiac disease (New York Heart Association Class III or IV see appendix 4) including preexisting arrhythmia, (such as ventricular tachycardia, ventricular fibrillation, or “Torsade de Pointes”), myocardial

Approved v7.0 930029550 5.0

infarction, uncontrolled angina within 6 months, congestive heart failure, cardiomyopathy, or pericardial disease.

c) Clinically-significant coagulation or platelet function disorder (eg, known von Willebrand's disease).

d) Abnormal QTcF interval prolonged (≥ 450 msec) after electrolytes have been corrected on baseline ECG.

e) The presence of another underlying disease that currently requires or might be expected to require therapy with > 10mg of prednisone-equivalent per day.

f) History of malignancy other than basal cell carcinoma, fewer than 3 squamous cell carcinomas, or cervical carcinoma in situ treated with conization or other definitive therapy.

4) Physical and Laboratory Test Findings

a) Hgb < 10 g/dL; platelet count < 100x10^9/L: WBC < 3.0x10^9/L; PMN < 1.0x10^9/L; OR lymphocytes < 3.5x10^9/L.

b) The presence of any of the following laboratory findings at screening: positive for antibodies to hepatitis C virus; positive for antibodies to hepatitis B surface antigen (HBsAg); serum bilirubin ≥ 2 times normal, ALT or AST > 2.5 times upper limit of normal

c) Subjects with evidence (as assessed by the investigator) of active or latent bacterial or viral infection at the time of potential enrollment, including subjects with evidence of Human Immunodeficiency Virus (HIV) infection.

5) Allergies and Adverse Drug Reactions

None Known

6) Prohibited Treatments and/or Therapies

a) subjects taking drugs generally accepted to have a risk of causing Torsades de Pointes (see category 1 at: http://www.qtdrugs.org/medical-pros/drug-lists/drug-lists.htm)

b) use of other immunosuppressive therapies must be discontinued at enrollement, eg methotrexate, azathioprine, cyclophosphamide, mycophenolic acid, mycophenolate mofetil, cyclosporine

c) treatment with any other experimental or investigational drug(s) concurrently or less than 12 weeks prior to study enrollment

d) use of anti-fibrotic agents must be discontinued at enrollment, eg colchicine, D-penicillamine, minocycline or Type 1 oral collagen

Approved v7.0 930029550 5.0

e) current or prior use of endothelin 1 receptors eg Bosentan, prior exposure to dasatinib, imatinib, or any other tyrosine kinase inhibitor

See Section 5.5.1

7) Other Exclusion Criteria

a) Prisoners or subjects who are involuntarily incarcerated
b) Subjects who are compulsorily detained for treatment of either a psychiatric or physical (eg, infectious disease) illness

Eligibility criteria for this study have been carefully considered to ensure the safety of the study subjects and to ensure that the results of the study can be used. It is imperative that subjects fully meet all eligibility criteria.

4.2.3 Discontinuation of Subjects from Treatment

Subjects MUST discontinue study treatment (investigational or noninvestigational treatment) for any of the following reasons:

- Withdrawal of informed consent (subject’s decision to withdraw for any reason)
- Any clinical adverse event (AE), laboratory abnormality or intercurrent illness which, in the opinion of the investigator, indicates that continued participation in the study is not in the best interest of the subject
- Pregnancy (see Section 7.6.2)
- Termination of the study by Bristol-Myers Squibb (BMS)
- Loss of ability to freely provide consent through imprisonment or involuntarily incarceration for treatment of either a psychiatric or physical (eg, infectious disease) illness
- Hemodynamically significant pericardial effusion by echocardiogram or physical examination that presents a significant risk to the patient's well-being and persists despite medical management.
- Progressive and/or significant pleural effusion by chest x-ray (CXR) or HRCT or physical examination
- Significant peripheral edema that presents a significant risk to the patient's well-being and persists despite medical management.
- Significant worsening of underlying scleroderma.

Approved v7.0 930029550 5.0

All Adverse Events will be graded according to Common Terminology Criteria for Adverse Events.

All subjects who discontinue study treatment should comply with protocol specified procedures as outlined in the time and events schedule (Section 6.1) day 729/early termination. The only exception to this requirement is when a subject withdraws consent for all study procedures or loses the ability to consent freely (eg, is imprisoned or involuntarily incarcerated for the treatment of either a psychiatric or physical illness).

If a subject was withdrawn before completing the study, the reason for withdrawal must be entered on the appropriate case report form (eCRF) page.

4.3 Data Monitoring Committee

Not applicable

5 TREATMENTS

5.1 Study Treatment

5.1.1 Investigational Product

An investigational product, also known as investigational medicinal product in some regions, is defined as follows:

A pharmaceutical form of an active substance or placebo being tested or used as a reference in a clinical study, including products already with a marketing authorization but used or assembled (formulated or packaged) in a way different from the authorized form, or used for an unauthorized indication, or when used to gain further information about the authorized form.

In this protocol, investigational product(s) is/are:

Dasatinib (SPRYCEL®) 20 mg and 50 mg tablets

Approved v7.0 930029550 5.0

5.1.2 Noninvestigational Product

Other medications used in the study as support or escape medication for preventative, diagnostic, or therapeutic reasons, as components of the standard of care for a given diagnosis, are considered noninvestigational products.

5.1.3 Identification

Dasatinib will be supplied by Bristol-Myers Squibb Research and Development in two different strengths:

Table 5.1.3: Identification

PRODUCT	APPEARANCE
Dasatinib 20 mg	film coated tablets, biconvex, round, white to off-white in appearance with “20” or “BMS”debossed on one side and “527” on the other side
Dasatinib 50 mg	film coated tablets, biconvex, oval, white to off-white in appearance with “50” or “BMS” debossed on one side and “528” on the other side

5.1.4 Packaging and Labeling

Dasatinib will be labeled in open-label fashion. Description of the contents, batch number, container number, storage conditions and caution statements required by country regulations will be on the label.

The second panel will have the batch number and container number.

Study medications will be packaged as follows:

- Dasatinib 20 mg film coated tablets, 30 tabs/bottle
- Dasatinib 50 mg film coated tablets, 30 tabs/bottle.

5.1.5 Handling and Dispensing

The investigational product should be stored in a secure area according to local regulations. It is the responsibility of the investigator to ensure that investigational

Approved v7.0 930029550 5.0

product is only dispensed to study subjects. The investigational product must be dispensed only from official study sites by authorized personnel according to local regulations. Bristol-Myers Squibb will be responsible for assuring that the quality of dasatinib is adequate for the duration of the study.

The investigator should ensure that the investigational product is stored in accordance with the environmental conditions (temperature, light, and humidity) as determined by the sponsor. Dasatinib should be stored in a secure area, at 59°F to 77°F (15°C to 25°C). If concerns regarding the quality or appearance of the investigational product arise, do not dispense the investigational product and contact the sponsor immediately.

The investigator (or assigned designee, eg, study pharmacist) will dispense the proper number of each strength tablet to the subject to satisfy dosing requirements until the subject's next visit.

The subject must be instructed to return all unused study medications in the provided packaging at each subsequent visit.

Procedures for proper handling and disposal of anticancer drugs should be considered.

Dasatinib tablets consist of a core tablet (containing the active drug substance), surrounded by a film coating to prevent exposure of pharmacy and clinical personnel to the active drug substance. However, if the tablets are crushed or broken, pharmacy and clinical personnel should wear disposable chemotherapy gloves. Personnel who are pregnant should avoid exposure to crushed and/or broken tablets.

The investigator must be satisfied the subject returned or accounted for all unused medication before additional medication is dispensed. If the number of tablets used is substantially different from the number of tablets dispensed, the subject must be counseled on how study therapy should be taken. If such deviations persist, the investigator may consider discontinuing the subject for non-compliance.

Please refer to Section 9.2.2 for information on investigational product record retention and 9.3 for return and destruction instructions.

Approved v7.0 930029550 5.0

5.2 Method of Assigning Subjects to a Treatment

This is an open label study and all subjects will receive dasatinib 100 mg PO QD. Subjects will be enrolled by a phone call to an Interactive Voice Response System (IVRS) after informed consent is obtained and eligibility confirmed. A patient identification number (PID) to be used for all subsequent communications will be assigned. Instructions on the use of interactive voice response system will be provided at site initiation and in a Procedure Manual.

5.3 Selection and Timing of Dose for Each Subject

Dasatinib 100mg (two 50mg tablets) will be taken daily. Dose may be reduced at a later time in case of toxicity. Dasatinib absorption is highly dependent on the presence of a low gastric pH. Thus therapy with protein pump inhibitors (PPIs) or histamine receptor H2) blockers should not be commenced after a subject enters this study. If a subject is taking these agents prior to study start the agent and dosage should not be changed while the subject is in the study and should be taken at least 2 hours after the dasatinib dose.

5.3.1 Dose Modifications

Common dasatinib toxicities in both leukemia and solid tumor studies include bone marrow suppression and fluid retention, including effusions (pleural and pericardial) and peripheral edema. Bone marrow suppression is far less common in studies of solid tumors.

If, in the judgment of the investigator, significant dasatinib-related bone marrow suppression occurs, the dasatinib dosage may be decreased utilizing the following scheme:

100 mg qd → 70 mg qd→ 50 mg qd→ 20 mg qd→ discontinue dosing.

If potentially life-threatening myelosuppression develops, eg ANC < 0.5 x 10^9/L or Platelets < 50 x 10^9/L, dasatinib should be stopped until ANC > 1.0 x 10^9/L, and platelets > 50 x 10^9/L, when treatment can be resumed with dasatinib at a lower dose.

Approved v7.0 930029550 5.0

Fluid retention due to dasatinib, in prior hematology and oncology trials, has been successfully managed with diuresis and has occasionally benefited from a short course of corticosteroids. If in the current trial this approach is not successful, or if the fluid retention is of greater concern, the dasatinib dosage may be decreased following the dosing approach outlined above. If the fluid retention persists at the lower dose, a further decrease should be considered within 7 days.

Should other, non-anticipated adverse events, occur that are, in the judgment of the investigator, likely due to dasatinib, the dasatinib dosage may be decreased following the dosing approach outlined above. If the adverse event persists at the lower dose, a further decrease should be considered within 7 days. If an adverse event does not resolve after a subject's dose has been decreased to 20 mg, the subject must then be discontinued.

5.4 Blinding/Unblinding

Not Applicable

5.5 Concomitant Treatments

Prior use of other immunosuppressive therapies must have been terminated at enrollment; these agents include methotrexate, azathioprine, cyclophosphamide, mycophenolic acid, mycophenolate mofetil, and cyclosporine. None of these agents can be taken concomitantly with dasatinib.

Likewise, the use of any anti-fibrotic agents must have been terminated at enrollment; these agents include colchicine, D-penicillamine, minocycline and Type 1 oral collagen.

Prednisone may be continued at a stable dose of no more than 10 mg a day, if prednisone had been part of the subject's prior therapeutic regimen for at least 1 month prior to signing informed consent; prednisone dosage cannot have been changed within 1 month of signing informed consent. Likewise, subjects may continue non-steroidal anti-inflammatory agents and COX-2 inhibitors, eg celecoxib, that had been part of their pre-study therapy, but with no modification of dosing throughout the study unless it is a decrease in dosage for toxicity.

Approved v7.0 930029550 5.0

5.5.1 Prohibited and/or Restricted Treatments

The investigator must discuss with the Medical Monitor any plans to use prohibited/restricted treatments during the study, unless there is emergent need to utilize such agent(s).

5.5.1.1 *Medications that Prolong QT_C Interval*

Subjects enrolled in this study should not take or commence therapy with concomitant medications known to prolong the QTc interval. Should the investigator believe that therapy with an agent that may prolong the QTc (other than those explicitly prohibited) is vital to an individual subject's care, the investigator must check that the subject's prior on-therapy ECG has not shown a QTcF ≤ 450 msec or an increase in QTc ≥ 60 msec over the baseline value. For such medications, a wash-out period of ≥ 7days is required prior to starting dasatinib. For Category 1 risk medications known to prolong the QTc interval refer to http://www.qtdrugs.org/medical-pros/drug-lists/drug-lists.htm.

5.5.2 Other Restrictions and Precautions

Restricted medications should be avoided during concomitant dasatinib use. Their use is not strictly prohibited. If there is a clinically significant indication to use one of these medications, the following precautions should be recognized.

5.5.2.1 *Medications that Inhibit Platelet Function and Anticoagulants*

Subjects enrolled in this study should avoid taking concomitant medications that durably inhibit platelet function in the setting of a grade 3 or 4 thrombocytopenia. Subjects who require these medications should be monitored closely for thrombocytopenia and bleeding and discontinuation of these medications should be considered in the setting of grade 3 - 4 thrombocytopenia.

Medications that directly and durably inhibit platelet function include:

- aspirin or aspirin-containing combinations, clopidogrel, dipyridamole
- tirofiban, epoprostenol, eptifibatide, cilostazol, abciximab, ticlopidine, cilostazol.

Approved v7.0 930029550 5.0

Medications that directly and durably inhibit coagulation include:

- warfarin, heparin/low molecular weight heparin (eg, danaparoid, dalteparin, tinzaparin, enoxaparin).

5.5.2.2 CYP3A4 Inhibitors

Dasatinib is primarily metabolized by the CYP3A4 enzyme. In a study of 18 subjects with solid tumors, 20 mg dasatinib QD co-administered with 200 mg of ketaconazole BID increased the dasatinib Cmax and AUC by four- and five- fold, respectively. Therefore, potent inhibitors of CYP3A4 are restricted during study. These include ketoconazole, itraconazole, erythromycin, clarithromycin, ritonavir, atazanavir, indinavir, nefazodone, nelfinavir, saquinavir, telithromycin. For this same reason, subjects should not consume substantial quantities of grapefruit juice.

Any of these may increase dasatinib plasma concentration and should be avoided.

Subjects receiving dasatinib, should be closely monitored for toxicity and a dasatinib dose reduction should be considered if systemic administration of a potent CYP3A4 inhibitor cannot be avoided. Also refer to Appendix 5 of this protocol.

5.5.2.3 CYP3A4 Inducers

Drugs that induce CYP3A4 activity may decrease dasatinib plasma concentrations. Data from a study of 20 healthy subjects indicate that when a single morning dose of dasatinib was administered following 8 days of continuous evening administration of 600 mg of rifampicin, a potent CYP3A4 inducer, the mean Cmax and AUC of dasatinib were decreased by 81% and 82%, respectively. In subjects in whom CYP3A4 inducers (eg, phenytoin, carbamazepine, rifampicin, phenobarbital) are absolutely indicated, alternative agents with less enzyme induction potential must be used. Also refer to appendix 5 of this protocol.

5.5.2.4 CYP3A4 Substrates

CYP3A4 substrates may have their plasma concentration altered by dasatinib. Single dose data from a study of 54 healthy subjects indicate that the mean Cmax and AUC of

Approved v7.0 930029550 5.0

simvastatin, a CYP3A4 substrate, were increased by 37% and 20%, respectively, when simvastatin was administered in combination with a single 100 mg dose of dasatinib. Drugs metabolized in the CYP3A4 system that are known to have a narrow therapeutic index such as alfentanil, astemizole, terfenadine, cisapride, cyclosporine, fentanyl, pimozide, quinidine, sirolimus, tacrolimus, or ergot alkaloids (ergotamine, dihydroergotamine) should be administered with caution in subjects receiving dasatinib. Also refer to Appendix 5 of this protocol.

5.5.2.5 Antacids

Nonclinical data demonstrate that the solubility of dasatinib is pH dependent. In a study of 24 healthy subjects, administration of 30mL aluminum hydroxide/magnesium hydroxide 2 hours prior to a single 50 mg dose of dasatinib was associated with no relevant change in dasatinib AUC; however, the dasatinib Cmax increased 26%. When 30mL of aluminum hydroxide/magnesium hydroxide was administered to the same subjects concomitantly with a 50 mg dose of dasatinib, a 55% reduction in dasatinib AUC and a 58% reduction in Cmax were observed. Simultaneous administration of dasatinib with antacids should be avoided. If antacid therapy is needed, the antacid dose should be administered at least 2 hours after the dose of dasatinib.

5.5.2.6 H2 Blockers/Proton Pump Inhibitors

Long-term suppression of gastric acid secretion by H2 blockers or proton pump inhibitors (eg, famotidine and omeprazole) is likely to reduce dasatinib exposure. In a study of 24 healthy subjects, administration of a single 50 mg dose of dasatinib 10 hours following famotidine reduced the AUC and Cmax of dasatinib by 61% and 63%, respectively. The concomitant use of H2 blockers or proton pump inhibitors with dasatinib is not recommended. The use of antacids should be considered in place of H2 blockers or proton pump inhibitors in subjects receiving dasatinib therapy. However, if the use of H2 blockers or proton pump inhibitors is necessary, these agents should be taken at least 2 hours following the dasatinib dose.

Approved v7.0 930029550 5.0

5.6 Treatment Compliance

Starting at Day 1, all treated subjects will be assessed for drug compliance of dasatinib. Subjects will return study drug containers at clinic visits. Treatment compliance will be monitored by drug accountability and recorded in the subject's medical record and on the CRF.

6 STUDY ASSESSMENTS AND PROCEDURES

6.1 Flow Chart/Time and Events Schedule

A visit window of ± 3 days is allowed for each visit with the exception of Day 15. A ± 1 day visit window is allowed at Day 15. Visits will be monthly until Day 365 and then quarterly until Day 729. WOCBP will be provided with pregnancy test kits for the months between the visits.

Approved v7.0 930029550 5.0

Table 6.1A: Flow Chart/Time and Events Schedule Protocol CA180267

**Visit Day**	**Screening**	**Day 1**	**Day 15**	**Day 29**	**Day 57**	**Day 85**	**Day 113**	**Day 141**	**Day 169**	**Protocol Sections**
Informed Consent	X									3.3
Inclusion/Exclusion Criteria	X									4.2
Enroll Subject (contact IVRS)	X									5.2
Medical History	X									4.2.1/4.2.2
Complete Physical Exam	X								X	6.3
Brief Physical Exam (see Section 6.3)		X	X	X	X	X	X	X		6.3
Vital Signs (Heart Rate and Blood Pressure, after subject has been in the sitting position for 5 minutes)	X	X	X	X	X	X	X	X	X	6.3
ECG	X		X	X						4.2.2/6.3
High Resolution CT Scan of the Chest	X								X	4.2.2/6.3
Chest X Ray			X							4.2.2/6.3
Echocardiogram	X			X		X		X		4.2.2/6.3
**Efficacy Assessments**										6.4
Skin biopsy		X							X	4.2.2/6.4
Modified Rodnan skin score	X								X	4.2.2/6.4
Pulmonary Function Testing	X					X			X	4.2.2/6.4
Mahler Dyspnea Index		X							X	6.4

Approved v7.0 930029550 5.0

Table 6.1A: Flow Chart/Time and Events Schedule Protocol CA180267

**Visit Day**	**Screening**	**Day 1**	**Day 15**	**Day 29**	**Day 57**	**Day 85**	**Day 113**	**Day 141**	**Day 169**	**Protocol Sections**
**Laboratory Assessments**										6.3.1
CBC with Platelets	X	X	X	X	X	X	X	X	X	4.2.2/6.3.1
Chemistry panel	X	X	X	X	X	X	X	X	X	4.2.2/6.3.1
Hepatitis Screening	X									4.2.2/6.3.1
PT/PTT	X									4.2.2/6.3.1
Urinalysis	X	X	X	X	X	X	X	X	X	4.2.2/6.3.1
Urine/serum pregnancy test (WOCBP only)	X	X		X	X	X	X	X	X	6.3
Pharmacokinetic Assessments (see Section 6.5)			X							6.5
Pharmacodynamic Assessments (see Section 6.6)		X	X						X	6.6
Dispense Drug		X	X	X	X	X	X	X	X	5

Approved v7.0 930029550 5.0

Table 6.1B: Months 6 - 12 Flow Chart/Time and Events Schedule Protocol CA180267

**Visit Day**	**Day 197**	**Day 225**	**Day 253**	**Day 281**	**Day 309**	**Day 337**	**Day 365**	**Protocol Sections**
Complete Physical Exam							X	
Brief Physical Exam (see Section 6.3)	X	X	X	X	X	X		6.3
Vital Signs (Heart Rate and Blood Pressure, after subject has been in the sitting position for 5 minutes)	X	X	X	X	X	X	X	6.3
High Resolution CT Scan of the Chest							X	4.2.2/6.3
Echocardiogram	X		X		X			4.2.2/6.3
**Efficacy Assessments**								6.4
Skin biopsy								4.2.2/6.4
Modified Rodnan skin score							X	4.2.2/6.4
Pulmonary Function Testing							X	4.2.2/6.4
Mahler Dyspnea Index							X	6.4
**Laboratory Assessments**								6.3.1
CBC with Platelets	X	X	X	X	X	X	X	4.2.2/6.3.1
Chemistry panel	X	X	X	X	X	X	X	4.2.2/6.3.1
Urinalysis	X	X	X	X	X	X	X	4.2.2/6.3.1
Urine/serum pregnancy test (WOCBP only)	X	X	X	X	X	X	X	6.3

Approved v7.0 930029550 5.0

Table 6.1B: Months 6 - 12 Flow Chart/Time and Events Schedule Protocol CA180267

Visit Day	Day 197	Day 225	Day 253	Day 281	Day 309	Day 337	Day 365	Protocol Sections
Pharmacodynamic Assessments (see Section 6.6, no assessments during this time period)								6.6
Dispense Drug	X	X	X	X	X	X	X	5

Table 6.1C: Flow Chart/Time and Events Schedule Protocol CA180267

Visit Day	Day 449	Day 533	Day 617	Day 729 /Early Term	Day 757/ 28 Days past Last Dose	Protocol Sections
Complete Physical Exam				X		6.3
Brief Physical Exam (see Section 6.3)	X	X	X			6.3
Vital Signs (Heart Rate and Blood Pressure, after subject has been in the sitting position for 5 minutes)	X	X	X	X		6.3
High Resolution CT Scan of the Chest				X		4.2.2/6.3
Echocardiogram		X				4.2.2/6.3
**Efficacy Assessments**						6.4
Skin biopsy				X		4.2.2/6.4
Modified Rodnan skin score		X		X		4.2.2/6.4
Pulmonary Function Testing		X		X		4.2.2/6.4
Mahler Dyspnea Index		X		X		6.4
**Laboratory Assessments**						6.3.1
CBC with Platelets	X	X	X	X		4.2.2/6.3.1
Chemistry panel	X	X	X	X		4.2.2/6.3.1
Urinalysis	X	X	X	X		4.2.2/6.3.1
Urine/serum pregnancy test (WOCBP only)[a]	X	X	X	X		6.3

Approved v7.0 930029550 5.0

Table 6.1C: Flow Chart/Time and Events Schedule Protocol CA180267

Visit Day	Day 449	Day 533	Day 617	Day 729 /Early Term	Day 757/ 28 Days past Last Dose	Protocol Sections
Pharmacodynamic Assessments (see Section 6.6)<br>Only Samples for Exploratory RNA Profiling will be collected at day 729.				X		6.6
Dispense Drug	X	X	X			5
Follow Up Phone Visit					X	6.3.2

[a] Visits are quarterly after Day 365. At the interim visits, the Women of Child Bearing Potential (WOCBP) subjects will be provided with pregnancy test kits, to be used monthly and instructed to contact their study office to report the results of the pregnancy test.

Approved v7.0 930029550 5.0

6.2 Study Materials

Subject appointment cards; Pregnancy Prevention Information Sheets; Serious Adverse Event Forms; Mahler Dyspnea CDROM; pocket sized protocols and Common Terminology Criteria for Adverse Events (CTCAE) guides; source document worksheets to assist in data entry and; laminated Time and Events Schedule.

6.3 Safety Assessments

All subjects who receive a dose of study drug will be evaluated for safety. Safety outcomes include adverse events, clinically significant changes in vital signs, and laboratory test abnormalities. The Investigator will rate the severity of each adverse event as mild, moderate, severe, or very severe and grade the event using Common Terminology Criteria for Adverse Events. Lab findings which the Investigator feels are clinically relevant based on laboratory guidelines in appendix 6 should be recorded as adverse events. In addition, the Investigator will determine the relationship of the adverse event to the administration of the study drug.

Complete and/or brief physical examinations may be performed by a Doctor of Medicine (MD), Doctor of Osteopathy (DO), Physician’s Assistant (PA), or Nurse Practitioner (NP). While the interim physical exam may not be as comprehensive as the initial full examination, important body systems should be included as clinically indicated. These body systems can include lymph nodes, liver, spleen, and breast, at the discretion of the examiner. An interim physical examination may note any changes in the subject’s condition since the last assessment and does not preclude examination of any of the body systems as clinically indicated.

If during any physical exam signs and symptoms of a pleural effusion are noted obtaining a chest x-ray is recommended. If it is therapeutically necessary to perform a thoracentesis the pleural fluid analysis will be captured on a supplemental eCRF.

The following safety assessments will be done according to the time and events schedules in Section 6.1:

Complete Medical History
Complete Physical Exam/Brief Physical Exam

Approved v7.0 930029550 5.0

Echocardiogram
Chest Xray
High Resolution CT Scan of the chest
ECG
Vital Signs (heart rate and blood pressure)

6.3.1 Laboratory Assessments to include:

The following laboratory assessments will be done according to the time and events schedules in Section 6.1 Detailed instructions concerning processing and shipping will be included in a separate manual.

Hematology

Hemoglobin
Hematocrit
RBC
Total WBC count, including differential
Platelet count
PT/PTT

Blood Chemistry:

Sodium	Creatinine
Potassium	Blood urea nitrogen (BUN)
Chloride	Total bilirubin
Total Protein	Alanine aminotransferase (ALT)
Albumin	Aspartate aminotransferase (AST)
Calcium	Gamma-glutamyltransferase (GGT)
Phosphorus	Alkaline phosphatase
Uric Acid	Glucose

Creatinine Kinase (CK) and troponin 1 will be tested on Day 1 and on Day 57 and Day 169 or early term if early term occurs prior to day 169. If the CPK is high a CKMB will be performed.

Approved v7.0 930029550 5.0

Urinalysis:

pH
Protein
Glucose
Blood

If blood, protein or glucose is positive on the dipstick a microscopic examination of the urine sediment should be performed.

Hepatitis screen: (performed at screening visit only)

Hepatitis B surface antigen	if positive, core antibody
Hepatitis C antibody	if positive, RIBA or PCR

In Addition:

Urine or serum pregnancy tests will be performed, for all WOCBP, within 48 hours of initial dose and at every visit thereafter with the exception of the DAY 15 visit.,. If any female subject becomes pregnant she will be immediately discharged from the study.

6.3.2 Follow Up Visits

All subjects will receive a follow up phone call approximately 28 days after the last dose of study drug to assess adverse events, whether or not any study related toxicities are present. Additional follow up visits will be required every 4 weeks until all study related toxicities are resolved to baseline values, stabilized, or are deemed irreversible.

6.4 Efficacy Assessments

The following efficacy assessments will be performed according to the time and events schedule in Section 6.1:

Pulmonary Function Testing (Forced Vital Capacity (FVC) and Diffusing Capacity (D_LCO).
Modified Rodnan Skin Score
Skin Biopsy
Mahler Dyspnea Index

Approved v7.0 930029550 5.0

6.5 Pharmacokinetic Assessments

The plasma pharmacokinetics of dasatinib will be determined for subjects on Day 15 of the study at a dose of 100 mg QD.

6.5.1 Pharmacokinetics: Blood Collection and Processing

The samples for pharmacokinetic assessment should be collected on the same day as the pharmacodynamic assessment of samples. Table 6.5.1 list the sampling schedule to be followed for the assessment of pharmacokinetics.

PK samples will be obtained at clinic visit, for which the subject will delay that day's dose until the subject is in clinic in order to obtain a pre-dose sample (a trough sample being essential). The time of prior dose must be recorded. PK sampling should be deferred to a subsequent visit if a daily dose was already taken or if interruption or dose adjustment is required.

Table 6.5.1: PK Sampling Schedule

**Sample Collection Time**		**Time (Relative To Dasatinib Dosing) hours:min**	**PK samples for Dasatinib**
Study Days	Time (Event Relative to Dasatinib Dosing)		
15	0 (pre-dose)	00.00 (pre-dose)	X
	0.5 h	00:30	X
	1 h	01:00	X
	1.5 h	01:30	X
	2 h	02:00	X
	3 h	03:00	X
	4	04.00	X
	6	06.00	X
	8	08.00	X

6.5.2 Pharmacokinetic Sample Analysis

Plasma samples will be analyzed for dasatinib by a validated liquid chromatography tandem mass spectrometry (LC-MS/MS).

Approved v 7.0 930029550 5.0

6.6 Pharmacodynamics Assessments

Blood, urine and skin biopsies will be collected prior to dosing for pharmacodynamic assessments on all treated subjects at the times specified in Section 6 of the protocol. Analysis of markers will be performed by a central/vendor laboratory or BMS using validated methodology. Complete details for the collection, processing, storing, and shipping of all pharmacodynamic samples will be provided in the laboratory procedures manual.

Remaining blood/serum/plasma/biopsy samples will be stored for a period of up to 3 years following last patient last visit, for possible analysis of additional markers. All samples will be destroyed within this 3 year period.

Assessments including, but not limited to, the following measures will be conducted:

- Plasma/Serum/Urine Soluble Mediators: CRP, IL-6, IL-8, IL-10, sIL-2R, sCTX-II, COMP, TGFbeta
- Exploratory RNA Profiling: Whole blood and skin samples will be studied utilizing microarray and/or qPCR to identify changes in RNA expression.

6.6.1 Plasma/Serum Soluble Mediators

Plasma and serum blood samples, as well as urine, will be collected for CRP, IL-6, IL-8, IL-10, sIL-2R, sCTX-II, COMP, TGFbeta levels and will be shipped to the central laboratory. Refer to Section 6 of the protocol for specified collection time points.

6.6.2 Exploratory RNA Profiling

Whole blood RNA samples will be collected in PAXgene and skin biopsy RNA samples will be collected into tubes containing RNAlater according to the specified time points in Section 6 of the protocol. The samples will then be labeled, frozen and transferred via the central laboratory to Bristol-Myers Squibb Sample Bank for storage and future analysis.

Approved v7.0 930029550 5.0

6.7 Pharmacogenomic/Pharmacogenetic Assessments

Refer to Protocol Amendment #1.

6.8 Outcomes Research Assessments

Not Applicable

6.9 Other Assessments

Not Applicable

7 ADVERSE EVENTS

7.1 Definitions

An ***Adverse Event (AE)*** is defined as any new untoward medical occurrence or worsening of a pre-existing medical condition in a subject or clinical investigation subject administered an investigational (medicinal) product and that does not necessarily have a causal relationship with this treatment. An AE can therefore be any unfavorable and unintended sign (including an abnormal laboratory finding, for example), symptom, or disease temporally associated with the use of investigational product, whether or not considered related to the investigational product.

7.1.1 Serious Adverse Events

A ***serious AE (SAE)*** is any untoward medical occurrence that at any dose:

- results in death
- is life-threatening (defined as an event in which the subject was at risk of death at the time of the event; it does not refer to an event which hypothetically might have caused death if it were more severe)
- requires inpatient hospitalization or causes prolongation of existing hospitalization (see note below for exceptions)
- results in persistent or significant disability/incapacity

Approved v7.0 930029550 5.0

- is a congenital anomaly/birth defect
- is an important medical event (defined as a medical event(s) that may not be immediately life-threatening or result in death or hospitalization but, based upon appropriate medical and scientific judgment, may jeopardize the subject or may require intervention [eg, medical, surgical] to prevent one of the other serious outcomes listed in the definition above.) Examples of such events include, but are not limited to, intensive treatment in an emergency room or at home for allergic bronchospasm; blood dyscrasias or convulsions that do not result in hospitalization.)

All pregnancies, regardless of outcome, must be reported to the sponsor on a Pregnancy Surveillance Form, not an SAE form (see Section 7.6). Although overdose and cancer are not always serious by regulatory definition, these events should be reported on an SAE form and sent to BMS in an expedited manner.

NOTE:

The following hospitalizations are not considered SAEs in BMS clinical studies:

- a visit to the emergency room or other hospital department < 24 hours, that does not result in admission (unless considered "important medical event" or event life threatening)
- elective surgery, planned prior to signing consent
- admissions as per protocol for a planned medical/surgical procedure
- routine health assessment requiring admission for baseline/trending of health status (eg, routine colonoscopy)
- medical/surgical admission for purpose other than remedying ill health state and was planned prior to entry into the study. Appropriate documentation is required in these cases
- admission encountered for another life circumstance that carries no bearing on health status and requires no medical/surgical intervention (eg, lack of housing, economic inadequacy, care-giver respite, family circumstances, administrative)

7.1.2 Nonserious Adverse Events

All AEs that are not classified as serious.

Approved v 7.0 930029550 5.0

7.2 Assignment of Adverse Event Intensity and Relationship to Investigational Product

The following categories and definitions of intensity as determined by a physician should be used for all BMS clinical study AEs:

- Mild (Grade 1) - Awareness of event but easily tolerated
- Moderate (Grade 2) - Discomfort enough to cause some interference with usual activity
- Severe (Grade 3) - Inability to carry out usual activity
- Very Severe (Grade 4) - Debilitating, significantly incapacitates subject despite symptomatic therapy.

The following categories and definitions of causal relationship to investigational product as determined by a physician should be used for all BMS clinical study AEs:

- Related: There is a reasonable causal relationship to investigational product administration and the AE
- Not related: There is not a reasonable causal relationship to investigational product administration and the AE.

The expression "reasonable causal relationship" is meant to convey in general that there are facts (eg, evidence such as de-challenge/re-challenge) or other arguments to suggest a positive causal relationship.

7.3 Collection and Reporting

Adverse events can be spontaneously reported or elicited during open-ended questioning, examination, or evaluation of a subject. (In order to prevent reporting bias, subjects should not be questioned regarding the specific occurrence of one or more AEs.)

If known, the diagnosis of the underlying illness or disorder should be recorded, rather than its individual symptoms. The following information should be captured for all AEs: onset, duration, intensity, seriousness, relationship to investigational product, action taken, and treatment required. If treatment for the AE was administered, it should be

Approved v7.0 930029550 5.0

recorded on the appropriate CRF page. The investigator shall supply the sponsor and Ethics Committee with any additional requested information, notably for reported deaths of subjects.

Completion of supplemental CRFs may be requested for AEs and/or laboratory abnormalities that are reported/identified during the course of the study.

7.3.1 Serious Adverse Events

Following the subject's written consent to participate in the study, all SAEs must be collected, including those thought to be associated with protocol-specified procedures. All SAEs must be collected that occur within 30 days of discontinuation of dosing of the investigational product. If applicable, SAEs must be collected that relate to any later protocol-specified procedure (eg, a follow-up skin biopsy). The investigator should notify BMS of any SAE occurring after this time period that is believed to be related to the investigational product or protocol-specified procedure.

Serious adverse events, whether related or unrelated to investigational product, must be recorded on the SAE page of the CRF and reported within 24 hours to BMS (or designee) to comply with regulatory requirements. An SAE report should be completed for any event where doubt exists regarding its status of seriousness.

All SAEs must be reported within 24 hours by confirmed facsimile transmission (fax) or scanned and reported via electronic mail. If only limited information is initially available, follow-up reports are required. (Note: Follow-up SAE reports should include the same investigator term(s) initially reported.) In selected circumstances, the protocol may specify conditions that require additional telephone reporting. The SAE electronic CRF in the electronic data capture tool should not be used.

If the investigator believes that an SAE is not related to the investigational product, but is potentially related to the conditions of the study (such as withdrawal of previous therapy, or a complication of a study procedure), the relationship should be specified in the narrative section of the SAE page of the CRF.

If an ongoing SAE changes in its intensity or relationship to the investigational product, a follow-up SAE report should be sent within 24 hours to the sponsor. As follow-up

Approved v7.0 930029550 5.0

information becomes available it should be sent within 24 hours using the same procedure used for transmitting the initial SAE report. All SAEs should be followed to resolution or stabilization.

SAE Email Address: Worldwide.Safety@BMS.com

SAE FACSIMILE TRANSMISSION:

For US Sites: Central Facsimile Station:
(609) 818-3804

SAE TELEPHONE CONTACT

Name: Ramesh Pappu, MD
Office: 609-252-4654
24 Hour: USA 1-866-470-2267
International 1-908-904-5520

7.3.2 Handling of Expedited Safety Reports

In accordance with local regulations, BMS will notify investigators of all SAEs that are suspected (related to the investigational product) and unexpected (ie, not previously described in the Investigator Brochure). In the European Union (EU), an event meeting these criteria is termed a Suspected, Unexpected Serious Adverse Reaction (SUSAR). Investigator notification of these events will be in the form of an expedited safety report (ESR).

Other important findings which may be reported by the sponsor as an ESR include: increased frequency of a clinically significant expected SAE, an SAE considered associated with study procedures that could modify the conduct of the study, lack of efficacy that poses significant hazard to study subjects, clinically significant safety finding from a nonclinical (eg, animal) study, important safety recommendations from a study data monitoring committee, or sponsor decision to end or temporarily halt a clinical study for safety reasons.

Upon receiving an ESR from BMS, the investigator must review and retain the ESR with the Investigator Brochure. Where required by local regulations or when there is a central

Approved v 7.0 930029550 5.0

IRB/IEC for the study, the sponsor will submit the ESR to the appropriate IRB/IEC. The investigator and IRB/IEC will determine if the informed consent requires revision. The investigator should also comply with the IRB/IEC procedures for reporting any other safety information.

In addition, suspected serious adverse reactions (whether expected or unexpected) shall be reported by BMS to the relevant competent health authorities in all concerned countries according to local regulations (either as expedited and/or in aggregate reports).

7.3.3 Nonserious Adverse Events

The collection of nonserious AE information should begin at initiation of investigational product. Nonserious AE information should also be collected from the start of a placebo lead-in period or other observational period intended to establish a baseline status for the subjects.

If an ongoing nonserious AE worsens in its intensity or its relationship to the investigational product changes, a new nonserious AE entry for the event should be completed. Nonserious AEs should be followed to resolution or stabilization, or reported as SAEs if they become serious (see Section 7.3.1). Follow-up is also required for nonserious AEs that cause interruption or discontinuation of investigational product, or those that are present at the end of study participation. Subjects with nonserious AEs at study completion should receive post-treatment follow-up as appropriate.

All identified nonserious AEs must be recorded and described on the appropriate nonserious AE page of the CRF (paper or electronic).

7.4 Laboratory Test Abnormalities

All laboratory test values captured as part of the study should be recorded on the appropriate laboratory test results pages of the CRF, or be submitted electronically from a central laboratory. In addition, the following laboratory abnormalities should also be captured on the nonserious AE CRF page (paper or electronic) or SAE paper CRF page as appropriate:

Approved v7.0 930029550 5.0

- Any laboratory test result that is clinically significant or meets the definition of an SAE
- Any laboratory abnormality that required the subject to have the investigational product discontinued or interrupted
- Any laboratory abnormality that required the subject to receive specific corrective therapy.

It is expected that wherever possible, the clinical, rather than the laboratory term would be used by the reporting investigator (eg, anemia versus low hemoglobin value).

7.5 Overdose

All occurrences of overdose must be reported as an SAE (see Section 7.3.1 for reporting details).

7.6 Pregnancy

Sexually active WOCBP must use an effective method of birth control during the course of the study, in a manner such that risk of failure is minimized (See Section 4.2.1 for the definition of WOCBP).

Before enrolling WOCBP in this clinical study, investigators must review the sponsor-provided information about study participation for WOCBP. The topics include the following:

- General Information
- Informed Consent Form
- Pregnancy Prevention Information Sheet
- Drug Interactions with Hormonal Contraceptives
- Contraceptives in Current Use
- Guidelines for the Follow-up of a Reported Pregnancy.

Prior to study enrollment, WOCBP must be advised of the importance of avoiding pregnancy during study participation and the potential risk factors for an unintentional pregnancy. The subject must sign an informed consent form documenting this discussion.

Approved v7.0 930029550 5.0

7.6.1 Requirements for Pregnancy Testing

All WOCBP MUST have a **negative** pregnancy test within 0 to 48 hours as specified in Section 6.1 **prior** to receiving the investigational product. The minimum sensitivity of the pregnancy test must be 25 IU/L or equivalent units of HCG. If the pregnancy test is positive, the subject must not receive the investigational product and must not continue in the study.

Pregnancy testing must also be performed throughout the study as specified in Section 6.1 (see flow chart/time and events schedule) including the interim months in which the subjects are not scheduled for a visit. At the interim visits, the WOCBP subjects will be provided with pregnancy test kits, to be used monthly and instructed to contact their study office to report the results of the pregnancy test. The results of all pregnancy tests (positive or negative) recorded on the CRF or transferred electronically.

In addition, all WOCBP should be instructed to contact the investigator immediately if they suspect they might be pregnant (eg, missed or late menstrual period) at any time during study participation.

7.6.2 Reporting of Pregnancy

If, following initiation of the investigational product, it is subsequently discovered that a study subject is pregnant or may have been pregnant at the time of investigational product exposure, including during at least 6 half-lives after product administration, the investigational product will be permanently discontinued in an appropriate manner (eg, dose tapering if necessary for subject safety). The investigator must immediately notify the BMS medical monitor of this event, record the pregnancy on the Pregnancy Surveillance Form (not an SAE form). Initial information on a pregnancy must be reported immediately to BMS and the outcome information provided once the outcome is known. Completed Pregnancy Surveillance Forms must be forwarded to BMS according to SAE reporting procedures described in Section 7.3.1.

Protocol-required procedures for study discontinuation and follow-up must be performed on the subject unless contraindicated by pregnancy (eg, x-ray studies). Other appropriate pregnancy follow-up procedures should be considered if indicated. Follow-up

Approved v7.0 930029550 5.0

information regarding the course of the pregnancy, including perinatal and neonatal outcome must be reported on the Pregnancy Surveillance Form.

Any pregnancy that occurs in a female partner of a male study participant should be reported to the Sponsor. Information on this pregnancy will be collected on the Pregnancy Surveillance Form.

7.7 Other Safety Considerations

Any significant worsening noted during interim or final physical examinations, electrocardiograms, x-rays, and any other potential safety assessments, whether or not these procedures are required by the protocol, should also be recorded on the appropriate nonserious AE page of the CRF (paper or electronic) or SAE paper CRF page.

8 STATISTICAL CONSIDERATIONS

8.1 Sample Size Determination

The study will include 30 patients. Although the sample size is not driven by hypothesis testing, given incidence rates in the table below, a sample size of 30 provides 45% probability of observing at least one occurrence of drug-related death, and 79% probability of observing at least one occurrence of discontinuation due to pleural effusion, and 96% probability of observing at least one occurrence of discontinuation due to pericardial effusion. The following table shows the cumulative probability of observing the incidences of the safety endpoint.

Table 8.1: Cumulative Probability of Endpoints

Endpoint (Incidence rate)	P (X > 0)[a]	P (X > 1)	P (X > 2)	P (X > 3)	P (X > 4)	P (X > 5)	P (X > 6)
Death (2%)	45%	12%	2%	0.3%	---	---	---
Pleural Effusion (5%)	79%	45%	19%	6%	2%	---	---
Peripheral edema (< 5%)	79%	45%	19%	6%	2%	---	---
Pericardial Effusion (10%)	96%	82%	59%	35%	18%	7%	3%

[a] P(X > 0) is a probability of having at least one incidence.

Approved v7.0 930029550 5.0

8.2 Populations for Analyses

There are 3 subject populations for purposes of statistical analyses in this study:

- Enrolled subjects: All subjects who signed an informed consent
- Treated subjects: All subjects who received at least one dose of dasatinib and will be use for safety analyses
- Response-evaluable subjects: All treated subjects who have at least one post-baseline assessment of FVC. The all response-evaluable subjects will be used for efficacy analyses.

8.3 Endpoint Definitions

The primary safety endpoints include the following:

- Incidence of drug-related death
- Incidence of discontinuation related to significant drug-related pericardial effusion
- Incidence of discontinuation related to significant drug-related pleural effusion
- Incidence of discontinuation related to significant drug-related peripheral edema
- Incidence of discontinuation related to significant drug-related bone marrow suppression
- Incidence of discontinuation related to significant worsening of underlying scleroderma
- Incidence of discontinuation related to other unforeseen toxicities of dasatinib in this population.

8.4 Analyses

A complete analysis will be detailed in the Statistical Analysis Plan (SAP) and Data Presentation Plan (DPP). Database lock will occur once all subjects complete the first 6 months of treatment. A clinical study report will be generated at that time. An additional data base lock and analysis will occur once all subjects have completed the additional 18 months of treatment.

Approved v7.0 930029550 5.0

8.4.1 Demographics and Baseline Characteristics

Demographic and baseline characteristics will be summarized using summary statistics and frequency tables using the treated subjects dataset.

8.4.2 Safety Analyses

All treated subjects population will be used for safety analyses. Each primary endpoint incidence rate is calculated as the proportion of subjects with a discontinuation reason due to the primary endpoint divided by all treated subjects.

The frequency and percentage of each primary safety endpoints along with an exact 95% two-sided binomial confidence interval will be provided without adjustment for multiple comparisons.

The frequency of occurrence of adverse events (AEs) and laboratory abnormalities will be provided. Tabulations of AEs considered at least possibly-related to study drug and of AEs regardless of relationship will be provided separately.

Exploratory analyses will be performed of the relationship between the incidence of drug-related adverse events and extent of prior treatment, pre-study AEs, study drug dose and treatment duration.

8.4.3 Efficacy Analyses

The all response-evaluable subjects population will be used for efficacy analyses. For continuous measurements, such as FVC, mean change from baseline to each scheduled study visit and its standard error will be estimated. The mean change from baseline values along with individual change from baseline values at each study visit will be presented in a plot. Individual best response values, which are defined as the largest percent change from baseline to any study visit will be presented.

For categorical measurement, such as scleroderma skin severity score, frequency and percentage of each category will be presented.

Approved v7.0 930029550 5.0

8.4.4 Pharmacokinetic Analyses

Not Applicable

8.4.5 Pharmacodynamic Analyses

Not Applicable

8.4.6 Pharmacogenomic Analyses

Not Applicable

8.4.7 Outcomes Research Analyses

Not Applicable

8.4.8 Other Analyses

Not Applicable

8.5 Interim Analyses

Not Applicable

9 ADMINISTRATIVE SECTION

9.1 Compliance

9.1.1 Compliance with the Protocol and Protocol Revisions

The study shall be conducted as described in this approved protocol. All revisions to the protocol must be discussed with, and be prepared by, BMS. The investigator should not implement any deviation or change to the protocol without prior review and documented approval/favorable opinion from the IRB/IEC of an amendment, except where necessary to eliminate an immediate hazard(s) to study subjects. Any significant deviation must be documented in the CRF.

Approved v7.0 930029550 5.0

If a deviation or change to a protocol is implemented to eliminate an immediate hazard(s) prior to obtaining IRB/IEC approval/favorable opinion, as soon as possible the deviation or change will be submitted to:

- IRB/IEC for review and approval/favorable opinion
- Bristol-Myers Squibb
- Regulatory Authority(ies), if required by local regulations.

Documentation of approval signed by the chairperson or designee of the IRB(s)/IEC(s) must be sent to BMS.

If an amendment substantially alters the study design or increases the potential risk to the subject: (1) the consent form must be revised and submitted to the IRB(s)/IEC(s) for review and approval/favorable opinion; (2) the revised form must be used to obtain consent from subjects currently enrolled in the study if they are affected by the amendment; and (3) the new form must be used to obtain consent from new subjects prior to enrollment.

If the revision is an administrative letter, investigators must inform their IRB(s)/IEC(s).

9.1.2 Monitoring

Representatives of BMS must be allowed to visit all study site locations periodically to assess the data quality and study integrity. On site they will review study records and directly compare them with source documents, discuss the conduct of the study with the investigator, and verify that the facilities remain acceptable.

In addition, the study may be evaluated by BMS internal auditors and government inspectors who must be allowed access to CRFs, source documents, other study files, and study facilities. BMS audit reports will be kept confidential.

THE INVESTIGATOR MUST NOTIFY BMS PROMPTLY OF ANY INSPECTIONS SCHEDULED BY REGULATORY AUTHORITIES, AND PROMPTLY FORWARD COPIES OF INSPECTION REPORTS TO BMS.

Approved v7.0 930029550 5.0

9.1.3 Investigational Site Training

Bristol-Myers Squibb will provide quality investigational staff training prior to study initiation. Training topics will include but are not limited to: GCP, AE reporting, study details and procedure, study documentation, informed consent, and enrollment of WOCBP.

For sites using the BMS electronic data capture tool, each individual making entries and/or corrections on electronic CRFs must meet BMS training requirements and must only access the BMS electronic data capture tool using the unique user account provided by the sponsor. User accounts are not to be shared or reassigned to other individuals.

For electronic CRFs, corrections are made through the BMS electronic data capture tool that generates an automated audit trail including date and timestamp, full name of the person making the correction and original entry. The system also prompts the user to document reason for change that is also maintained in the audit trail.

Each individual electronically signing electronic CRFs must meet BMS training requirements and must only access the BMS electronic data capture tool using the unique user account provided by the sponsor. User accounts are not to be shared or reassigned to other individuals.

9.2 Records Retention

The investigator must retain investigational product disposition records, copies of CRFs (or electronic files), and source documents for the maximum period required by applicable regulations and guidelines, or institution procedures, or for the period specified by the sponsor, whichever is longer. The investigator must contact BMS prior to destroying any records associated with the study.

BMS will notify the investigator when the study records are no longer needed.

If the investigator withdraws from the study (eg, relocation, retirement), the records shall be transferred to a mutually agreed upon designee (eg, another investigator, IRB). Notice of such transfer will be given in writing to BMS.

Approved v7.0 930029550 5.0

9.2.1 Case Report Forms

An investigator is required to prepare and maintain adequate and accurate case histories designed to record all observations and other data pertinent to the investigation on each individual treated or entered as a control in the investigation. Data reported on the CRF that are derived from source documents must be consistent with the source documents or the discrepancies must be explained.

For sites using the BMS electronic data capture tool, electronic CRFs will be prepared for all data collection fields except for fields specific to SAEs and pregnancy, which will be reported on the Pregnancy Surveillance Form. Paper CRFs must be completed legibly in ink. Subjects are to be identified by birth date and subject number, if applicable. All requested information must be entered on the CRF in the spaces provided. If an item is not available or is not applicable, it must be documented as such; do not leave a space blank. Spaces may be left blank only in those circumstances permitted by study-specific CRF completion guidelines provided by the sponsor. Electronic data transfer is acceptable.

The confidentiality of records that could identify subjects must be protected, respecting the privacy and confidentiality rules in accordance with the applicable regulatory requirement(s).

The investigator will maintain a signature sheet to document signatures and initials of all persons authorized to make entries and/or corrections on CRFs.

For paper CRFs, a correction must be made by striking through the incorrect entry with a single line and entering the correct information adjacent to the incorrect entry. The correction must be dated, initialed and explained (if necessary) by the person making the correction and must not obscure the original entry.

The completed CRF, including any paper SAE/pregnancy CRFs, must be promptly reviewed, signed, and dated by a qualified physician who is an investigator or subinvestigator. For electronic CRFs, review and approval/signature is completed electronically through the BMS electronic data capture tool. The investigator must retain a copy of the CRFs including records of the changes and corrections.

Approved v7.0 930029550 5.0

9.2.2 Investigational Product Records

It is the responsibility of the investigator to ensure that a current record of investigational product disposition is maintained at each study site where investigational product is inventoried and disposed. Records or logs must comply with applicable regulations and guidelines and should include:

- amount received and placed in storage area
- amount currently in storage area
- label ID number or batch number and use date or expiry date
- dates and initials of person responsible for each investigational product inventory entry/movement
- amount dispensed to and returned by each subject, including unique subject identifiers
- amount transferred to another area/site for dispensing or storage
- non-study disposition (eg, lost, wasted, broken)
- amount returned to the sponsor
- amount destroyed at study site, if applicable
- retain samples sent to third party for bioavailability/bioequivalence, if applicable.

The sponsor will provide forms to facilitate inventory control if the staff at the investigational site does not have an established system that meets these requirements.

9.3 Return and Destruction of Investigational Product

9.3.1 Return of Investigational Product

Upon completion or termination of the study, all unused and/or partially used investigational product must be returned to BMS, if not authorized by BMS to be destroyed at the site.

All investigational product returned to BMS must be accompanied by the appropriate documentation and be clearly identified by protocol number and study site number on the outermost shipping container. Returned supplies should be in the original containers (eg, patient kits that have clinical labels attached). Empty containers should not be returned to

Approved v7.0 930029550 5.0

BMS. It is the investigator's responsibility to arrange for disposal of all empty containers, provided that procedures for proper disposal have been established according to applicable federal, state, local, and institutional guidelines and procedures, and provided that appropriate records of disposal are kept. The return of unused investigational product(s) should be arranged by the responsible Study Monitor.

9.3.2 Destruction of Investigational Product

If investigational products are to be destroyed on site, it is the investigator's responsibility to ensure that arrangements have been made for the disposal, written authorization has been granted by BMS, procedures for proper disposal have been established according to applicable regulation and guidelines and institutional procedures, and appropriate records of the disposal have been documented. The unused investigational products can only be destroyed after being inspected and reconciled by the responsible BMS Study Monitor.

9.4 Publications

The data collected during this study are confidential and proprietary to the sponsor. Any publications or abstracts arising from this study require approval by the sponsor prior to publication or presentation and must adhere to the sponsor's publication requirements as set forth in the approved clinical trial agreement (CTA). All draft publications, including abstracts or detailed summaries of any proposed presentations, must be submitted to the sponsor at the earliest practicable time for review, but at any event not less than 30 days before submission or presentation unless otherwise set forth in the CTA. Sponsor shall have the right to delete any confidential or proprietary information contained in any proposed presentation or abstract and may delay publication for up to 60 days for purposes of filing a patent application.

Approved v7.0 930029550 5.0

10 GLOSSARY OF TERMS

Term	Definition
Adverse Reaction	An adverse event that is considered by either the investigator or the sponsor as certainly, probably, or possibly to the investigational product
Expedited Safety Report	Rapid notification to investigators of all SAEs that are suspected (related to the investigational product) and unexpected (ie, not previously described in the Investigator Brochure), or that could be associated with the study procedures.
SUSAR	Suspected, Unexpected, Serious Adverse Reaction as termed by the European Clinical Trial Directive (2001/20/EC).
Unexpected Adverse Reaction	An adverse reaction, the nature or severity of which is not consistent with the applicable product information (eg, Investigator Brochure for an unapproved investigational product)

Approved v7.0 930029550 5.0

11 LIST OF ABBREVIATIONS

Abl	Abelson leukemia oncogene cellular homolog; human form found on chromosome 9
ACE	Angiotensin concerting enzyme
ACR	American College of Rheumatology
AE	Adverse event
APRIL	A Proliferation-Inducing Ligand [APRIL])
ALT	Alanine aminotransferase
AST	Aspartate aminotransferase
AUC	Area under the curve
BAL	Bronchoalveolar lavage
Bcr	Breakpoint cluster region- the area of human chromosome 22 that fuses with Abl (on chromosome 9) to form Bcr-ABL: the “Philadelphia chromosome”
Bcr-Abl	Fusion protein of Bcr (chromosome 22) and Abl (chromosome 9), found in chronic myeloid leukemia- the “Philadelphia chromosome” is the new fusion chromosome 22
BID	Two times a day
BMS	Bristol-Myers Squibb
CHF	Congestive Heart Failure
CMax	Maximum cancentration
CML	Chronic myeloid leukemia
CTX	Cyclophosphamide
CXR	Chest Xray
DDR1	Discoidin domain receptor tyrosine kinase 1
D_LCO	Diffusing capacity for carbon monoxide
ECM	Extracellular matrix
FEV1	Forced expiratory volume in the first 1 second of expiration
FSH	Follicle Stimulating Hormone
FVC	Forced vital capacity
GCP	Good clinical practice
H_2	Histamine Receptor
Hgb	Hemoglobin
HRCT	High resolution computer tomography
HRT	Hormone Replacement Therapy

Approved v7.0 930029550 5.0

ICH	International Conference on Harmonization of Technical Requirements for Registration of Pharmaceuticals for Human Use
IEC	Independent Ethics Committee
IL	Interleukin
IRB	Institutional Review Board
MCP-1	Monocyte chemoattractant protein-1
MI	Myocardial Infarction
MIP-1α	Macrophage inflammatory protein-1α
MST	Medical Surveillance Team
MRT	Medical Review Team
PDGF	Platelet-derived growth factor
PFT	Pulmonary function tests
PMN	Polymorphonuclear Neutrophilic Leukocyte
PPI	Protein Pump Inhibitor
PO	By mouth
QD	Once a day
QTC	QT interval corrected for rate
Src	Rous sarcoma oncogene cellular homolog
TLC	Total Lung Capacity
TGFβ	Transforming growth factor β
TNFα	Tumor necrosis factor α
WBC	White Blood Cell
WOCBP	Women of Child Bearing Potential

Approved v7.0 930029550 5.0

12 REFERENCES

1 Wigley FM. Scleroderma (systemic sclerosis) In Cecil Textbook of Medicine 22nd Edition. Editors Goldman L and Ausiello D. Saunders, Philadelphia, PA 2004 1670-1677.

2 Boin F, Rosen A. Autoimmunity in systemic sclerosis: current concepts. Curr Rheumatol Rep. 2007 May;9(2):165-72.

3 Mcnearney TA, Reveille JD, Fischbach M Pulmonary involvement in systemic sclerosis: Associations with genetic, serologic, sociodemographic, and behavioral factors Arthritis Rheum 2007;56(2):318-326.

4 Renzoni EA. Interstitial lung disease in systemic sclerosis. Monaldi Arch Chest Dis 2007;67(4):217-228.

5 Tashkin DP, Elashoff R, Clements PJ, et al. Cyclophosphamide versus placebo in scleroderma lung disease. New Engl J Med 2006 (25):2655-2666.

6 Tashkin DP, Elashoff R, Clements PJ, et al. Effects of 1-year treatment with cyclophosphamide on outcomes at 2 years in scleroderma lung disease. Am J Respir Crit Care Med 2007;176:1026-1034.

7 Strange C, Bolser MB, Roth MD, et al. Bronchoalveolar lavage and response to cyclophosphamide in scleroderma interstitial lung disease. Am J Respir Crit Care Med 2007;176:952-953.

8 Khanna D, Yan X, Tashkin DP, et al. Impact of oral cyclophosphamide on health-related quality of life in patients with active scleroderma lung disease: Results from the scleroderma lung study. Arthritis Rheum 2007;56(5):1676-1684.

9 Wells AU, Latsi P, McCune WJ. Daily cyclophosphamide for scleroderma. Am J Respir Crit Care Med 2007;176:952-953.

Approved v7.0 930029550 5.0

10 Hoyles RK, Ellis RW, Wellsbury J, et al. A multicenter, prospective, randomized, double-blind, placebo-controlled trial of corticosteroids and intravenous cyclophosphamide followed by oral azathioprine for the treatment of pulmonary fibrosis in scleroderma. Arthritis Rheum 2006;54(12):3962-3970.

11 Basu D, Reveille JD. Anti-scl70. Autoimmunity 2005 ;38(1) :65-72.

12 Sakkas LI, Chikanza IC, Platsoucas CD. Mechanisms of Disease: the role of immune cells in the pathogenesis of systemic sclerosis. Nat Clin Pract Rheumatol. 2006 Dec;2(12):679-85.

13 Abdollah A. Li M, Ping G, et al. Inhibition of platelet-derived growth factor signaling attenuates pulmonary fibrosis. J Exp Med 2005 Mar 21; 201 (6):925-35.

14 Bueso-Ramos CE, Cortes J, Talpaz M, et al. Imatinib mesylate therapy reduces bone marrow fibrosis in patients with chronic myelogenous leukemia. Cancer 2004 Jul 15; 101(2):332-6.

15 Daniels CE, Wilkes MC, Edens M, et al. Imatinib mesylate inhibits the profibrogenic activity of TGF beta and prevents bleomycin-mediated lung fibrosis. J Clin Invest 2004 Nov; 114(9):1308-16.

16 Distler JHW, Jungel A, Huber LC, et al. Imatinib mesylate reduces production of extracellular matrix and prevents development of experimental dermal fibrosis. Arthritis Rheum 2007 Jan; 56(1):311-22.

17 Paniagua RT, Robinson WH. Imatinib for the treatment of rheumatic diseases. Nature Clin Practice Rheum 2007 Apr; 3(4):190-1.

18 Kyttaris VC, Tsokos GC. Syk kinase as a treatment target for therapy in autoimmune diseases. Clinical Immunology 2007;124(2):235-7.

Approved v7.0 930029550 5.0

19 Schade AE, Schieven GL, Townsend R, et al. Dasatinib, a small molecule protein kinase inhibitor, Inhibits T-cell activation and proliferation. Blood. In press

20 Weichsel R, Dix C, Wooldridge L, Clement M, Fenton-May A, Sewell AK, Zezula J, Greiner E, Gostick E, Price DA, Einsele H, Seggewiss R. Profound inhibition of antigen-specific T-cell effector functions by dasatinib. Clin Cancer Res. 2008 Apr 15;14(8):2484-91.

21 Blake S, Hughes TP, Mayrhofer G, Lyons AB. The Src/ABL kinase inhibitor dasatinib (BMS-354825) inhibits function of normal human T-lymphocytes in vitro.Clin Immunol. 2008 Apr 3. [Epub ahead of print]

22 Schade AE, Schieven GL, Townsend R, Jankowska AM, Susulic V, Zhang R, Szpurka H, Maciejewski JP. Dasatinib, a small-molecule protein tyrosine kinase inhibitor, inhibits T-cell activation and proliferation. Blood. 2008 Feb 1;111(3):1366-77.

23 Shah NP, Lee FY, Luo R, Jiang Y, Donker M, Akin C. Dasatinib (BMS-354825) inhibits KITD816V, an imatinib-resistant activating mutation that triggers neoplastic growth in most patients with systemic mastocytosis. Blood. 2006 Jul 1;108(1):286-91.

24 Hantschel O, Rix U, Schmidt U, Bürckstümmer T, Kneidinger M, Schütze G, Colinge J, Bennett KL, Ellmeier W, Valent P, Superti-Furga G. The Btk tyrosine kinase is a major target of the Bcr-Abl inhibitor dasatinib. Proc Natl Acad Sci U S A. 2007 Aug 14;104(33):13283-8.

25 Akhmetshina A, Dees C, Pileckyte M, Maurer B, Axmann R, Jüngel A, Zwerina J, Gay S, Schett G, Distler O, Distler JH. Dual inhibition of c-abl and PDGF receptor signaling by dasatinib and nilotinib for the treatment of dermal fibrosis. FASEB J. 2008 Mar 7. [Epub ahead of print]

Approved v7.0 930029550 5.0

[26] Luo FR, Yang Z, Camuso A, Smykla R, McGlinchey K, Fager K, Flefleh C, Castaneda S, Inigo I, Kan D, Wen M-L, Kramer R, Blackwood-Chirchir A, Lee FY. Dasatinib (BMS-354825) pharmacokinetics and pharmacodynamic biomarkers in animal models predict optimal clinical exposure. Clin Cancer Res 2006;12(23) December 1:7180-6.

[27] Weichsel R, Dix C, Wooldridge L, Clement M, Fenton-May A, Sewell AK, Zezula J, Greiner E, Gostick E, Price DA, Einsele H, Seggewiss R. Profound inhibition of antigen-specific T-cell effector functions by dasatinib. Clin Cancer Res 2008;14(8) April 15:2484-91.

[28] Varga J and Abraham D. Systemic sclerosis: a prototypic multisystem fibrotic disorder. J Clin Invest 2007; 117(3):557-67.

[29] Karaman MW, Herrgard S, Treiber DK, Gallant P, Atteridge CE, Campbell BT, Chan KW, Ciceri P, Davis MI, Edeen PT, Faraoni R, Floyd M, Hunt JP, Lockhart DJ, Milanov ZV, Morrison MJ, Pallares G, Patel HK, Pritchard S, Wodicka LM, Zarrinkar PP. A quantitative analysis of kinase inhibitor selectivity. Nat Biotechnol. 2008 Jan;26(1):127-32.

Approved v7.0 930029550 5.0

APPENDIX 1 ADDITIONAL ETHICAL CONSIDERATIONS

1 INFORMED CONSENT PROCEDURES

BMS will provide the investigator with an appropriate (ie, Global or Local) sample informed consent form which will include all elements required by ICH, GCP and applicable regulatory requirements. The sample informed consent form will adhere to the ethical principles that have their origin in the Declaration of Helsinki. If the investigator makes changes to the informed consent form sample, BMS will ensure all required elements and local regulatory and legal requirements are met.

The consent form must also include a statement that BMS and regulatory authorities have direct access to subject records. Prior to the beginning of the study, the investigator must have the IRB/IEC's written approval/favorable opinion of the written informed consent form and any other information to be provided to the subjects.

The investigator must provide the subject, or, in those situations where consent cannot be given by subjects, their legally acceptable representative with a copy of the consent form and written information about the study in the language in which the subject is most proficient. The language must be non-technical and easily understood. The investigator should allow time necessary for subject or subject's legally acceptable representative to inquire about the details of the study, then informed consent must be signed and personally dated by the subject or the subject's legally acceptable representative and by the person who conducted the informed consent discussion. The subject or legally acceptable representative should receive a copy of the signed informed consent and any other written information provided to study subjects prior to subject's participation in the study.

1.1 Subjects Unable to Give Written Informed Consent

1.1.1 Minors

For minors, according to local legislation, one or both parents or a legally acceptable representative must be informed of the study procedures and must sign the informed consent form approved for the study prior to clinical study participation. (In the event that

Approved v7.0 930029550 5.0

the parents or legal guardians are unable to read, then an impartial witness should be present during the entire informed consent discussion). Whenever feasible, minors who are judged to be of an age of reason must also give their written assent by signing and dating the completed informed consent. All local laws, rules and regulations regarding informed consent of minors must be followed.

1.1.2 Subjects Experiencing Acute Events or Emergencies

A legally acceptable representative or legal guardian must provide informed consent when consent of the subject is not possible prior to clinical study participation, eg, for subjects experiencing an acute medical event such as myocardial infarction or stroke. Informed consent of the subject must additionally be obtained if they become capable of making and communicating their informed consent during the clinical study. All local laws, rules and regulations regarding informed consent of adult subjects incapable of giving informed consent must be followed.

1.1.3 Mentally Impaired or Incapacitated Subjects

Investigators (or whoever required by local regulations) should determine whether or not a mentally impaired or incapacitated subject is capable of giving informed consent and should sign a statement to that effect. If the subject is deemed mentally competent to give informed consent, the investigator should follow standard procedures. If the subject is deemed not to be mentally competent to give informed consent, a fully informed legal guardian or legally acceptable representative can be asked to give consent for, or on behalf of, the subject. All local laws, rules and regulations regarding informed consent of mentally impaired or incapacitated subjects must be followed.

Patients who are involuntarily hospitalized because of mental illness must not be enrolled in clinical studies

1.1.4 Other Circumstances

Subjects who are imprisoned or involuntarily detained for treatment of either a psychiatric or physical (eg, infectious disease) illness must not be enrolled in clinical studies.

Approved v7.0 930029550 5.0

In circumstances where a subject's only access to treatment is through enrollment in a clinical study, eg, for subjects in developing countries with limited resources or for subjects with no marketed treatment options, the investigator must take special care to explain the potential risks and benefits associated with the study and ensure that the subject is giving informed consent.

When a subject may be in a dependent relationship with the investigator, a well-informed physician who is not engaged in the clinical study and is completely independent of the relationship between the subject and investigator should obtain the subject's informed consent.

1.1.5 Illiterate Subjects

If the subject, or, in those situations where consent cannot be given by the subject, their legally acceptable representative is unable to read, a reliable and independent witness should be present during the entire informed consent discussion. The choice of the witness must not breach the subject's rights to confidentiality. A reliable independent witness is defined as one not affiliated with the institution or engaged in the investigation. A family member or acquaintance is an appropriate independent witness. After the subject or legally acceptable representative orally consents and has signed, if capable, the witness should sign and personally date the consent form attesting that the information is accurate and that the subject, or, in those situations where consent cannot be given by subjects, their legally acceptable representative has fully understood the content of the informed consent agreement and is giving true informed consent.

1.2 Update of Informed Consent

The informed consent and any other information provided to subjects, or, in those situations where consent cannot be given by subjects, the subject's legally acceptable representative, should be revised whenever important new information becomes available that is relevant to the subject's consent, and should receive IRB/IEC approval/favorable opinion prior to use. The investigator, or a person designated by the investigator should fully inform the subject or the subject's legally acceptable representative of all pertinent aspects of the study and of any new information relevant to the subject's willingness to continue participation in the study. This communication should be documented.

Approved v7.0 930029550 5.0

During a subject's participation in the study, any updates to the consent form and any updates to the written information will be provided to the subject.

Approved v7.0 930029550 5.0

APPENDIX 2 CRITERIA FOR THE CLASSIFICATION OF SYSTEMIC SCLEROSIS (SCLERODERMA)

1980 Criteria for the Classification of Systemic Sclerosis

The American College of Rheumatology (former American Rheumatism Association - ARA) has defined criteria, that are 97% sensitive and 98% specific for systemic sclerosis (SSc) as follows:

Major criterion:

- Proximal diffuse (truncal) sclerosis (skin tightness, thickening, non-pitting induration)

Minor criteria:

- Sclerodactyly (only fingers and/or toes)
- Digital pitting scars or loss of substance of the digital finger pads (pulp loss)
- Bilateral basilar pulmonary fibrosis

The patient should fulfill the major criterion or two of the three minor criteria. Raynaud's phenomenon is observed in 90-98 % of SSc patients.

Approved v7.0 930029550 5.0

Subsets of Systemic Sclerosis

	**Diffuse**	**Limited**[a]
Skin involvement	Distal and proximal extremities, face, trunk	Distal to elbows, face
Raynaud's phenomenon	Onset within 1 year or at time of skin changes	May precede skin disease by years
Organ involvement	Pulmonary (interstitial fibrosis); renal (renovascular hypertensive crisis); gastrointestinal; cardiac	Gastrointestinal; pulmonary arterial hypertension after 10- 15 years of disease in < 10% of patients; biliary cirrhosis
Nail fold capillaries	Dilatation and dropout	Dilatation without significant dropout
Antinuclear antibodies	Anti-topoisomerase 1	Anticentromere

[a] Also referred to as CREST (calcinosis, Raynaud's, esophageal dysmotility, sclerodactyly, telangiectasia).

References: Subcommittee for Scleroderma Criteria of the American Rheumatism Association Diagnostic and Therapeutic Criteria Committee. Preliminary criteria for the classification of systemic sclerosis (scleroderma). Arthritis Rheum 1980; 23: 581-90. Medline

Approved v7.0 930029550 5.0

APPENDIX 3 SKIN SCLEROSIS SCORE

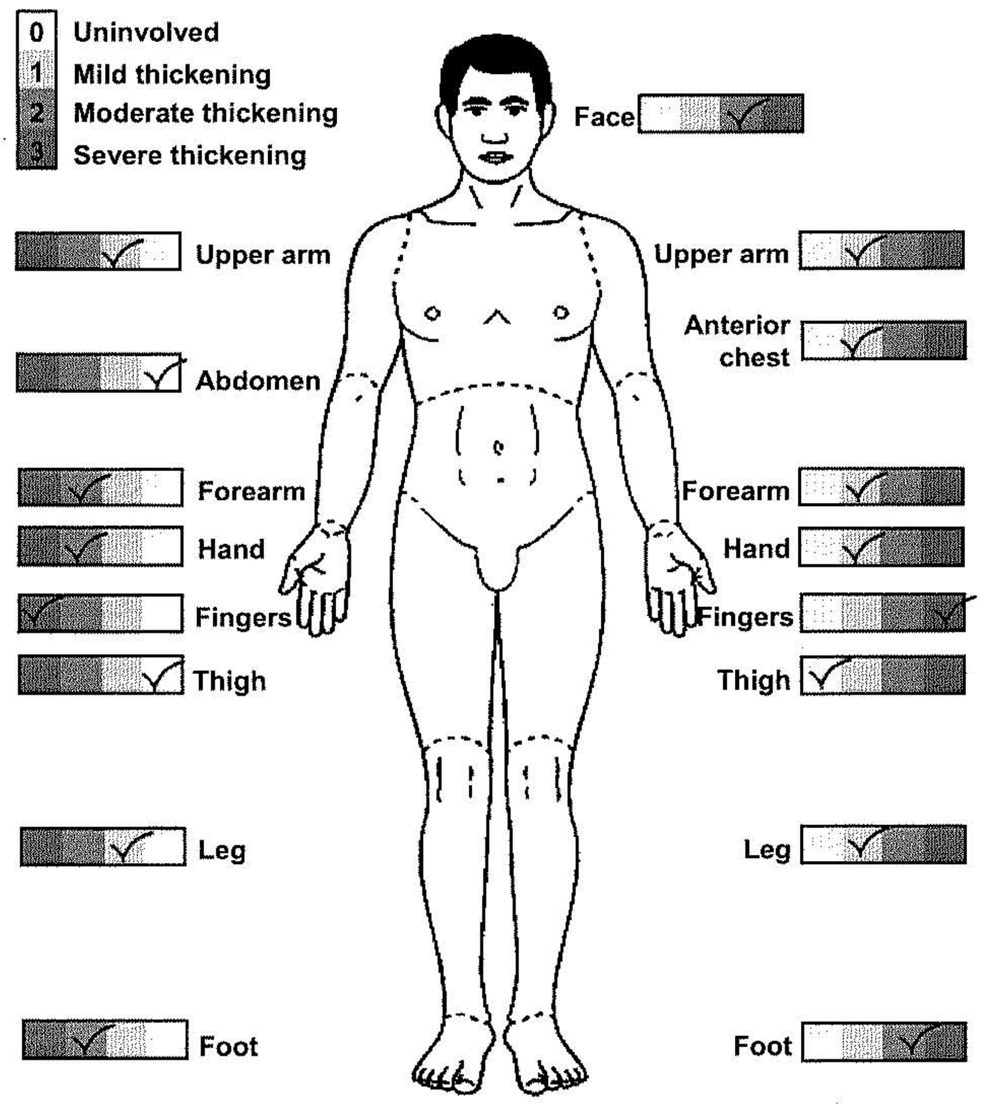

Approved v 7.0 930029550 5.0

Modified Rodnan Skin Score:

Face	3	
Neck	3	
Anterior chest	3	
Abdomen	3	
Back - upper	3	
Back - lower	3	
	**9** (18)	
Upper arm	3	3
Forearm	3	3
Hand	3	3
Fingers	3	3
Thigh	3	3
Leg	3	3
Foot	3	3
	**21**	**21**
**Maximum (17 site)**		**51**
**20 site**		**60**

Approved v7.0 930029550 5.0

APPENDIX 4 NEW YORK HEART ASSOCIATION (NYHA) CLASSIFICATION

New York Heart Association (NYHA) Classification:

A functional and therapeutic classification for prescription of physical activity for cardiac patients.

- Class I: patients with no limitation of activities; they suffer no symptoms from ordinary activities
- Class II: patients with slight, mild limitation of activity; they are comfortable with rest or with mild exertion
- Class III: patients with marked limitation of activity; they are comfortable only at rest
- Class IV: patients who should be at complete rest, confined to bed or chair; any physical activity brings on discomfort and symptoms occur at rest.

Approved v7.0 930029550 5.0

APPENDIX 5 COMMON CYP3A4 SUBSTRATES, INHIBITORS AND INDUCERS (NOT ALL INCLUSIVE)

SUBSTRATES

The following list describe medications which are common **CYP3A4 substrates**. This list should not be considered all inclusive. Consult individual drug labels for specific information on a compound's propensity for metabolism by CYP3A4.

Macrolide Antibiotics:
clarithromycin
erythromycin
NOT azithromycin

Anti-arrhythmics:
quinidine

Benzodiazepines:
alprazolam
diazepam
midazolam
triazolam

Immune Modulators:
cyclosporine
tacrolimus (FK506)

HIV Antivirals:
indinavir
nelfinavir
ritonavir
saquinavir

Antihistamines:
astemizole
chlorpheniramine
terfenidine

Approved v7.0 930029550 5.0

Calcium Channel Blockers:
amlodipine
diltiazem
felodipine
lercanidipine
nifedipine
nisoldipine
nitrendipine
verapamil

HMG CoA Reductase Inhibitors:
atorvastatin
cerivastatin
lovastatin
NOT pravastatin
simvastatin

Steroid 6beta-OH:
estradiol
hydrocortisone
progesterone
testosterone

Others:
alfentanyl
buspirone
cafergot
caffeine = > TMU
cocaine
dapsone
codeine-N-
demethylation
dextromethorphan
eplerenone
fentanyl
finasteride
gleevec
haloperidol (in part)
irinotecan
LAAM
lidocaine
methadone

Approved v7.0 930029550 5.0

odanestron
pimozide
propranolol
quinine
salmetrerol
sildenafil
sirolimus
tamoxifen
taxol
terfenadine
trazodone
vincristine
zaleplon
zolpidem

INHIBITORS

The following list describe medications and foods which are **common inhibitors of CYP3A4.** This list should not be considered all inclusive. Consult individual drug labels for specific information on a compound's propensity to inhibit CYP3A4.

HIV Antivirals:
delaviridine
indinavir
nelfinavir
ritonavir
saquinavir

Others:
amiodarone NOT azithromycin
cimetidine
ciprofloxacin
clarithromycin
diethyl-
dithiocarbamate
diltiazem
erythromycin
fluconazole

Approved v 7.0 930029550 5.0

fluvoxamine
gestodene
grapefruit juice
pomegranate juice
itraconazole
ketoconazole
mifepristone
nefazodone
norfloxacin
norfluoxetine
mibefradil
verapamil

INDUCERS

The following lists describe medications which are common **inducers of CYP3A4**. This list should not be considered all inclusive. Consult individual drug labels for specific information on a compound's propensity to induce CYP3A4.

HIV Antivirals:
efavirenz
nevirapine

Others:
barbiturates
carbamazepine
glucocorticoids
modafinil
phenobarbital
phenytoin
rifampin
St. John's wort
troglitazone
pioglitazone
rifabutin

APPENDIX 6 LABORATORY GUIDELINES: PATIENT STUDIES (REVISED AUGUST 13, 1999)

Laboratory test results, which meet these criteria, and the Investigator feels is clinically relevant should be described on the Adverse Event Form. Those which are judged to be SERIOUS events require the completion of a Serious Adverse Event Form.

[NOTE: LLN = lower limit of normal; ULN = upper limit of normal.]

albumin - <0.9xLLN or if pretreatment value is <LLN, <0.75x pretreatment

alkaline phosphatase - >2 x ULN; or if pretreatment >ULN, 3x pretreatment value

basophils (%) - >3% if 0-1% pretreatment, >3x pretreatment value if pretreatment >1%

bilirubin

a. direct - >1.5x ULN, or if pretreatment above ULN, >2x pretreatment

b. total - >2x upper limit of normal, or if pretreatment above ULN, >4 x pretreatment value

blasts - >0

blood urea nitrogen (BUN) - >2x pretreatment

calcium - <0.8x LLN or >1.2x ULN; <0.75x pretreatment if pretreatment below LLN, or >1.25x pretreatment if pretreatment above ULN; >ULN if <LLN pretreatment, or <LLN if >ULN pretreatment

chloride - <0.9x LLN or >1.1x ULN; or <0.9x pretreatment if pretreatment below LLN, or >1.1x pretreatment if pretreatment above ULN; >ULN if <LLN pretreatment, or <LLN if >ULN pretreatment

creatinine - >1.5x pretreatment value

Approved v7.0 930029550 5.0

eosinophils (%) - >3x pretreatment and > 8% if pretreatment normal; if pretreatment >ULN, >3 x pretreatment

erythrocytes - <0.75x pretreatment value

glucose - <0.8x LLN or >1.5x ULN; if pretreatment <LLN then <0.8x pretreatment; if pretreatment >ULN then >2x pretreatment; <LLN if >ULN pretreatment, or >ULN if <LLN pretreatment

hematocrit - <0.75x pretreatment

hemoglobin - >3 g/dL decrease from pretreatment value

lactic dehydrogenase (LDH) - >1.5x ULN; if pretreatment value is above ULN, >3x pretreatment value

leukocyte (WBC) count - <0.75 x LLN or >1.25x ULN; <0.8x pretreatment if pretreatment <LLN or >1.2x pretreatment if pretreatment >ULN; >ULN if pretreatment <LLN or <LLN if >ULN pretreatment

lymphocytes (%) - <0.5x LLN or >2.0 x ULN, or <0.5x pretreatment if below LLN pretreatment; >2.0x pretreatment if above ULN pretreatment. >ULN if <LLN pretreatment, or <LLN if >ULN pretreatment

monocytes (%) - >2x ULN; >2x pretreatment if pretreatment above ULN

neutrophil count (neutrophils+bands) - <0.67x pretreatment if pretreatment <1000/mm^3; otherwise, <1000/mm^3.

phosphate - <0.75 x LLN or >1.25x ULN; <0.67x pretreatment value if below LLN pretreatment, or >1.33x pretreatment if above ULN pretreatment; >ULN if <LLN pretreatment, or <LLN if >ULN pretreatment

platelet count - <0.67x LLN or >1.5 x ULN; if below normal pretreatment, <0.5x pretreatment value and <100,000/mm^3

Approved v7.0 930029550 5.0

potassium - <0.9x LLN or >1.1x ULN; <0.9x pretreatment if pretreatment below LLN, or >1.1x pretreatment if pretreatment above ULN; >ULN if <LLN pretreatment, or <LLN if >ULN pretreatment

protein, total - <0.9x LLN or >1.1x ULN; <0.9 x pretreatment if below LLN pretreatment, or >1.1x ULN if above ULN pretreatment; >ULN if <LLN pretreatment, or <LLN if >ULN pretreatment

SGOT and SGPT (ASAT and ALAT) - >3 x upper limit of normal; if pretreatment above ULN, >4x pretreatment value

sodium - <0.95x LLN or >1.05x ULN; <0.95x pretreatment if below LLN pretreatment, >1.05x pretreatment if above ULN pretreatment; >ULN if <LLN pretreatment, or <LLN if >ULN pretreatment

uric acid - >1.5x ULN; if pretreatment above ULN, >2x pretreatment value

urinalysis

a) urinary protein - >1 gram/24 hours and 2x pretreatment value

b) urinary RBC - >5/HPF or, >4x pretreatment if pretreatment value 5/HPF

c) urinary WBC - >5/HPF or, >4x pretreatment if 5/HPF pretreatment

d) creatinine clearance (glomerular filtration rate) - <0.67x pretreatment value

e) urine dipstick measurements: Protein, blood, sugar, and acetone- 2+, or if 1+pretreatment, 2x pretreatment. Do not evaluate protein if quantitative protein determination done

stool hemoccult - positive if negative pretreatment

Approved v7.0 930029550 5.0
