# Supplementary material for: Novel lung imaging biomarkers and skin gene expression subsetting in dasatinib treatment of systemic sclerosis-associated interstitial lung disease
Source: PLoS One. 2017 Nov 9;12(11):e0187580. doi: 10.1371/journal.pone.0187580 (PMC5679625; doi:10.1371/journal.pone.0187580)
Supplement: S1 Table — (DOCX) [file pone.0187580.s003.docx]

| Criteria | Target population |
| --- | --- |
| Inclusion criteria | - Meet ACR criteria for scleroderma - Have clinical evidence of active skin disease with a skin score of ≥15 - Have had the onset of their first non-Raynaud phenomenon feature of SSc no more than 3 years prior to screening - Have evidence of fibrosing alveolitis (active pulmonary fibrosis) manifested by a FVC between 45% and 80% of predicted normal and/or a DLCO between 30% and 70% of predicted normal - Have an abnormal HRCT scan of the chest/lungs demonstrating typical ground glass changes of alveolitis with background fibrosis - Have adequate renal function – no evidence of renal crisis in the 2 months prior to enrollment and serum creatinine < 3 mg/dL - For both sexes, must use an acceptable form of birth control - Men and women, ages ≥ 18 |
| Exclusion criteria | - Clinically significant pleural or pericardial effusion in the previous 12 months: Grade 3 or 4. Patients with recent Grade I or II effusions or peripheral edema will be permitted to enter the study - Clinically significant cardiac disease (New York Heart Association Class III or IV) including preexisting arrhythmia, (such as ventricular tachycardia, ventricular fibrillation, or "Torsade de Pointes"), myocardial infarction, uncontrolled angina within 6 months, congestive heart failure, cardiomyopathy, or pericardial disease - Clinically significant coagulation or platelet function disorder (e.g., known von Willebrand's disease) - Abnormal QTcF interval prolonged (> 450 msec) after electrolytes have been corrected on baseline electrocardiogram   Laboratory Test Findings   - Hgb < 10 g/dL; platelet count < 100,000/dL; WBC < 3,000/dL; PMN < 1,000/dL; OR lymphocytes < 350/dL - The presence of any of the following laboratory findings at screening: positive for antibodies to hepatitis C virus; positive for antibodies to hepatitis B surface antigen (HBsAg); serum bilirubin ≥ 2 times normal, Alanine Aminotransferase (ALT), or Aspartate Aminotransferase (AST)> 2.5 times upper limit of normal   Prohibited Treatments and/or Therapies   - Use of other immunosuppressive therapies must be discontinued at enrollment, eg methotrexate, azathioprine, cyclophosphamide, mycophenolic acid, mycophenolate mofetil, cyclosporine - Treatment with any other experimental or investigational drug(s) concurrently or less than 12 weeks prior to study enrollment - Use of anti-fibrotic agents must be discontinued at enrollment, e.g. colchicine, D‑penicillamine, minocycline or Type 1 oral collagen - Current or prior use of endothelin 1 receptors e.g. Bosentan, prior exposure to dasatinib, imatinib, or any other tyrosine kinase inhibitor. |
