# Supplementary material for: Novel lung imaging biomarkers and skin gene expression subsetting in dasatinib treatment of systemic sclerosis-associated interstitial lung disease
Source: PLoS One. 2017 Nov 9;12(11):e0187580. doi: 10.1371/journal.pone.0187580 (PMC5679625; doi:10.1371/journal.pone.0187580)
Supplement: S2 Table — (DOCX) [file pone.0187580.s004.docx]

| Adverse events | Overall, n (%) | Dasatinib-related, n (%) |
| --- | --- | --- |
| Any adverse event | 31 (100) | 24 (77.4) |
| Diarrhea | 12 (38.7) | 4 (12.9) |
| Fatigue | 12 (38.7) | 1 (3.2) |
| Constipation | 8 (25.8) | 1 (3.2) |
| Nausea | 8 (25.8) | 4 (12.9) |
| Vomiting | 8 (25.8) | 4 (12.9) |
| Cough | 8 (25.8) | 1 (3.2) |
| Headache | 8 (25.8) | 5 (16.1) |
| Peripheral edema | 6 (19.4) | 6 (19.4) |
| Rash | 6 (19.4) | 2 (6.5) |
| Anemia | 6 (19.4) | 1 (3.2) |
| Dyspnea | 5 (16.1) | 2 (6.5) |
| Arthralgia | 5 (16.1) | 1 (3.2) |
| Insomnia | 5 (16.1) | 0 |
| Pleural effusion | 4 (12.9) | 3 (9.7) |
| Skin ulcer | 4 (12.9) | 0 |
| Sinusitis | 4 (12.9) | 0 |
| Upper respiratory tract infection | 4 (12.9) | 0 |
| Urinary tract infection | 4 (12.9) | 1 (3.2) |
| Dizziness | 4 (12.9) | 1 (3.2) |
| Depression | 4 (12.9) | 0 |
