# Supplementary material for: Novel lung imaging biomarkers and skin gene expression subsetting in dasatinib treatment of systemic sclerosis-associated interstitial lung disease
Source: PLoS One. 2017 Nov 9;12(11):e0187580. doi: 10.1371/journal.pone.0187580 (PMC5679625; doi:10.1371/journal.pone.0187580)
Supplement: S3 Table — (DOCX) [file pone.0187580.s005.docx]

| Serious adverse events | Overall,  n (%) | Dasatinib-related,  n (%) |
| --- | --- | --- |
| Any serious adverse event | 7 (22.6) | 5 (16.1) |
| Grade 4 |  |  |
| Myocardial infarction | 1 (3.2) | no |
| Hypoxia | 1 (3.2) | yes |
| Fluid overload | 1 (3.2) | yes |
| Grade 3 |  |  |
| Pleural effusion | 1 (3.2) | yes |
| Gastrointestinal hemorrhage | 1 (3.2) | no |
| Hematemesis | 1 (3.2) | yes |
| Clostridial infection | 1 (3.2) | no |
| Grade 1 |  |  |
| Atrial flutter | 1 (3.2) | no |
| Diastolic dysfunction | 1 (3.2) | yes |
| Dyspnea | 1 (3.2) | yes |
| Cystitis | 1 (3.2) | no |
| Face edema | 1 (3.2) | yes |
