# Supplementary material for: Novel lung imaging biomarkers and skin gene expression subsetting in dasatinib treatment of systemic sclerosis-associated interstitial lung disease
Source: PLoS One. 2017 Nov 9;12(11):e0187580. doi: 10.1371/journal.pone.0187580 (PMC5679625; doi:10.1371/journal.pone.0187580)
Supplement: S4 Table — (DOCX) [file pone.0187580.s006.docx]

| HRCT | PFT | | Serum markers | | | MRSS  (n=27) |
| --- | --- | --- | --- | --- | --- | --- |
|  | % predicted D_L_CO  (n=30) | % predicted FVC  (n=30) | APRIL (n=28) | SP-D  (n=28) | KL-6  (n=27) |  |
| Most severe lobe  QLF  QILD* | –0.52  (0.0033)  –0.51  (0.0050) | –0.54  (0.0020)  –0.52  (0.0038) | –0.05 (0.78)  0.02  (0.92) | 0.67  (0.0001)  0.73  (<0.0001) | 0.58  (0.0016)  0.59 (0.0016) | 0.134  (0.50)  0.078  (0.71) |
| Whole lung  QLF  QILD | –0.51  (0.0043)  –0.51  (0.0045) | –0.53  (0.0025)  –0.48  (0.0077) | 0.0249  (0.90)  0.0440  (0.83) | 0.68  (0.0001)  0.73  (<0.0001) | 0.61  (0.0007)  0.61  (0.0009) | 0.10 (0.60)  0.12 (0.57) |

*Sample size for QILD is 29.

Correlation values are reported in the following format: Spearman’s rho on top; (associated p-value at the bottom).
