# Supplementary material for: Novel lung imaging biomarkers and skin gene expression subsetting in dasatinib treatment of systemic sclerosis-associated interstitial lung disease
Source: PLoS One. 2017 Nov 9;12(11):e0187580. doi: 10.1371/journal.pone.0187580 (PMC5679625; doi:10.1371/journal.pone.0187580)
Supplement: S5 Table — (DOCX) [file pone.0187580.s007.docx]

| Change  in CAD score | Change  in PFT | | Change  in serum markers | | | Change  in MRSS  (n=19) |
| --- | --- | --- | --- | --- | --- | --- |
|  | % predicted D_L_CO  (n=20) | % predicted FVC  (n=22) | APRIL (n=22) | SP-D  (n=22) | KL-6  (n=22) |  |
| Most severe lobe  QLF  QILD* | –0.45  (0.0479)  –0.69  (0.0001) | –0.38  (0.08)  –0.32  (0.16) | 0.078  (0.73)  0.32  (0.16) | 0.59  (0.0035)  0.39 (0.079) | –0.05 (0.83)  0.14  (0.54) | 0.18  (0.45)  –0.06  (0.81) |
| Whole lung  QLF  QILD* | –0.39  (0.09)  –0.49  (0.032) | –0.36  (0.10)  –0.56  (0.0083) | 0.24  (0.28)  0.42  (0.058) | 0.47  (0.029)  0.22  (0.34) | –0.15  (0.51)  –0.09  (0.68) | 0.08  (0.73)  –0.19  (0.44) |

*Sample size for QILD is 21. Honeycomb HRCT feature was not evaluable.

Correlation values are reported in the following format: Spearman’s rho on top; (associated p-value at the bottom).

APRIL, A B cell proliferation-inducing ligand; D_L_CO, diffusing capacity for carbon monoxide; FVC, forced vital capacity; KL-6, Krebs von den Lungen-6; SP-D, surfactant protein D; QLF, quantitative lung fibrosis score; QILD, quantitative interstitial lung disease score; CAD, computer-assisted diagnosis.
