# Supplementary material for: Novel lung imaging biomarkers and skin gene expression subsetting in dasatinib treatment of systemic sclerosis-associated interstitial lung disease
Source: PLoS One. 2017 Nov 9;12(11):e0187580. doi: 10.1371/journal.pone.0187580 (PMC5679625; doi:10.1371/journal.pone.0187580)
Supplement: S6 Table — (DOCX) [file pone.0187580.s008.docx]

| Adiponectin | APRIL | SP-D | KL-6 | FVC | D_L_CO | FEV_1_ | TLC |
| --- | --- | --- | --- | --- | --- | --- | --- |
| Total | 0.111  (0.602) | -0.158  (0.458) | -0.179  (0.402) | -0.251  (0.225) | -0.127  (0.551) | -0.323  (0.114) | 0.018  (0.934) |
| HMW | 0.043  (0.838) | -0.092  (0.668) | -0.153  (0.472) | -0.233  (0.261) | -0.122  (0.566) | -0.318  (0.120) | 0.032  (0.887) |
| Ratio | -0.003  (0.988) | 0.097  (0.649) | 0.02  (0.915) | -0.164  (0.433) | -0.162  (0.448) | -0.24  (0.246) | -0.047  (0.838) |

Correlation values are reported in the following format: Spearman’s rho on top; (associated p-value at the bottom).
