# Supplementary material for: Novel lung imaging biomarkers and skin gene expression subsetting in dasatinib treatment of systemic sclerosis-associated interstitial lung disease
Source: PLoS One. 2017 Nov 9;12(11):e0187580. doi: 10.1371/journal.pone.0187580 (PMC5679625; doi:10.1371/journal.pone.0187580)
Supplement: S7 Table — (DOCX) [file pone.0187580.s009.docx]

| Δ Adiponectin | Δ APRIL | Δ SP-D | Δ KL-6 | Δ FVC | Δ D_L_CO | Δ FEV_1_ | Δ TLC |
| --- | --- | --- | --- | --- | --- | --- | --- |
| Δ Total | -0.105  (0.624) | -0.297  (0.158) | -0.198  (0.352) | 0.134  (0.584) | 0.242  (0.332) | 0.259  (0.283) | 0.258  (0.316) |
| Δ HMW | -0.085  (0.692) | -0.240  (0.257) | 0.220  (0.301) | 0.167  (0.493) | -0.003  (0.987) | 0.346  (0.146) | 0.149  (0.567) |
| Δ Ratio | 0.016  (0.939) | 0.128  (0.549) | 0.840  (<0.001) | -0.004  (0.984) | -0.229  (0.359) | 0.212  (0.383) | -0.123  (0.637) |

APRIL, A B cell proliferation-inducing ligand; D_L_CO, diffusing capacity for carbon monoxide; FEV_1_, forced expiratory volume in 1 sec; HMW, high molecular weight; ratio = HMW/total adiponectin; FVC, forced vital capacity; KL-6, Krebs von den Lungen-6; SP-D, surfactant protein D.

Correlation values are reported in the following format: Spearman’s rho on top; (associated p-value at the bottom).
