# Supplementary material for: Novel lung imaging biomarkers and skin gene expression subsetting in dasatinib treatment of systemic sclerosis-associated interstitial lung disease
Source: PLoS One. 2017 Nov 9;12(11):e0187580. doi: 10.1371/journal.pone.0187580 (PMC5679625; doi:10.1371/journal.pone.0187580)
Supplement: S9 Table — (DOCX) [file pone.0187580.s011.docx]

| Patient ID | Baseline MRSS | Follow-up MRSS | ΔMRSS | %ΔMRSS | Response* | Baseline  intrinsic subset |
| --- | --- | --- | --- | --- | --- | --- |
| 1 | 26 | 20 | -6 | -23.1% | Improver | Normal-like |
| 2 | 28 | 17 | -11 | -39.3% | Improver | Normal-like |
| 3 | 25 | 16 | -9 | -36.0% | Improver | Fibroproliferative |
| 4 | 48 | 46 | -2 | -4.2% | Non-improver | Inflammatory |
| 5 | 22 | 31 | 9 | 40.9% | Non-improver | Inflammatory |
| 6 | 21 | 36 | 15 | 71.4% | Non-improver | Inflammatory |
| 7 | 23 | 21 | -2 | -8.7% | Non-improver | Normal-like |
| 8 | 40 | 38 | -2 | -5.0% | Non-improver | Inflammatory |
| 9 | 20 | 27 | 7 | 35.0% | Non-improver | Inflammatory |
| 10 | 20 | 17 | -3 | -15.0% | Non-improver | Inflammatory |
| 11 | 21 | 20 | -1 | -4.8% | Non-improver | Inflammatory |
| 12 | 22 | 23 | 1 | 4.5% | Non-improver | Fibroproliferative |

*Clinical response defined as >5 point or >20% decrease in MRSS at follow-up
