# Supplementary material for: Novel lung imaging biomarkers and skin gene expression subsetting in dasatinib treatment of systemic sclerosis-associated interstitial lung disease
Source: PLoS One. 2017 Nov 9;12(11):e0187580. doi: 10.1371/journal.pone.0187580 (PMC5679625; doi:10.1371/journal.pone.0187580)
Supplement: S10 Table — (DOCX) [file pone.0187580.s012.docx]

| Chest HRCT in whole lung | Patients | | | |
| --- | --- | --- | --- | --- |
|  | Normal-like  (n=3) | Inflammatory  (n=7*) | Fibroproliferative (n=2) | Total |
| Baseline, %  QLF  QILD  QGG-QLF | 6.9 ± 5.8  24.8 ± 18.5  10.9 ± 9.4 | 5.8 ± 5.8  26.5 ± 12.9  14.7 ± 5.3 | 5.5 ± 7.3  18.1 ± 17.2  7.0 ± 2.7 | 6.0 ± 5.4  24.5 ± 13.8  12.5 ± 6.4 |
| Change at day 169 vs. baseline, %  QLF  QILD  QGG-QLF | 0.8 ± 0.9  2.3 ± 2.7  12.5 ± 15.7 | 6.2 ± 9.0  5.7 ± 8.6  3.9 ± 14.9 | 0.2 ± 0.0  -2.1 ± 3.8  3.9 ± 0.3 | 3.1 ± 6.2  2.5 ± 6.0  6.8 ± 12.8 |

^*^At day 169, four subjects were available from inflammatory group.

Values are mean ± standard deviation.
