# Supplementary figures and images for: Novel lung imaging biomarkers and skin gene expression subsetting in dasatinib treatment of systemic sclerosis-associated interstitial lung disease
Source: PLoS One. 2017 Nov 9;12(11):e0187580. doi: 10.1371/journal.pone.0187580 (PMC5679625; doi:10.1371/journal.pone.0187580)

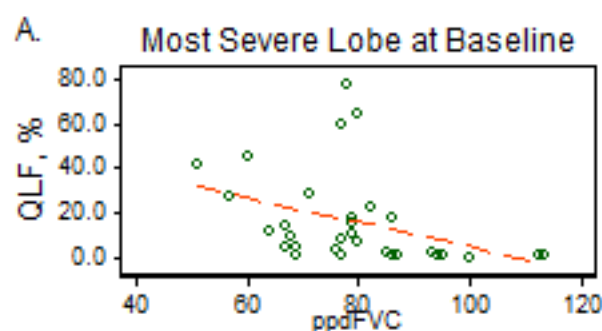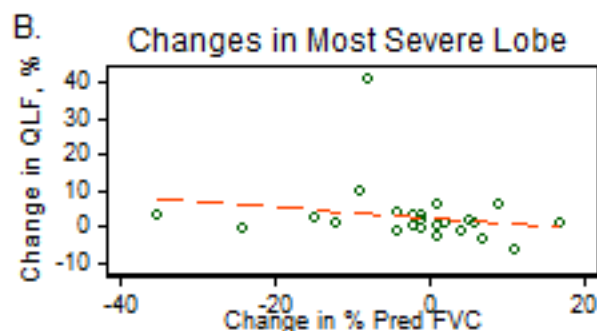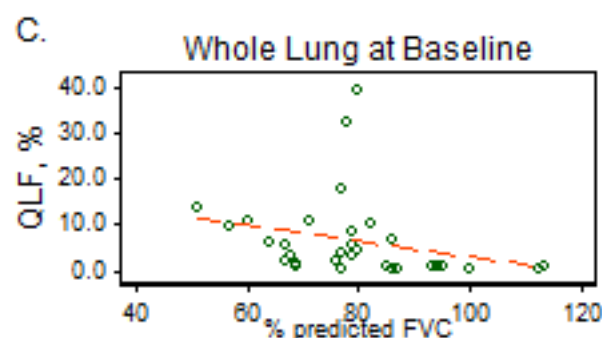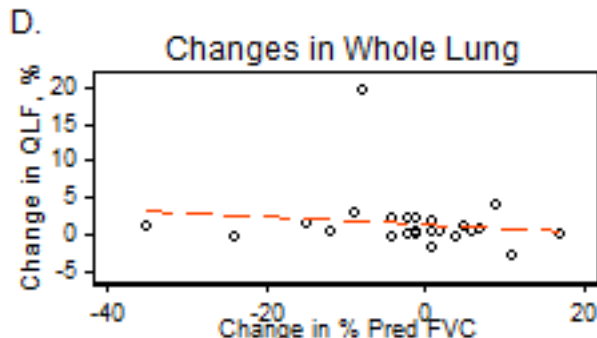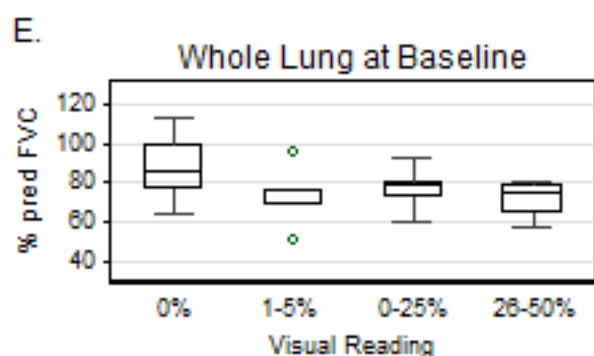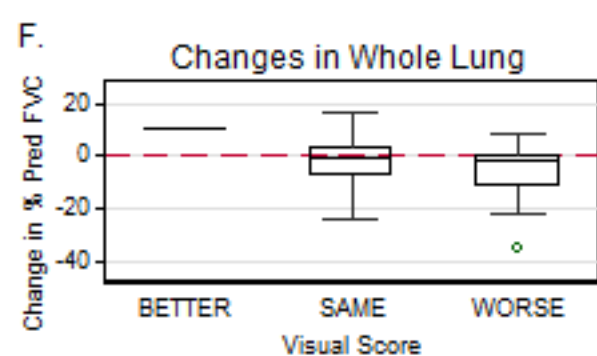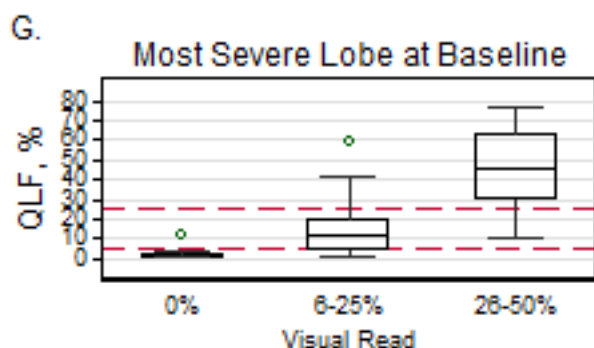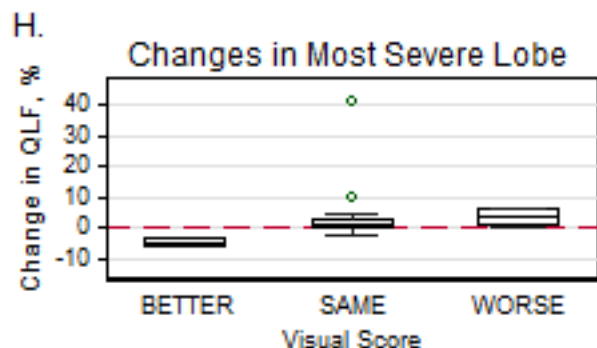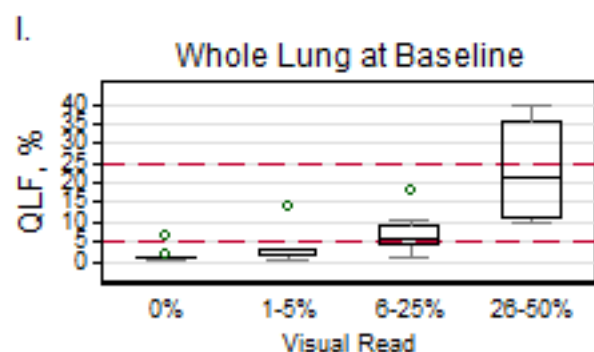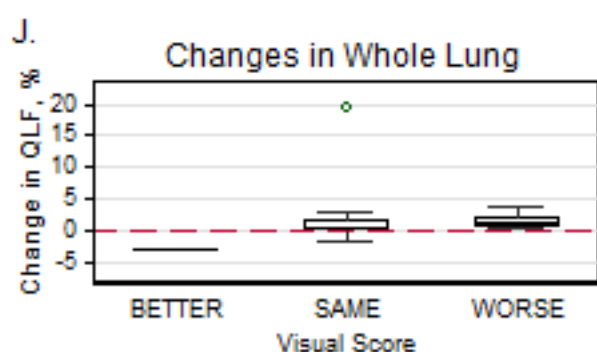

Supplement: S1 Fig — (A) Plot of FVC (% predicted) and QLF score at the most severe lobe. (B) Plot of changes in FVC and QLF score at the most severe lobe at baseline. (C) Plot of FVC and QLF score in whole lung at baseline. (D) Plot of changes in FVC and QLF score in whole lung. (E) Box-whisker plot of FVC by visually assessed whole lung fibrotic reticulation at baseline. The range of visual assessment is as follows: 0% (none), 1–5%, 6–25%, 26–50%, 51–75%, and >75%. (F) Box-whisker plot of changes in FVC by the changes of the visual assessment in fibrotic reticular pattern (better, same, worse) in whole lung; (G) Box-whisker plot of QLF score by the visual assessment of fibrotic reticulation at the most severe lobe at baseline. (H) Box-whisker plot of changes in QLF by the visual assessment of fibrotic reticulation at the most severe lobe. (I) Box-whisker plot of QLF score by the visual assessment of fibrotic reticulation in whole lung at baseline. (J) Box-whisker plot of changes in QLF by the changes of the visual assessment in fibrotic reticular pattern (better, same, worse) in whole lung. (PDF) [file pone.0187580.s013.pdf]

**A**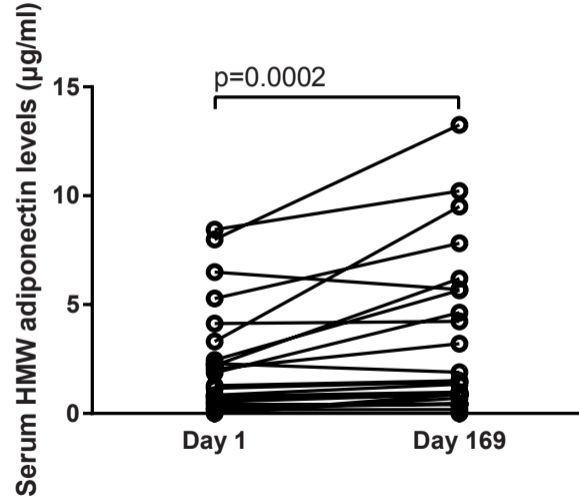**B**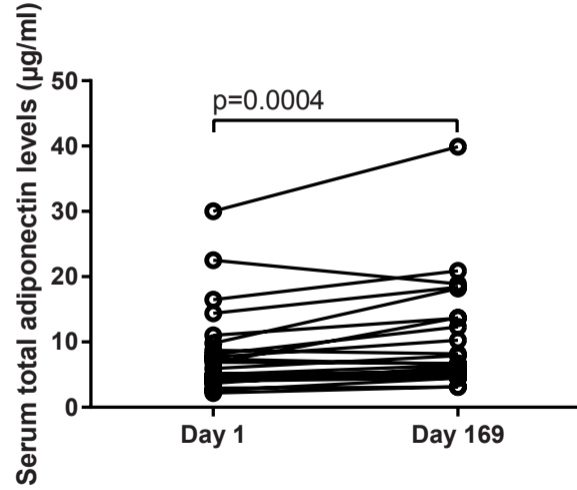**C**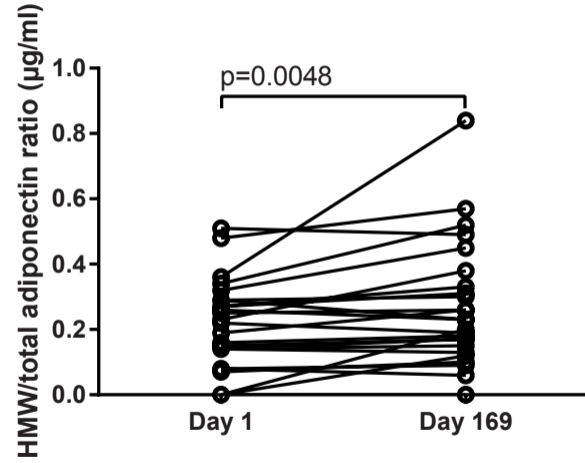

Supplement: S2 Fig — Change in levels of high molecular weight (HMW) adiponectin (A), total adiponectin (B), and ratio of HMW/total adiponectin (C). Data represent mean of duplicates for each patient at day 1 and 169 (n = 25). (PDF) [file pone.0187580.s014.pdf]

Expression values  
for centroids

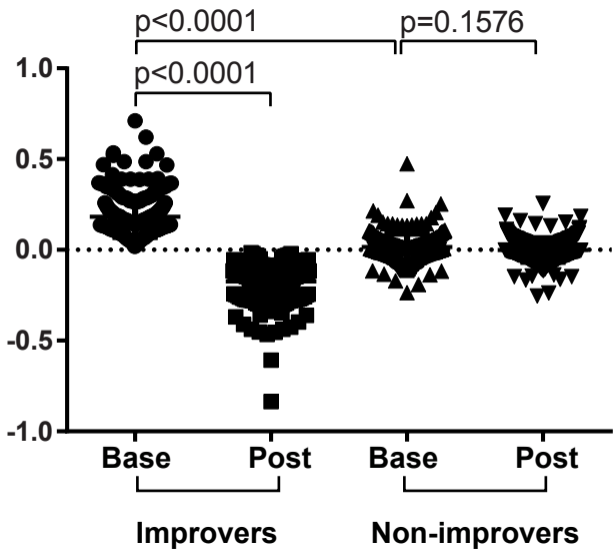

Supplement: S3 Fig — Only the centroid for genes down-regulated post-treatment in improvers is shown; the centroid for genes upregulated post-treatment represents its mirror image. See Statistical analysis section for the centroid explanation. P-values are for Tukey’s multiple comparisons test following ordinary one-way analysis of variance (ANOVA). Data are displayed as mean ± SEM scatter plot. (PDF) [file pone.0187580.s015.pdf]
